# Annulative π-extension of indoles and pyrroles with diiodobiaryls by Pd catalysis: rapid synthesis of nitrogen-containing polycyclic aromatic compounds†
†Electronic supplementary information (ESI) available: Syntheses, NMR, UV-vis-nearIR absorption, CV and crystallographic table. CCDC 1848311 (**3ad**), 1848309 (**8**), 1848310 (**9**). For ESI and crystallographic data in CIF or other electronic format see DOI: 10.1039/c8sc02802h


**DOI:** 10.1039/c8sc02802h

**Published:** 2018-08-09

**Authors:** Hiroyuki Kitano, Wataru Matsuoka, Hideto Ito, Kenichiro Itami

**Affiliations:** a Institute of Transformative Bio-Molecules (WPI-ITbM) , Nagoya University , Chikusa , Nagoya 464-8602 , Japan; b Graduate School of Science , Nagoya University , Chikusa , Nagoya 464-8602 , Japan; c JST-ERATO , Itami Molecular Nanocarbon Project , Nagoya University , Chikusa , Nagoya 464-8602 , Japan . Email: ito.hideto@g.mbox.nagoya-u.ac.jp ; Email: itami@chem.nagoya-u.ac.jp

## Abstract

A palladium-catalyzed one-step annulative π-extension (APEX) reaction of indoles and pyrroles that allows rapid access to nitrogen-containing polycyclic aromatic compounds is described. In the presence of palladium pivalate and silver carbonate, diverse indoles or pyrroles coupled with diiodobiaryls in a double direct C–H arylation manner to be transformed into the corresponding π-extended compounds in a single step. The newly developed catalytic system enables the use of various pyrroles and indoles as templates with a series of diiodobiaryls to provide structurally complicated and largely π-extended nitrogen-containing polycyclic aromatic compounds that are otherwise difficult to synthesize.

## Abstract 2

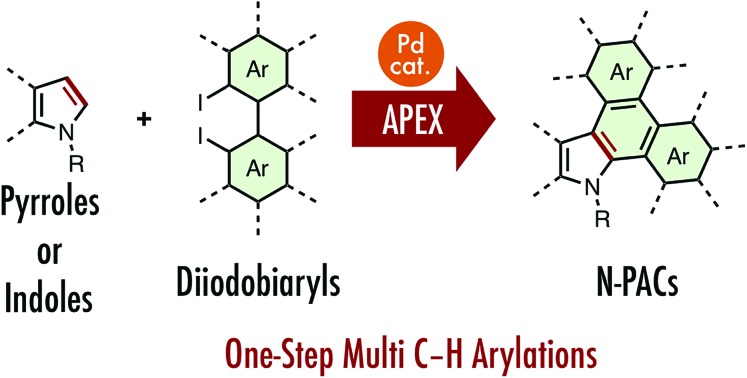
A palladium-catalyzed one-step annulative π-extension (APEX) reaction of indoles and pyrroles that allows rapid access to nitrogen-containing polycyclic aromatic compounds is described.

## Introduction

With desirable electronic properties and diverse biological activities, nitrogen-containing fused aromatics have long been recognized as privileged structures in the fields of organic materials and pharmaceutical science.^1^ As these properties can be readily tuned *via* skeletal modification of the core *N*-heteroarene structure, significant efforts have been devoted to develop new synthetic approaches for π-extended nitrogen-containing polycyclic aromatic compounds (N-PACs). Representative classical approaches include (i) intramolecular carbon–nitrogen bond formation of biaryl amines,^2^ (ii) intramolecular carbon–carbon bond formation of diaryl amines,^3^ and (iii) stepwise functionalization and π-extension of indoles and pyrroles.^4^ However, these methods require the use of prefunctionalized heteroaromatics such as halogenated pyrroles, anilines and indoles, and stepwise transformations from unfuctionalized (hetero)aromatics. To achieve maximum efficiency in N-PAC construction, a more direct and ‘intuitive’ method for π-extension of unfunctionalized pyrroles and indoles is called for.

**Fig. 1:**
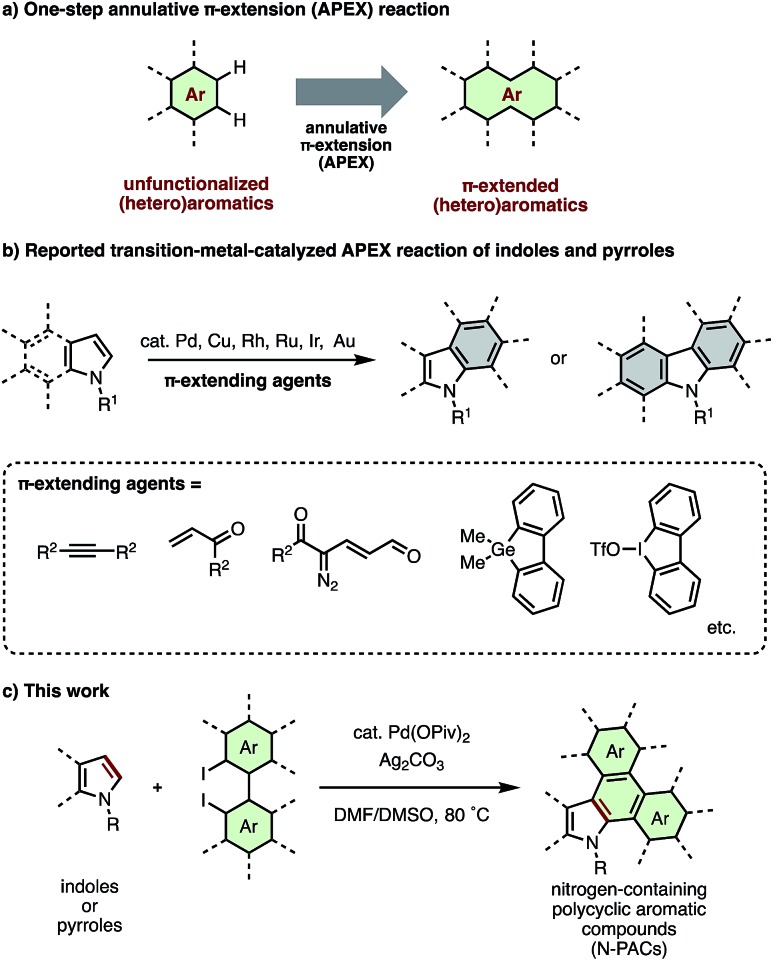
(a) General scheme of annulative π-extension (APEX) reaction of unfunctionalized (hetero)aromatics. (b) Previous transition-metal-catalyzed APEX reactions of indoles and pyrroles. (c) Palladium-catalyzed APEX reaction of indoles and pyrroles with diiodobiaryls (this work).

Recently, we have introduced several new one-step methods for the annulative π-extension (APEX) of unfuctionalized (hetero)aromatics (Fig. 1a).^5^–^7^ Because such APEX reactions directly transform easily available unfunctionalized (hetero)arenes to polycyclic aromatic hydrocarbons, nanographenes and π-extended heteroaromatics in a double direct C–H arylation manner, these protocols offer large benefits in the context of cost, simplicity, and step/atom economy.^8^

Recently, we^7^ and others^9^–^14^ have reported transition-metal-catalyzed APEX reactions of indoles and pyrroles using various π-extension units such as alkyne,^9^ alkene,7a,^10^ 1-vinylpropargyl alcohols,^11^ α-diazocarbonyl compounds,^12^ α-bromochalcone,^13^ α-bromocinnamate,^13^ cyclic diaryliodonium salts,^14^ dibenzogermoles7*b* and diiodobiphenyls7*c* (Fig. 1b). However, these APEX reactions are limited in terms of lack of variety in π-extending agents, narrow substrate scope, and low functional group tolerance. Herein, we report a new catalytic APEX reaction that allows efficient pyrrole-to-indole, pyrrole-to-carbazole and indole-to-carbazole π-extensions. Our newly established catalytic system featuring palladium pivalate and silver carbonate in a mixed DMF/DMSO solvent system enabled the rapid synthesis of structurally complicated N-PACs from readily available unfunctionalized pyrroles/indoles and diiodobiaryls.

## Results and discussion

We began our study by optimizing the reaction conditions for indole-to-carbazole extension of *N*-methylindole (**1a**) using 2,2′-diido-1,1′-biphenyl (**2a**) as a π-extending agent (Table 1). After extensive screening, we discovered that **1a** (1.0 equiv.) coupled with **2a** (1.5 equiv.) in the presence of Pd(OAc)_2_ (5 mol%) and Ag_2_CO_3_ (3.0 equiv.) at 80 °C in 7 : 3 mixture of dimethylformamide (DMF) and dimethylsulfoxide (DMSO) to provide *N*-methyldibenzo[*a*,*c*]carbazole (**3aa**) in 54% yield (entry 1). Use of palladium pivalate [Pd(OPiv)_2_] instead of Pd(OAc)_2_ improved the yield to 61% (entry 2), but other palladium sources such as PdCl_2_, PdI_2_, Pd(PPh_3_)_4_, Pd(OCOCF_3_)_2_ and Pd(CH_3_CN)_4_(BF_4_)_2_ failed to give more than trace amounts of product (entries 3–7). Decreasing the amount of Ag_2_CO_3_ to 1.5 equiv. (relative to **1a**) further increased the yield of **3aa** to 78% (entry 8). The use of silver carboxylate salts (AgOAc, AgOPiv, or AgOCOCF_3_) instead of Ag_2_CO_3_ resulted in much lower yield (entries 9–11). The silver cation itself was essential for this reaction; the use of Na_2_CO_3_, K_2_CO_3_ or Cs_2_CO_3_ instead of Ag_2_CO_3_ failed to give any product (see ESI† for details). The use of the DMF/DMSO mixed solvent system was important for obtaining maximum conversion; highly polar single solvents such as *N*,*N*-dimethylacetamide (DMAc), DMF, DMSO, CH_3_CN provided **3aa** in diminished yield (29–10%, see ESI† for details), while less polar solvents such as 1,2-dichloroethane, 2,2,2-trifluoroethanol, 1,4-dioxane and toluene completely suppressed the reaction. Although higher reaction temperature accelerated the consumption of the starting material, the yield of **3aa** was decreased (entries 12 and 13). Finally, we confirmed that the APEX reaction did not proceed in the absence of Pd catalyst or Ag_2_CO_3_ (entries 14 and 15). Although the use of additional ligands for Pd and the use of dibromobiphenyl in place of diiodobiphenyl as the π-extension reagent were also investigated, these modifications proved ineffective (see ESI† for details). Ultimately the conditions in entry 8 were deemed optimal for the present indole-to-carbazole APEX reaction.

**Table 1:** Screening of reaction conditions for the Pd-catalyzed indole-to-carbazole APEX reaction of *N*-methylindole (**1a**) and diiodobiphenyl (**2a**)

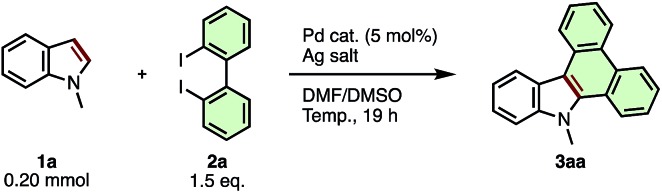				
Entry	Pd cat.	Ag salt	Temp. (°C)	Yield^*a*^ (%)
1	Pd(OAc)_2_	Ag_2_CO_3_ (3.0 eq.)	80	54
2	Pd(OPiv)_2_	Ag_2_CO_3_ (3.0 eq.)	80	61
3	PdCl_2_	Ag_2_CO_3_ (3.0 eq.)	80	3
4	PdI_2_	Ag_2_CO_3_ (3.0 eq.)	80	2
5	Pd(PPh_3_)_4_	Ag_2_CO_3_ (3.0 eq.)	80	4
6	Pd(OCOCF_3_)_2_	Ag_2_CO_3_ (3.0 eq.)	80	0
7	Pd(CH_3_CN)_4_(BF_4_)_2_	Ag_2_CO_3_ (3.0 eq.)	80	0
8	Pd(OPiv)_2_	Ag_2_CO_3_ (1.5 eq.)	80	78 (66)^*c*^
9	Pd(OPiv)_2_	AgOAc (3.0 eq.)	80	33
10	Pd(OPiv)_2_	AgOPiv (3.0 eq.)	80	0
11	Pd(OPiv)_2_	AgOCOCF_3_ (3.0 eq.)	80	0
12	Pd(OPiv)_2_	Ag_2_CO_3_ (1.5 eq.)	100	57
13^*b*^	Pd(OPiv)_2_	Ag_2_CO_3_ (1.5 eq.)	100	58
14	None	Ag_2_CO_3_ (1.5 eq.)	80	0
15	Pd(OPiv)_2_	None	80	0

^*a*^Yield was determined by ^1^H NMR analysis using dodecane as an internal standard.

^*b*^Reaction time was 1 h.

^*c*^Isolated yield in the parenthesis. Piv = pivaloyl.

**Scheme 1:**
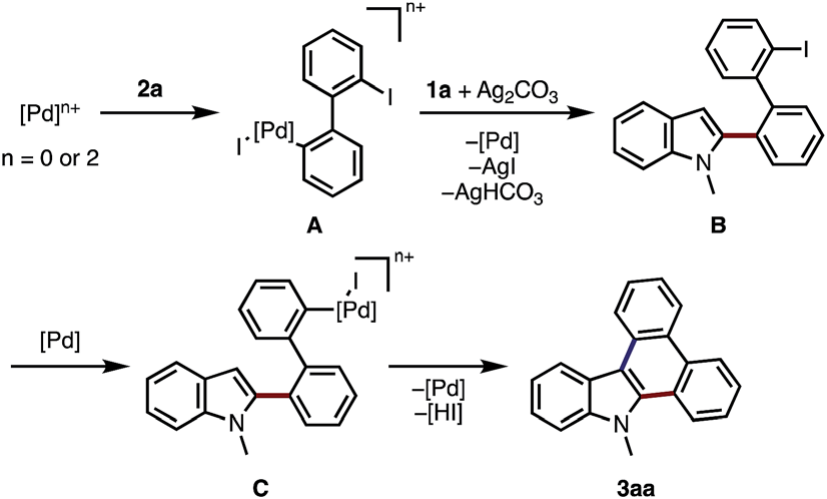
Proposed reaction mechanism for the Pd-catalyzed APEX reaction of *N*-methylindole (**1a**) with 2,2′-diiodo-1,1′-biphenyl (**2a**).

A possible reaction mechanism of current indole-to-carbazole APEX reaction is shown in Scheme 1. Oxidative addition of **2a** to palladium species (Pd(0) or Pd(ii)) occurs to form biphenylylpalladium intermediate **A**.^15^ Then, the removal of iodide by silver salt may activate Pd complex **A**^16^ to form electron-deficient aryl-Pd species,^17^ which then react with indole at the C2 position to afford intermediate **B**. Through the control experiments on the C–H arylations of 1,2-dimethylindole and 1,3-dimethylindole with iodobenzene, the present APEX reaction seems to occur through the C2-arylation of indole rather than C3-arylation in the first step (see ESI† for details). Final step would be well-established Pd-catalyzed intramolecular C–H/C–I coupling to afford the cyclized compound **3aa**.^18^

**Scheme 2:**
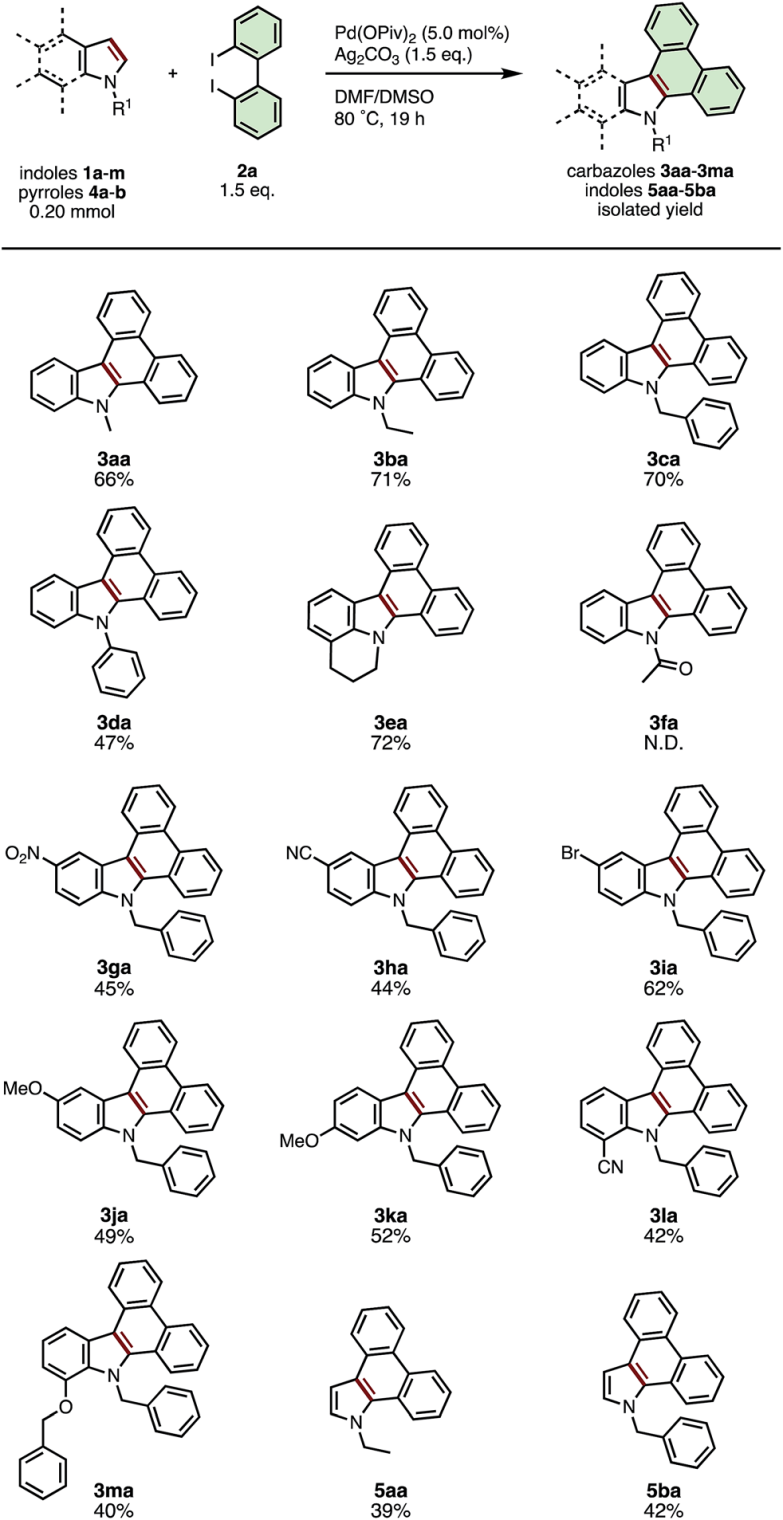
Substrate scope of indoles and pyrroles in the APEX reaction with 2,2′-diiodo-1,1′-biphenyl (**2a**).

Under the optimized conditions, various types of π-extended carbazoles/indoles **3**, **5** were prepared from the corresponding indole/pyrrole derivatives **1**, **4** and diiodobiaryls **2**. Scheme 2 illustrates the scope of applicable indole and pyrrole derivatives (**1a–1m**). *N*-Alkyl (**2a**, **2b**), *N*-benzyl (**2c**), *N*-phenyl (**2d**) indoles and cross-linked lilolidine (**2e**) were converted smoothly to dibenzocarbazoles **3ba–3da** in good to moderate yield, however the reaction of *N*-acetyl indole **2f** did not provide the expected π-extension product **3fa**. The presence of substituents at the 5-, 6-, or 7-positions of the indole ring was well-tolerated, giving various nitro- (**3ga**), cyano- (**3ha**, **3la**), bromo- (**3ia**), methoxy- (**3ja**, **3ka**), and benzyloxy-substituted (**3ma**) dibenzocarbazoles in moderate yields (40–62%). These results suggest that substituents on the benzene ring of indole do not critically affect the reaction progress. Interestingly, we found that the current APEX reaction between *N*-substituted pyrroles and **2a** was mono-selective for the formation of dibenzoindoles **5aa** and **5ba** in 39% and 43% yields; only trace amounts of the di-APEX tetrabenzocarbazole products, the main products of our previous report,7c,^19^ were observed. As synthetic methods to prepare the dibenzo[*e*,*g*]indole skeleton remain limited and inefficient,^20^ the current APEX protocol provides a valuable, streamlined entry to this compound class.

**Scheme 3:**
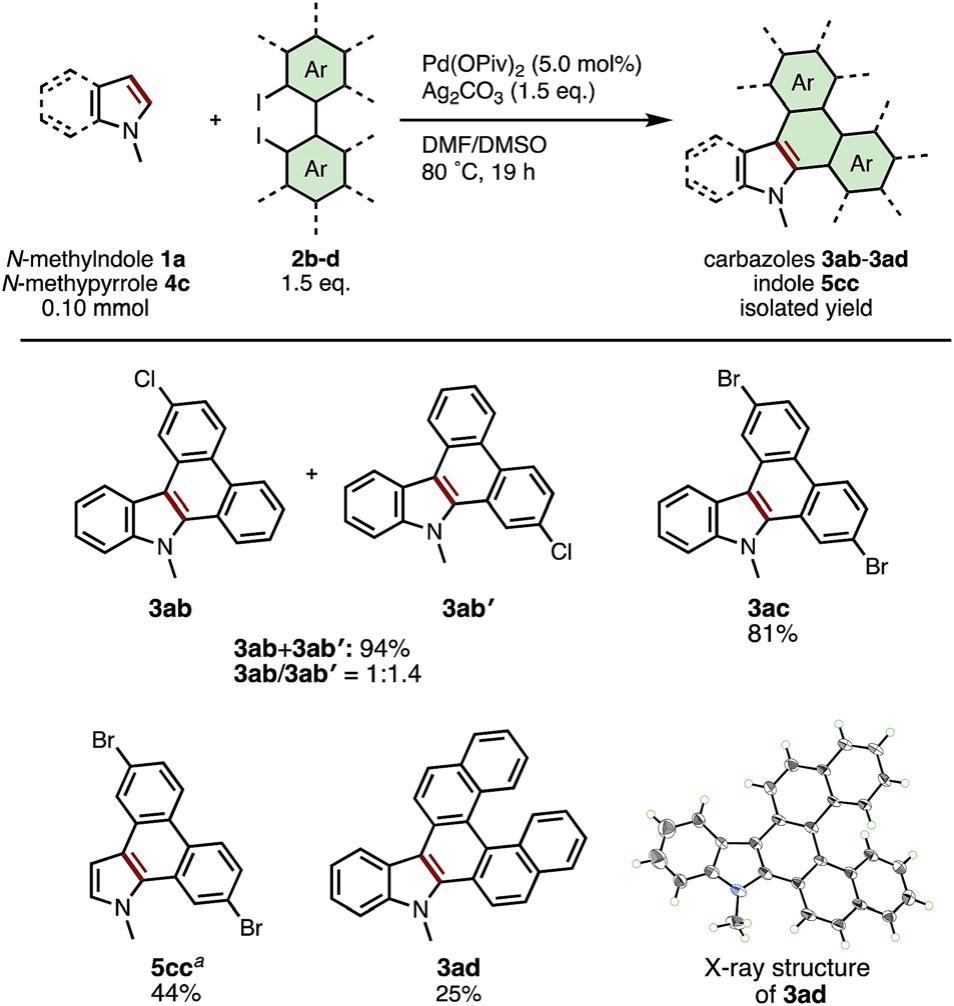
Substrate scope of diiodobiaryls. ^*a*^0.20 mmol scale.

The scope of diiodobiaryls in the current APEX reaction is shown in Scheme 3. The reaction of *N*-methylindole (**2a**) with unsymmetrical 4-chloro-2,2′-diiodo-1,1′-biphenyl (**2b**) gave a 1 : 1.4 regioisomeric mixture of **3ab** and **3ab′** in 94% combined yield. APEX reactions of **1a** and **4a** with 4,4′-dibromo-2,2′-diiodo-1,1′-biphenyl (**2c**) smoothly occurred to give dibromodibenzocarbazole **3ac** and dibromodibenozoindole **5cc** in 81% and 44% yield, respectively. To our delight, the reaction of **1a** with 2,2′-diiodo-1,1′-binaphthalene (**2d**) gave dinaphthocarbazole **3ad** containing a [5]helicene moiety in 25% yield, whose helical structure was confirmed by X-ray crystallographic analysis. As this example clearly demonstrates, the late-stage attachment of complex, extended polyaromatic units is one of the most remarkable characteristics in the present APEX reaction.

**Scheme 4:**
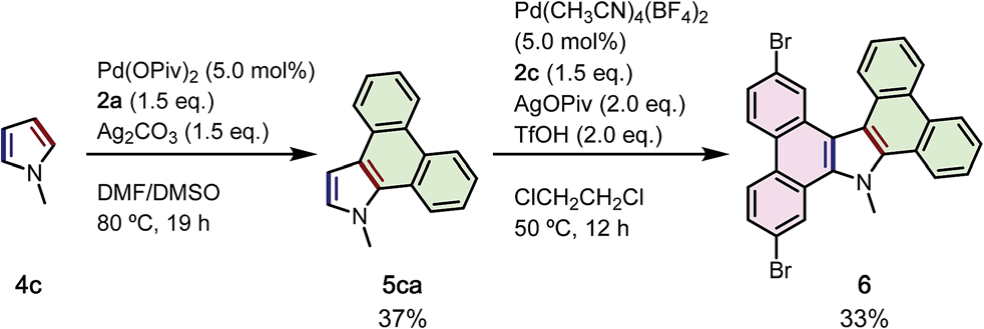
Sequential APEX reactions of *N*-methylpyrrole (**4c**) for the synthesis of unsymmetrically substituted tetrabenzocarbazole **6**.

To demonstrate the power of the current APEX reaction to build complex, unsymmetrical N-PACs from simple starting materials, we employed a two-step sequence to synthesize tetrabenzocarbazole **6**, a compound difficult to prepare *via* known methods (Scheme 4). First, APEX reaction of *N*-methylpyrrole (**4c**) with **2a** was carried out to give the corresponding *N*-methyldibenzoindole (**5ca**) in 37% yield. Notably, this reaction did not give double-APEX product which is the major product in the previously developed APEX reaction of *N*-phenylpyrrole.8*b* Then, **5ca** was further reacted with 4,4′-dibromo-2,2′-diiodo-1,1′-biphenyl (**2c**) by using Pd(CH_3_CN)_4_(BF_4_)_2_/AgOPiv/TfOH catalytic system8*b* to give the desired product **6** in 33% yield.^21^ Rapid access to a new class of unsymmetrically substituted tetrabenzocarbazole is notable, and should contribute to the exploration of new compounds for organic electronics application.

**Scheme 5:**
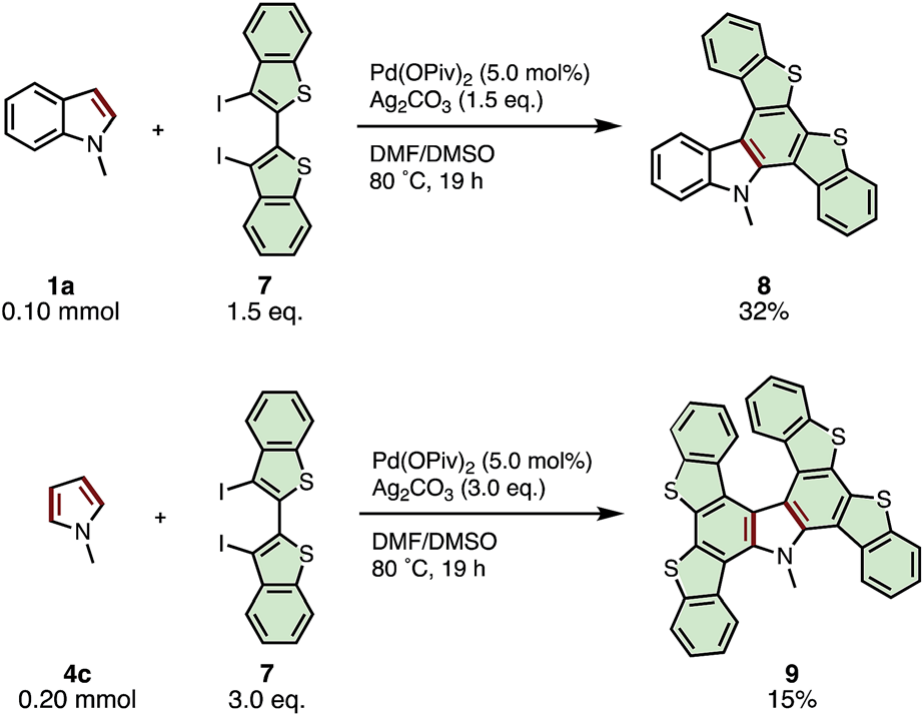
APEX reactions of *N*-methylindole (**1a**) and *N*-methylpyrrole (**4c**) with 3,3′-diiodo-2,2′-bibenzothiophene (**7**) for the synthesis of N–S-PACs.

The current APEX reaction also provided a facile route to polycyclic aromatic compounds containing both nitrogen and sulfur (N–S-PACs) (Scheme 5). *N*-Methylindole (**1a**) coupled with 3,3′-diiodo-2,2′-bibenzothiophene (**7**) to give di(benzothieno)carbazole **8** in 32%. To our delight, the reaction of *N*-methylpyrrole (**4c**) with diiodo-2,2′-bibenzothiophene **7** afforded a double APEX product, tetra(benzothieno)carbazole **9**, in 15% yield. While the yields were low, the generation of these novel N–S-PAC structures, which are highly interesting from the viewpoint of optoelectronic properties yet otherwise difficult to synthesize by conventional organic reactions, is notable.

**Fig. 2:**
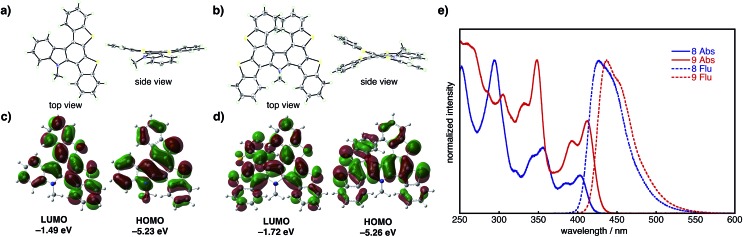
Top and side views of the X-ray crystal structures of (a) **8** and (b) **9**. Thermal ellipsoids are drawn at 50% probability. Pictorial Frontier molecular orbitals and energy levels of (c) **8** and (d) **9** calculated using the B3LYP/6-31G(d) level of theory. (e) Normalized UV-vis absorption and fluorescence spectra of **8** and **9** in CH_2_Cl_2_ at rt.

The structural and electronic properties of **8** and **9** were elucidated *via* X-ray crystallography, UV-vis/photoluminescence spectroscopy, and DFT/TD-DFT calculations at the B3LYP/6-31G(d) level of theory (Fig. 2). Single crystal X-ray structures (Fig. 2a, b, S2 and S3†) reveal that compound **8** adopts a relatively flattened structure in the solid state (Fig. 2a), while compound **9** possesses a twisted structure owing to the embedded heterohelicene moiety. DFT calculations for **8** (Fig. 2c) reveal delocalization of the HOMO (–5.23 eV) over the entire molecule, while the LUMO (–1.49 eV) localizes on a benzothienocarbazole wing. On the other hand, the HOMO and LUMO of **9** are delocalized over entire molecule, and thus the energy level of LUMO (–1.72 eV) is slightly lower than that of **8**. The UV-vis absorption spectra of **8** and **9** in CH_2_Cl_2_ show that both compounds have broad absorption bands between 300 and 450 nm (Fig. 2e). Absorption maxima were found at 294, 317, 339, 357, 381 and 399 nm in **8**, and the corresponding peaks were also found in **9** at 305, 332, 348, 393 and 412 nm. The TD-DFT calculations revealed that the longest-wavenumber absorptions in **8** and **9** (399 and 412 nm) are attributed to the allowed HOMO–LUMO transitions (see ESI† for details). The fluorescence spectra of **8** and **9** in CH_2_Cl_2_ display broad emissions with emission maxima of 427 and 437 nm, respectively (Fig. 2e).

## Conclusions

In summary, we have developed a novel palladium-catalyzed APEX reaction to enable the annulative π-extension of indoles/pyrroles with diiodobiaryls. Use of the Pd(OPiv)_2_/Ag_2_CO_3_ catalytic system in a mixed DMF/DMSO solvent allows the preparation of a diverse range of N-PACs in a single step, including several previously unsynthesized structures. Rapid access to exotic scaffolds such as complex, unsymmetrically substituted tetrabenzocarbazoles and extended *N*-heteroarenes featuring multiple helicene moieties is a particular highlight of the present APEX protocol. Developed APEX methodology also has great potential for the efficient and rapid synthesis of planar and non-planar π-extended N-PACs such as π-extended azacorannulenes, aza-buckybowls and pyrrolopyrroles which are regarded as one of promising materials for optoelectronics.^22^ Further investigations into the reaction mechanism and applications of this APEX method towards the synthesis of larger π-extended heteroaromatics are currently underway.

## Conflicts of interest

There are no conflicts to declare.

## Supplementary Material

Supplementary information

Crystal structure data

## References

[cit1] Knölker H.-J., Reddy K. R. (2002). Chem. Rev..

[cit2] Chen Z., Wang B., Zhang J., Yu W., Liu Z., Zhang Y. (2015). Org. Chem. Front..

[cit3] Yamaguchi J., Yamaguchi A. D., Itami K. (2012). Angew. Chem., Int. Ed..

[cit4] (a) Eicher T., Hauptmann S. and Speicher A., Five-Membered Heterocycles, in The Chemistry of Heterocycles: Structure, Reactions, Syntheses, and Applications, Wiley-VCH Verlag GmbH & Co. KGaA, Weinheim, 2nd edn, 2003, ch. 5.1–5.21, pp. 52–121.

[cit5] For a review on APEX reactions, see: Ito H. Ozaki K. Itami K., Angew. Chem., Int. Ed., 2017, 56 , 11144 –11164 . 10.1002/anie.201701058 28370962

[cit6] Ozaki K., Kawasumi K., Shibata M., Ito H., Itami K. (2015). Nat. Commun..

[cit7] Ozaki K., Zhang H., Ito H., Lei A., Itami K. (2013). Chem. Sci..

[cit8] Trost B. M. (1991). Science.

[cit9] Yamashita M., Horiguchi H., Hirano K., Satoh T., Miura M. (2009). J. Org. Chem..

[cit10] Guo T., Jiang Q., Huang F., Chen J., Yu Z. (2014). Org. Chem. Front..

[cit11] Thies N., Hrib C. G., Haak E. (2012). Chem.–Eur. J..

[cit12] Dawande S. G., Kanchupalli V., Kalepu J., Chennamsetti H., Lad B. S., Katukojvala S. (2014). Angew. Chem., Int. Ed..

[cit13] Paria S., Reiser O. (2014). Adv. Synth. Catal..

[cit14] Wu Y., Peng X., Luo B., Wu F., Liu B., Song F., Huang P., Wen S. (2014). Org. Biomol. Chem..

[cit15] Qin C., Lu W. (2008). J. Org. Chem..

[cit16] Lebrasseur N., Larrosa I. (2008). J. Am. Chem. Soc..

[cit17] (b) Nova A. Ujaque G. Maseras F. Lledós A. Espinet P., J. Am. Chem. Soc., 2006, 128 , 14571 –14578 , . Also see ref. 6a and 16 . 17090041 10.1021/ja0635736

[cit18] Li C.-W., Wang C.-I., Liao H.-Y., Chaudhuri R., Liu R.-S. (2007). J. Org. Chem..

[cit19] Changing the reaction temperature or the amount of diiodobiphenyl did not change this result (see ESI for details)

[cit20] (b) Hyun S. Y., Jung S. O. and Oh H. J., KR Pat. 20150121626A, 2015.

[cit21] In this reaction, the previously developed APEX conditions using Pd(CH_3_CN)_4_(BF_4_)_2_/AgOPiv/TfOH gave the best result in terms of yield of **6**. On the other hand, the present APEX conditions did not give **6**

[cit22] Ito S., Tokimaru Y., Nozaki K. (2015). Angew. Chem., Int. Ed..

