# Supplementary material for: Annulative π-extension of indoles and pyrroles with diiodobiaryls by Pd catalysis: rapid synthesis of nitrogen-containing polycyclic aromatic compounds
Source: Chem Sci. 2018 Aug 9;9(38):7556–61. doi: 10.1039/c8sc02802h (PMC6180308; doi:10.1039/c8sc02802h)

Electronic Supplementary Material (ESI) for Chemical Science.
This journal is © The Royal Society of Chemistry 2018

Supporting Information

Annulative π-Extension of Indoles and Pyrroles with Diiodobiaryls by Pd Catalysis: Rapid Synthesis of Nitrogen-Containing Polycyclic Aromatic Compounds

Hiroyuki Kitano,[#] Wataru Matsuoka,[†] Hideto Ito,*[,†,‡] and Kenichiro Itami*[,#,†,‡]

[#]*Institute of Transformative Bio-Molecules (WPI-ITbM), Nagoya University, Chikusa, Nagoya 464-8602, Japan*

[†]*Graduate School of Science, Nagoya University, Chikusa, Nagoya 464-8602, Japan*

[‡]*JST-ERATO, Itami Molecular Nanocarbon Project, Nagoya University, Chikusa, Nagoya 464-8602, Japan*

E-mail: itami@chem.nagoya-u.ac.jp (K.I.), ito.hideto@g.mbox.nagoya-u.ac.jp (H.I.)

Table of Contents

1. General	S2
2. Palladium-catalyzed APEX reaction of indoles and pyrroles with diiodobiaryls	S3–14
3. Experiments for mechanistic considerations	S15
4. Effect of reaction parameters	S16–18
5. X-ray crystallographic analysis of **3ad**, **8** and **9**	S19–21
6. DFT calculations of **8** and **9**	S22–25
7. Absorption and emission spectra of **8** and **9**	S26
8. ^{1}H and ^{13}C NMR spectra	S27–50

1. General

Unless otherwise noted, all reactants or reagents including dry solvents were obtained from commercial suppliers and used as received. $Pd(OPiv)_2$ was obtained from Aldrich. Ag_2CO_3 was purchased from WAKO. 4,4′-Dibromo-2,2′-diiodo-1,1′-biphenyl (**2c**) was purchased from TCI. 2,2′-Diiodo-1,1′-biphenyl (**2a**)[1], 4-chloro-2,2′-diiodo-1,1′-biphenyl (**2b**)[2], 2,2′-diiodo-1,1′-binaphthalene (**2d**)[3], 3,3′-diiodo-2,2′-bibenzothiophene (**7**)[4], 2,2′-dibromo-1,1′-biphenyl (**2e**)[1], 1,2-dimethyl-1*H*-indole (**10**)[5], and 1,3-dimethyl-1*H*-indole (**11**)[5] were synthesized according to procedures reported in the literature. Unless otherwise noted, all reactions were performed with dry solvents under air. All work-up and purification procedures were carried out with reagent-grade solvents in air.

Analytical thin-layer chromatography (TLC) was performed using E. Merck silica gel 60 F_{254} precoated plates (0.25 mm). The developed chromatogram was analyzed by UV lamp (254 nm). Medium pressure liquid chromatography (MPLC) was performed using Yamazen W-prep 2XY. Preparative thin-layer chromatography (PTLC) was performed using Wakogel B5-F silica coated plates (0.75 mm) prepared in our laboratory. Preparative gel permeation chromatography (GPC) was performed with a JAI LC-9204 instrument equipped with JAIGEL-1H/JAIGEL-2H columns using chloroform as an eluent. The high-resolution mass spectra (HRMS) were conducted on Thermo Fisher Scientific Exactive. Nuclear magnetic resonance (NMR) spectra were recorded on a JEOL JNM-ECA-600 (^{1}H 600 MHz, ^{13}C 150 MHz) spectrometer and a JEOL JNM-ECA-600II with Ultra COOL™ probe (^{1}H 600 MHz, ^{13}C 150 MHz) spectrometer. Chemical shifts for ^{1}H NMR are expressed in parts per million (ppm) relative to tetramethylsilane (δ 0.00 ppm). Chemical shifts for ^{13}C NMR are expressed in ppm relative to $CDCl_3$ (δ 77.0 ppm). Data are reported as follows: chemical shift, multiplicity (s = singlet, d = doublet, dd = doublet of doublets, ddd = doublet of doublets of doublets, t = triplet, dt = doublet of triplets, td = triplet of doublets, q = quartet, m = multiplet), coupling constant (Hz), and integration.

1 G. Shi, D. Chen, H. Jiang, Y. Zhang and Y. Zhang, *Org. Lett.*, 2016, **18**, 2958-2961.
2 B. Wu and N. Yoshikai, *Angew. Chem. Int. Ed.*, 2015, **54**, 8736-8739.
3 X. Li, J.-W. Han and H. N. C. Wong, *Asian J. Org. Chem.*, 2016, **5**, 74-81.
4 M. Miura, T. Sato, H. Tsurugi, A. Kumagai and M. Ueda, Jpn Pat., 2009190999, 2009.
5 X.-H. Xu, G.-K. Liu, A. Azuma, E. Tokunaga and N. Shibata, *Org. Lett.*, 2011, **13**, 4854-4857.

2. Palladium-catalyzed APEX reaction of indoles and pyrroles with diiodobiaryls

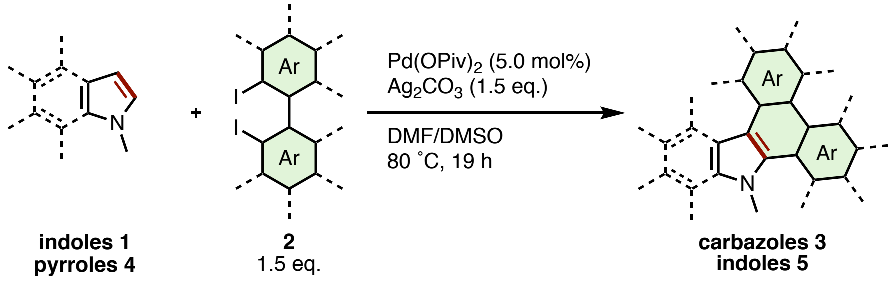

General procedure A: To a screw-capped glass tube containing a magnetic stirrer bar were added indole **1** or pyrrole **4** (0.20 mmol, 1.0 equiv), diiodobiaryl **2** (0.30 mmol, 1.5 equiv), Ag_2CO_3 (0.30 mmol, 1.5 equiv), $Pd(OPiv)_2$ (0.010 mmol, 5.0 mol%), DMF (0.7 mL) and DMSO (0.3 mL) under air. After stirring at 80 °C for 19 h, the reaction mixture was cooled to room temperature, and then passed through a short pad of Celite® (eluent: EtOAc or $CHCl_3$). After the organic solvent was removed under reduced pressure, the residue was purified by PTLC or MPLC to yield the corresponding π-extended carbazole **3** or indole **5**.

General procedure B: To a screw-capped glass tube containing a magnetic stirrer bar were added indole **1** or pyrrole **4** (0.10 mmol, 1.0 equiv), diiodobiaryl **2** (0.15 mmol, 1.5 equiv), Ag_2CO_3 (0.15 mmol, 1.5 equiv), $Pd(OPiv)_2$ (0.0050 mmol, 5.0 mol%), DMF (0.35 mL) and DMSO (0.15 mL) under air. After stirring at 80 °C for 19 h, the reaction mixture was cooled to room temperature, and then passed through a short pad of Celite® (eluent: EtOAc or $CHCl_3$). After the organic solvent was removed under reduced pressure, the residue was purified by PTLC or MPLC to yield the corresponding π-extended carbazole **3** or indole **5**.

9-Methyl-9*H*-dibenzo[*a*,*c*]carbazole (3aa)

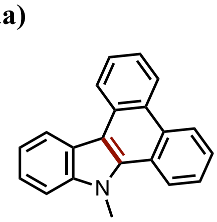

3aa

General procedure A: Purification by MPLC (hexane/$CHCl_3$ = hexane only to 9:1) afforded **3aa** as a white solid (37 mg, 66% yield). ^1H NMR (600 MHz, $CDCl_3$) δ 8.86–8.81 (m, 2H), 8.74 (d, *J* = 8.4 Hz, 1H), 8.68–8.64 (m, 1H), 8.59 (d, *J* = 8.4 Hz, 1H), 7.74 (ddd, *J* =7.8, 6.9, 0.9 Hz, 1H), 7.67–7.62 (m, 2H), 7.59–7.54 (m, 2H), 7.49 (ddd, *J* =8.4, 7.2, 1.2 Hz, 1H), 7.39 (ddd, *J* = 8.4, 7.2, 1.2 Hz, 1H), 4.35 (s. 3H). ^{13}C NMR (150 MHz, $CDCl_3$) δ 140.8, 134.7. 130.9, 129.9, 127.3, 126.9, 126.1, 125.6,

124.1, 123.9, 123.7, 123.60, 123.56, 123.4, 122.9, 121.8, 120.3, 113.4, 109.5, 34.6 (one carbon peak was not observed because of overlapping). HRMS (ESI$^+$) *m*/*z* calcd for $C_{21}H_{16}N$ $[M+H]^+$: 282.1277, found: 282.1277.

9-Ethyl-9*H*-dibenzo[*a*,*c*]carbazole (3ba)

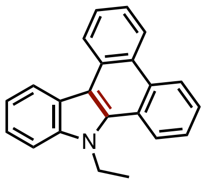

3ba

General procedure A: Purification by MPLC (hexane/$CHCl_3$ = hexane only to 9:1) afforded **3ba** as a white solid (42 mg, 71% yield). ^{1}H NMR (600 MHz, $CDCl_3$) δ 8.87 (t, *J* = 9.0 Hz, 2H), 8.76 (d, *J* = 8.4 Hz, 1H), 8.63 (d, *J* = 8.4 Hz, 1H), 8.53 (dd, *J* = 8.1, 1.5 Hz, 1H), 7.74 (ddd, *J* = 8.1, 7.2, 1.2 Hz, 1H), 7.71–7.65 (m, 2H), 7.62 (d, *J* = 8.4 Hz, 1H), 7.57 (ddd, *J* = 8.1, 7.5, 1.2 Hz, 1H), 7.50 (ddd, *J* = 8.1, 7.2, 1.2 Hz, 1H), 7.41 (ddd, *J* = 8.1, 6.9, 1.2 Hz, 1H), 4.85 (q, *J* = 7.2 Hz, 2H), 1.71 (t, *J* = 7.2 Hz, 3H). ^{13}C NMR (150 MHz, $CDCl_3$) δ 140.1, 133.7, 130.9, 130.0, 127.3, 126.9, 126.5, 125.6, 124.2, 123.8, 123.63, 123.56, 123.4, 122.6, 122.0, 120.4, 113.6, 109.4, 41.0, 15.3 (two carbon peaks were not observed because of overlapping). HRMS (ESI$^+$) *m*/*z* calcd for $C_{22}H_{18}N$ $[M+H]^+$: 296.1434, found: 296.1433.

9-Benzyl-9*H*-dibenzo[*a*,*c*]carbazole (3ca)

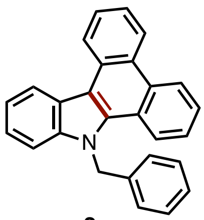

3ca

General procedure A: Purification by MPLC (hexane/$CHCl_3$ = hexane only to 9:1) afforded **3ca** as a white solid (50 mg, 70% yield). ^{1}H NMR (600 MHz, $CDCl_3$) δ 8.92 (d, *J* = 8.4 Hz, 1H), 8.82 (d, *J* = 8.4 Hz, 1H), 8.76 (d, *J* = 8.4 Hz, 1H), 8.68–8.64 (m, 1H), 8.23 (dd, *J* = 8.1, 0.9 Hz, 1H), 7.77 (ddd, *J* = 8.1, 6.9, 1.2 Hz, 1H), 7.59 (ddd, *J* = 8.4, 7.2, 1.2 Hz, 2H), 7.48–7.42 (m, 4H), 7.37–7.33 (m, 2H), 7.32–7.27 (m, 3H), 5.95 (s, 2H). ^{13}C NMR (150 MHz, $CDCl_3$) δ 141.3, 137.4, 134.7, 130.9, 129.9, 129.1, 127.5, 127.4, 127.1, 126.4, 126.0, 125.7, 124.1, 124.0, 123.8, 123.7, 123.5, 123.2, 122.9, 121.9, 120.9, 113.9, 110.0, 50.2 (one carbon peak was not observed because of overlapping). HRMS (ESI$^+$) *m*/*z* calcd for $C_{27}H_{20}N$ $[M+H]^+$: 358.1590, found: 358.1591.

9-Phenyl-9*H*-dibenzo[*a*,*c*]carbazole (3da)

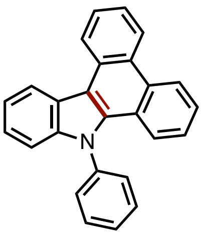

3da

General procedure A: Purification by MPLC (hexane/$CHCl_3$ = hexane only to 7:3) afforded **3da** as a white solid (32 mg, 47% yield). ^1H NMR (600 MHz, $CDCl_3$) δ 8.94 (d, *J* = 8.4 Hz, 1H), 8.80 (dd, *J* = 8.4, 4.8 Hz, 2H), 8.66 (d, *J* = 8.4 Hz, 1H), 7.79 (ddd, *J* = 8.1, 6.9, 0.9 Hz, 1H), 7.68–7.60 (m, 4H), 7.58–7.51 (m, 3H), 7.47 (dd, *J* = 8.4, 0.6 Hz, 1H), 7.43 (ddd, *J* = 8.1, 7.2, 1.2 Hz, 1H), 7.38 (ddd, *J* = 8.4, 7.2, 1.2 Hz, 1H), 7.27 (ddd, *J* = 8.1, 7.8, 0.6 Hz, 1H), 7.21 (d, *J* = 8.4 Hz, 1H). ^{13}C NMR (150 MHz, $CDCl_3$) δ 142.1, 140.2, 134.5, 130.8, 130.2, 129.9, 129.1, 128.9, 127.4, 127.3, 125.9, 125.7, 124.0, 123.9, 123.81, 123.79, 123.5, 123.2, 121.7, 121.0, 114.2, 111.0 (two carbon peaks were not observed because of overlapping). HRMS (ESI^+) *m*/*z* calcd for $C_{26}H_{18}N$ $[M+H]^+$: 344.1434, found: 344.1433.

5,6-Dihydro-4*H*-dibenzo[*a*,*c*]pyrido[3,2,1-*jk*]carbazole (3ea)

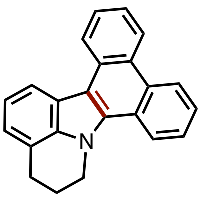

3ea

General procedure A: Purification by MPLC (hexane/$CHCl_3$ = hexane only to 1:1) afforded **3ea** as a pale yellow solid (44 mg, 72% yield). ^1H NMR (600 MHz, $CDCl_3$) δ 8.89–8.83 (m, 2H), 8.78 (d, *J* = 8.4 Hz, 1H), 8.69–8.66 (m, 1H), 8.40 (d, *J* = 7.8 Hz, 1H), 7.75 (ddd, *J* = 8.1, 7.2, 1.2 Hz, 1H), 7.70–7.66 (m, 2H), 7.58 (ddd, *J* = 8.1, 6.9, 1.2 Hz, 1H) 7.31 (t, *J* = 7.5 Hz, 1H), 7.20 (dd, *J* = 6.9, 0.9 Hz, 1H), 4.99 (t, *J* = 6.0 Hz, 2H), 3.17 (t, *J* = 6.0 Hz, 2H), 2.42 (quintet, *J* = 6.0 Hz, 2H). ^{13}C NMR (150 MHz, $CDCl_3$) δ 136.8, 133.6, 130.7, 130.4, 127.3, 126.6, 126.1, 125.5, 124.1, 123.7, 123.43, 123.38, 122.8, 122.4, 121.6, 121.0, 120.2, 119.4, 113.3, 46.7, 25.1, 23.7 (one carbon peak was not observed because of overlapping). HRMS (ESI^+) *m*/*z* calcd for $C_{23}H_{18}N$ $[M+H]^+$: 308.1434, found: 308.1432.

9-Benzyl-12-nitro-9*H*-dibenzo[*a*,*c*]carbazole (3ga)

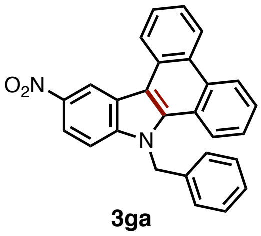

3ga

General procedure A: After purification by MPLC ($CHCl_3$ only), the obtained solid was washed with hexane and CH_2Cl_2 to afford **3ga** as a yellow solid (36 mg, 45% yield). ^{1}H NMR (600 MHz, $CDCl_3$) δ 9.62 (d, J = 1.8 Hz, 1H), 8.92 (d, J = 8.4 Hz, 1H), 8.88 (d, J = 8.4 Hz, 1H), 8.81 (d, J = 8.4 Hz, 1H), 8.36 (dd, J = 8.7, 2.1 Hz, 1H), 8.27 (d, J = 7.2 Hz, 1H), 7.87 (ddd, J = 8.1, 6.9, 1.5 Hz, 1H), 7.71–7.67 (m, 2H), 7.55–7.51 (m, 2H), 7.42–7.38 (m, 2H), 7.37–7.33 (m, 1H), 7.28 (d, J = 8.4 Hz, 1H), 6.08 (s, 2H). ^{13}C NMR (150 MHz, $CDCl_3$) δ 143.9, 142.2, 136.7, 136.0, 131.6, 129.4, 128.8, 128.0, 127.6, 126.9, 126.8, 125.8, 125.0, 124.3, 123.7, 123.5, 123.1, 122.8, 122.5, 119.7, 118.7, 114.7, 109.7, 50.5 (one carbon peak was not observed because of overlapping). HRMS (ESI$^+$) *m*/*z* calcd for $C_{27}H_{18}N_2O_2Na$ [M+Na]$^+$: 425.1260, found: 425.1260.

9-Benzyl-9*H*-dibenzo[*a*,*c*]carbazole-12-carbonitrile (3ha)

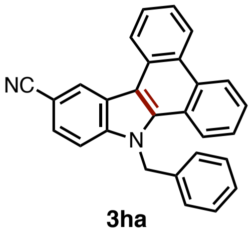

3ha

General procedure A: After purifcation by MPLC ($CHCl_3$ only), the obtained solid was washed with hexane to afford **3ha** as a pale yellow solid (34 mg, 44% yield). ^{1}H NMR (600 MHz, $CDCl_3$) δ 9.01 (d, J = 1.2 Hz, 1H), 8.88 (d, J = 8.4 Hz, 1H), 8.82 (dd, J = 8.1, 4.5 Hz, 2H), 8.27 (d, J = 8.4 Hz, 1H), 8.84 (ddd, J = 8.4, 7.2, 1.2 Hz, 1H), 7.71–7.66 (m, 3H), 7.56–7.51 (m, 2H) 7.41–7.37 (m, 2H), 7.36–7.33 (m, 1H), 7.30–7.26 (m, 2H), 6.06 (s, 2H). ^{13}C NMR (150 MHz, $CDCl_3$) δ 142.5, 136.2, 135.8, 131.4, 129.3, 128.9, 127.92, 127.86, 127.4, 126.9, 126.8, 126.6, 125.7, 124.8, 124.1, 123.64, 123.60, 123.4, 122.8, 122.5, 120.6, 113.5, 110.6, 103.8, 50.2 (one carbon peak was not observed because of overlapping). HRMS (ESI$^+$) *m*/*z* calcd for $C_{28}H_{19}N_2$ [M+H]$^+$: 383.1543, found: 383.1547.

9-Benzyl-12-bromo-9H-dibenzo[a,c]carbazole (3ia)

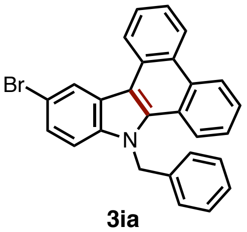

3ia

General procedure A: After purification by MPLC (hexane/$CHCl_3$ = hexane only to 1:1), the obtained solid was washed with hexane to afford **3ia** as a pale yellow solid (54 mg, 62% yield). ^1H NMR (600 MHz, $CDCl_3$) δ 8.85 (d, J = 8.4 Hz, 1H), 8.81–8.77 (m, 3H), 8.25 (d, J = 8.4 Hz, 1H), 7.80 (ddd, J = 8.1, 7.2, 1.2 Hz, 1H), 7.64 (ddd, J = 9.6, 7.2, 1.2 Hz, 2H), 7.53 (dd, J = 9.0, 1.8 Hz, 1H), 7.49 (ddd, J = 8.4, 7.2, 1.2 Hz, 1H), 7.39–7.35 (m, 3H), 7.34–7.30 (m, 1H), 7.28 (d, J = 7.2 Hz, 2H), 5.99 (s, 2H). ^{13}C NMR (150 MHz, $CDCl_3$) δ 139.9, 136.9, 135.3, 131.2, 129.5, 129.2, 127.73, 127.66, 127.2, 126.8, 126.6, 126.2, 125.9, 125.4, 124.5, 124.3, 124.1, 123.6, 123.5, 123.0, 122.9, 114.0, 113.2, 111.4, 50.3. HRMS (ESI^+) m/z calcd for $C_{27}H_{19}BrN$ $[M+H]^+$: 436.0695, found: 436.0696.

9-Benzyl-12-methoxy-9H-dibenzo[a,c]carbazole (3ja)

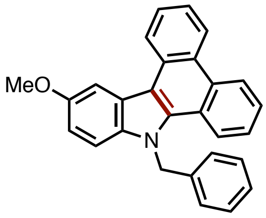

3ja

General procedure A: After purification by MPLC (hexane/$CHCl_3$ = 7:3 to 1:1), the obtained solid was washed with hexane to afford **3ja** as a white solid (38 mg, 49% yield). ^1H NMR (600 MHz, $CDCl_3$) δ 8.85 (dd, J = 7.8, 3.0 Hz, 2H), 8.79 (d, J = 7.8 Hz, 1H), 8.25 (d, J = 7.8 Hz, 1H), 8.13 (d, J = 2.4 Hz, 1H), 7.79 (ddd, J = 8.1, 6.9, 1.2 Hz, 1H), 7.64–7.59 (m, 2H), 7.48 (ddd, J = 8.4, 7.2, 1.2 Hz, 1H), 7.42–7.35 (m, 3H), 7.33–7.28 (m, 3H), 7.12 (dd, J = 8.4, 2.4 Hz, 1H), 5.99 (s, 2H), 4.05 (s, 3H). ^{13}C NMR (150 MHz, $CDCl_3$) δ 154.9, 137.5, 136.5, 135.2, 130.9, 130.0, 129.1, 127.5, 127.4, 127.0, 126.4, 126.0, 125.7, 124.1, 124.0, 123.7, 123.5, 123.3, 122.8, 113.5, 113.2, 110.5, 105.0, 56.2, 50.3 (one carbon peak was not observed because of overlapping). HRMS (ESI^+) m/z calcd for $C_{28}H_{22}NO$ $[M+H]^+$: 388.1696, found: 388.1700.

9-Benzyl-11-methoxy-9*H*-dibenzo[*a*,*c*]carbazole (3ka)

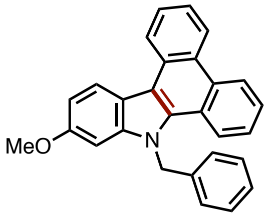

3ka

General procedure A: After purification by MPLC (hexane/$CHCl_3$ = 7:3 to 1:1), the obtained solid was washed with hexane to afford **3ka** as a yellow solid (41 mg, 52% yield). ^{1}H NMR (600 MHz, $CDCl_3$) δ 8.87 (d, *J* = 7.8 Hz, 1H), 8.85 (d, *J* = 7.8 Hz, 1H), 8.79 (d, *J* = 8.4 Hz, 1H), 8.55 (d, *J* = 9.0 Hz, 1H), 8.24 (d, *J* = 8.4 Hz, 1H), 7.77 (ddd, *J* = 8.4, 7.2, 1.2 Hz, 1H), 7.62–7.57 (m, 2H), 7.47 (ddd, *J* = 8.4, 7.2, 1.2 Hz, 1H), 7.40–7.31 (m, 5H), 7.08 (dd, *J* = 8.7, 2.1 Hz, 1H), 6.95 (d, *J* = 2.4 Hz, 1H), 5.97 (s, 2H), 3.90 (s, 3H). ^{13}C NMR (150 MHz, $CDCl_3$) δ 157.9, 142.7, 137.4, 134.2, 130.3, 129.6, 129.2, 127.5, 127.3, 127.1, 126.4, 126.0, 125.2, 124.0, 123.8, 123.54, 123.49, 123.3, 122.7, 122.4, 118.1, 114.2, 109.7, 94.0, 55.7, 50.4. HRMS (ESI$^+$) *m*/*z* calcd for $C_{28}H_{22}NO$ [M+H]$^+$: 388.1696, found: 388.1696.

9-Benzyl-9*H*-dibenzo[*a*,*c*]carbazole-10-carbonitrile (3la)

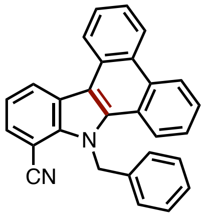

3la

General procedure A: After purification by MPLC ($CHCl_3$ only), the obtained solid was washed with hexane to afford **3la** as a pale yellow solid (32 mg, 42% yield). ^{1}H NMR (600 MHz, $CDCl_3$) δ 8.86 (t, *J* = 9.0 Hz, 2H), 8.89 (d, *J* = 7.8 Hz, 1H), 8.73 (d, *J* = 8.4 Hz, 1H), 8.28 (d, *J* = 8.4 Hz, 1H), 7.83–7.79 (m, 2H), 7.72–7.64 (m, 3H), 7.54 (ddd, *J* = 8.4, 7.2, 1.2 Hz, 1H), 7.42–7.34 (m, 3H), 7.29 (d, *J* = 7.2 Hz, 2H), 6.04 (s, 2H). ^{13}C NMR (150 MHz, $CDCl_3$) δ 139.9, 137.0, 136.2, 131.7, 129.4, 129.2, 128.0, 127.8, 127.4, 127.0, 126.8, 126.7, 125.7, 124.7, 124.2, 123.70, 123.67, 123.4, 123.2, 122.5, 122.3, 120.2, 114.2, 113.5, 106.3, 50.2. HRMS (ESI$^+$) *m*/*z* calcd for $C_{28}H_{19}N_2$ [M+H]$^+$: 383.1543, found: 383.1546.

9-Benzyl-10-(benzyloxy)-9*H*-dibenzo[*a*,*c*]carbazole (3ma)

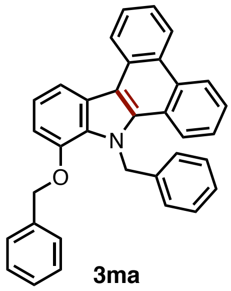

3ma

General procedure A: After purification by MPLC (hexane/$CHCl_3$ = 7:3 to 1:1), the obtained solid was washed with hexane/CH_2Cl_2 solution to afford **3ma** as a pale yellow solid (37 mg, 40% yield). ^{1}H NMR (600 MHz, $CDCl_3$) δ 8.93 (d, J = 8.4 Hz, 1H), 8.84 (d, J = 7.8 Hz, 1H), 8.78 (d, J = 9.0 Hz, 1H), 8.32 (d, J = 8.4 Hz, 1H), 8.26 (d, J = 9.0 Hz, 1H), 7.77 (ddd, J = 8.4, 6.9, 1.5 Hz, 1H), 7.62–7.58 (m, 2H), 7.44–7.40 (m, 1H), 7.35–7.21 (m, 7H), 7.19 (d, J = 6.6 Hz, 2H), 7.03 (d, J = 7.2 Hz, 2H), 7.00 (d, J = 7.8 Hz, 1H), 6.30 (br s, 2H), 5.06 (s, 2H). ^{13}C NMR (150 MHz, $CDCl_3$) δ 146.9, 139.8, 136.4, 135.6, 131.0, 130.9, 129.8, 128.7, 128.4, 127.7, 127.6, 127.3, 126.7, 126.33, 126.28, 125.9, 125.6, 124.0, 123.8, 123.6, 123.4, 123.32, 123.26, 121.2, 114.9, 114.2, 106.6, 70.7, 52.3 (one carbon peak was not observed because of overlapping). HRMS (ESI$^+$) *m/z* calcd for $C_{34}H_{26}NO$ $[M+H]^+$: 464.2009, found: 464.2011.

1-Ethyl-1*H*-dibenzo[*e*,*g*]indole (5aa)

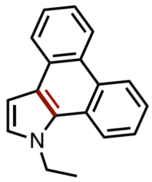

5aa

General procedure A: Purification by MPLC (hexane/$CHCl_3$ = hexane only to 7:3) and GPC afforded **5aa** as a white solid (19 mg, 39% yield). ^{1}H NMR (600 MHz, $CDCl_3$) δ 8.78 (dt, J = 7.8, 0.6 Hz, 1H), 8.65 (d, J = 8.4 Hz, 1H), 8.29 (dd, J = 9.0, 1.2 Hz, 1H), 8.22 (d, J = 8.1, 0.9 Hz, 1H), 7.64–7.57 (m, 2H), 7.54 (ddd, J = 8.1, 6.9, 1.2 Hz, 1H), 7.50 (ddd, J = 8.4, 6.9, 1.2 Hz, 1H), 7.11 (d, J = 2.4 Hz, 1H), 7.02 (d, J = 3.0 Hz, 1H), 4.61 (q, J = 7.2 Hz, 2H), 1.60 (t, J = 7.5 Hz, 3H). ^{13}C NMR (150 MHz, $CDCl_3$) δ 128.9, 128.8, 127.1, 126.8, 126.7, 126.4, 124.5, 124.2, 124.0, 123.8, 123.2, 123.1, 121.0, 100.9, 45.4, 16.4 (two carbon peaks were not observed because of overlapping). HRMS (ESI$^+$) *m/z* calcd for $C_{18}H_{16}N$ $[M+H]^+$: 246.1277, found: 246.1278.

1-Benzyl-1*H*-dibenzo[*e*,*g*]indole (5ba)

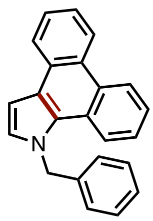

5ba

General procedure A: Purification by MPLC (hexane/$CHCl_3$ = hexane only to 4:1) and PTLC (hexane/$CHCl_3$ = 7:3) afforded **5ba** as a white solid (26 mg, 41% yield). ^{1}H NMR (600 MHz, $CDCl_3$) δ 8.72 (d, J = 8.4 Hz, 1H), 8.64 (d, J = 9.0 Hz, 1H), 8.26 (d, J = 8.4 Hz, 1H), 8.05 (d, J = 8.4 Hz, 1H), 7.64–7.60 (m, 1H), 7.54–7.50 (m, 1H), 7.47–7.44 (m, 1H), 7.41–7.37 (m, 1H), 7.30–7.26 (m, 2H), 7.25–7.21 (m, 1H), 7.16 (d, J = 3.6 Hz, 1H), 7.13 (dd, J = 9.3, 0.9 Hz, 1H), 7.09 (s, 1H), 7.08 (s, 1H), 5.81 (s, 2H). ^{13}C NMR (150 MHz, $CDCl_3$) δ 137.7, 129.0, 128.9, 128.71, 128.67, 127.6, 127.5, 127.0, 126.8, 126.3, 125.9, 124.2, 124.1, 124.0, 123.9, 123.3, 123.14, 123.11, 121.3, 101.5, 53.9. HRMS (ESI$^+$) *m*/*z* calcd for $C_{23}H_{18}N$ [M+H]$^+$: 308.1434, found: 308.1433.

1:1.4 Mixture of 2-Chloro-9-methyl-9*H*-dibenzo[*a*,*c*]carbazole (3ab) and 7-Chloro-9-methyl-9*H*-dibenzo[*a*,*c*]carbazole (3ab′)

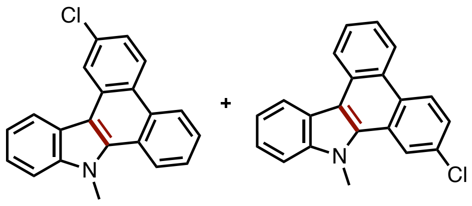

3ab **3ab′**

General procedure B: Purification by MPLC (hexane/$CHCl_3$ = hexane only to 1:1) afforded a regioisomeric mixture of **3ab** and **3ab′**as a yellow powder (30 mg, 94% yield, **3ab/3ab′** = 1:1.4). ^{1}H NMR (600 MHz, $CDCl_3$) δ 8.75 (d, J = 8.4 Hz, 1.4×1H), 8.67–8.61 (m, 2H + 1.4×1H), 8.60–8.50 (m, 2H + 1.4×2H), 8.47 (d, J = 2.4 Hz, 1.4×1H), 8.44 (d, J = 8.4 Hz, 1H), 7.71 (ddd, J = 8.1, 6.6, 1.2 Hz, 1.4×1H), 7.63–7.59 (m, 2H), 7.55–7.47 (m, 2H + 1.4×4H), 7.44 (dd, J = 8.7, 2.1 Hz, 1H), 7.41–7.36 (m, 1H + 1.4×1H), 4.25 (s, 3H), 4.21 (s, 1.4×3H). ^{13}C NMR (100 MHz, $CDCl_3$) δ 140.7, 140.6, 135.0, 133.22, 133.18, 132.0, 130.8, 130.3, 129.7, 129.0, 127.5, 126.30, 126.25, 125.8, 125.7, 125.5, 125.0, 124.9, 124.6, 124.1, 123.92, 123.89, 123.8, 123.7, 123.61, 123.58, 123.3, 123.1, 123.0, 122.9, 122.7, 122.1, 121.9, 121.5, 120.5, 120.4, 114.2, 112.3, 109.6 (2C), 34.4, 34.2. HRMS (ESI$^+$) *m*/*z* calcd for $C_{21}H_{15}ClN$ [M+H]$^+$: 316.0888, found: 316.0892.

2,7-Dibromo-9-methyl-9*H*-dibenzo[*a*,*c*]carbazole (3ac)

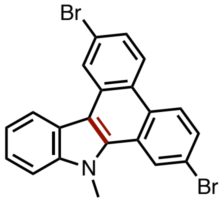

3ac

General procedure B: Purification by MPLC (hexane/$CHCl_3$ = hexane only to 1:1) afforded **3ac** as a white solid (36 mg, 81% yield). ^{1}H NMR (600 MHz, $CDCl_3$) δ 8.68 (d, *J* = 1.8 Hz, 1H), 8.54 (d, *J* = 2.4 Hz, 1H), 8.42 (d, *J* = 9.0 Hz, 1H), 8.34 (d, *J* = 8.4 Hz, 1H), 8.33 (d, *J* = 9.0 Hz, 1H), 7.65 (dd, *J* = 8.7, 2.1 Hz, 1H), 7.55 (dd, *J* = 9.0, 1.8 Hz, 1H), 7.54–7.52 (m, 2H), 7.42–7.38 (m, 1H), 4.13 (s, 3H). ^{13}C NMR (150 MHz, $CDCl_3$) δ 140.6, 133.4, 130.9, 128.72, 128.65, 126.7, 125.9, 125.4, 125.2, 124.8, 124.7, 124.4, 122.6, 121.9, 121.6, 120.8, 120.5, 112.9, 109.6, 34.2 (one carbon peak was not observed because of overlapping). HRMS (APCI$^+$) *m*/*z* calcd for $C_{21}H_{14}Br_2N$ $[M+H]^+$: 439.9467, found: 439.9468.

5,10-Dibromo-1-methyl-1*H*-dibenzo[*e*,*g*]indole (5cc)

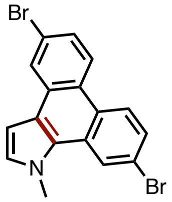

5cc

General procedure A: Purification by MPLC (hexane/$CHCl_3$ = 7:3 to 1:1) and GPC afforded **5cc** as a white solid (35 mg, 44% yield). ^{1}H NMR (600 MHz, $CDCl_3$) δ 8.56–8.53 (m, 2H), 8.43 (d, *J* = 8.4 Hz, 1H), 8.32 (d, *J* = 1.8 Hz, 1H), 7.64 (dd, *J* = 9.0, 1.8 Hz, 1H), 7.59 (dd, *J* = 8.7, 2.1 Hz, 1H), 7.10 (d, *J* = 3.0 Hz, 1H), 6.96 (d, *J* = 2.4 Hz, 1H), 4.30 (s, 3H). ^{13}C NMR (150 MHz, $CDCl_3$) δ 130.1, 129.5, 127.3, 127.1, 127.0, 126.9, 125.9, 125.7, 125.6, 124.94, 124.88, 123.5, 122.5, 121.3, 120.8, 100.8, 38.8. HRMS (ESI$^+$) *m*/*z* calcd for $C_{17}H_{12}Br_2N$ $[M+H]^+$: 389.9311, found: 389.9312.

11-Methyl-11*H*-dinaphtho[2,1-*a*:1′,2′-*c*]carbazole (3ad)

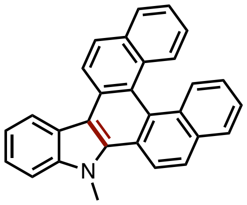

3ad

General procedure B: Purification by MPLC (hexane/$CHCl_3$ = hexane only to 3:2) afforded **3ad** as a yellow solid (9.7 mg, 25% yield). A single crystal of **3ad** was obtained by recrystallization from a

$CHCl_3$ solution through vapor diffusion of pentane at room temperature, and the structure was determined by X-ray crystallography (see Table S5 and Figure S1). ^{1}H NMR (600 MHz, $CDCl_3$) δ 8.95 (d, *J* = 9.0 Hz, 1H), 8.79 (d, *J* = 9.0 Hz, 1H), 8.68 (d, *J* = 8.4 Hz, 1H), 8.32 (d, *J* = 8.4 Hz, 1H), 8.21 (d, *J* = 9.0 Hz, 1H), 8.12 (d, *J* = 9.0 Hz, 1H), 8.03 (d, *J* = 9.0 Hz, 1H), 7.97 (d, *J* = 7.8 Hz, 2H), 7.64 (d, *J* = 8.4 Hz, 1H), 7.54 (ddd, *J* = 8.1, 7.2, 1.2 Hz, 1H), 7.50 (ddd, *J* = 8.1, 6.9, 1.2 Hz, 1H), 7.45–7.41 (m, 2H), 7.26–7.19 (m, 2H), 4.48 (s, 3H). ^{13}C NMR (150 MHz, $CDCl_3$) δ 141.8, 135.9, 131.8, 131.6, 131.1, 130.5, 130.4, 129.2, 129.1, 128.3, 127.8, 127.4, 126.9, 126.8, 125.9, 124.8, 124.7, 124.5, 124.2, 123.2, 122.1, 121.9, 121.5, 120.4, 120.2, 115.4, 109.6, 34.7 (one carbon peak was not observed because of overlapping). HRMS (ESI$^+$) *m*/*z* calcd for $C_{29}H_{20}N$ $[M+H]^+$: 382.1590, found: 382.1592.

1-Methyl-1*H*-dibenzo[*e*,*g*]indole (5ca)

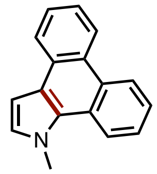

5ca

To a screw-capped glass tube containing a magnetic stirrer bar were added 1-methylpyrrole (**4c**) (2.0 mmol, 1.0 equiv), 2,2′-diiodo-1,1′-biphenyl (**2a**) (3.0 mmol, 1.5 equiv), Ag_2CO_3 (3.0 mmol, 1.5 equiv), $Pd(OPiv)_2$ (0.10 mmol, 5.0 mol%), DMF (7.0 mL) and DMSO (3.0 mL) under air. After stirring at 80 °C for 19 h, the reaction mixture was cooled to room temperature, and then passed through a short pad of Celite® (eluent: $CHCl_3$). After the organic solvent was removed under reduced pressure, the residue was purified by MPLC (hexane/$CHCl_3$ = hexane only to 7:3) to afford **5ca** as a white solid (171 mg, 37% yield). ^{1}H NMR (600 MHz, $CDCl_3$) δ 8.77 (dt, *J* = 8.4, 0.6 Hz, 1H), 8.65 (dd, *J* = 7.8, 0.6 Hz, 1H), 8.47 (dd, *J* = 8.4, 0.6 Hz, 1H), 8.22 (dd, *J* = 8.1, 0.9 Hz, 1H), 7.62–7.58 (m, 2H), 7.55 (ddd, *J* = 8.4, 7.2, 1.2 Hz, 1H), 7.51 (ddd, *J* = 8.4, 6.9, 1.5 Hz, 1H), 7.05 (d, *J* = 3.0 Hz, 1H), 7.00 (d, *J* = 2.4 Hz, 1H), 4.30 (s, 3H). ^{13}C NMR (150 MHz, $CDCl_3$) δ 128.9, 128.7, 128.6, 127.9, 126.83, 126.76, 126.3, 124.9, 124.1, 124.0, 123.9, 123.3, 123.1, 122.7, 121.0, 100.6, 38.9. HRMS (ESI$^+$) *m*/*z* calcd for $C_{17}H_{14}N$ $[M+H]^+$: 232.1121, found: 232.1123.

2,7-Dibromo-9-methyl-9*H*-tetrabenzo[*a*,*c*,*g*,*i*]carbazole (6)

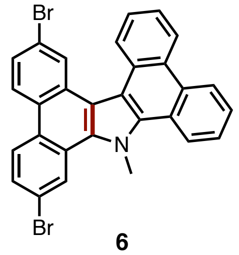

6

To a screw-capped glass tube containing a magnetic stirrer bar were added *N*-methyldibenzoindole (**5ca**) (0.20 mmol, 1.0 equiv), 4,4′-dibromo-2,2′-diiodo-1,1′-biphenyl (**2a**) (0.30 mmol, 1.5 equiv), AgOPiv (0.40 mmol, 2.0 equiv), $Pd(CH_3CN)_2(BF_4)_2$ (0.010 mmol, 5.0 mol%), 1,2-dichloroethane (2.0 mL) and TfOH (0.40 mmol, 2.0 equiv) under air. After stirring at 50 °C for 12 h, the reaction mixture was cooled to room temperature, and then passed through a short pad of Celite® (eluent: $CHCl_3$). After the organic solvent was removed under reduced pressure, the residue was purified by MPLC (hexane/$CHCl_3$ = hexane only to 7:3), and then the obtained solid was washed with hexane to afford **6** as a white solid (36 mg, 33% yield). ^{1}H NMR (600 MHz, $CDCl_3$) δ 9.13 (d, *J* = 1.8 Hz, 1H), 8.87 (dd, *J* = 8.1, 1.5 Hz, 1H), 8.84 (dd, *J* = 8.1, 1.5 Hz, 1H), 8.76 (dd, *J* = 8.1, 1.5 Hz, 1H), 8.61 (d, *J* = 1.8 Hz, 1H), 8.59 (d, *J* = 9.0 Hz, 1H), 8.51–8.48 (m, 2H), 7.74–7.62 (m, 6H), 4.59 (s, 3H). ^{13}C NMR (150 MHz, $CDCl_3$) δ 137.9, 136.5, 131.0, 130.1, 128.8, 128.34, 128.3, 128.13, 128.08, 127.6, 126.4, 126.2, 126.0, 125.8, 125.6, 125.33, 125.28, 125.2, 124.9, 124.1, 123.9, 123.3, 123.0, 120.7, 120.3, 117.1, 116.8, 41.2 (one carbon peak was not observed because of overlapping). HRMS (APCI$^+$) *m*/*z* calcd for $C_{29}H_{18}Br_2N$ $[M+H]^+$: 539.9780, found: 539.9781.

11-Methyl-11*H*-benzo[4,5]thieno[3,2-*a*]benzo[4,5]thieno[2,3-*c*]carbazole (8)

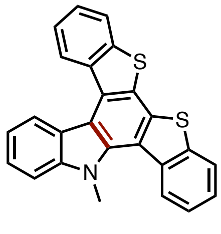

8

General procedure B: Purification by MPLC (hexane/$CHCl_3$ = hexane only to 4:1) and GPC afforded **8** as a yellow solid (13 mg, 32% yield). A single crystal of **8** was obtained by recrystallization from a $CHCl_3$ solution through vapor diffusion of pentane at room temperature, and the structure was determined by X-ray crystallography (see Table S5 and Figure S2). ^{1}H NMR (600 MHz, $CDCl_3$) δ 9.16 (d, *J* = 8.4 Hz, 1H), 8.92 (d, *J* = 8.4 Hz, 1H), 8.49 (d, *J* = 7.8 Hz, 1H), 8.01 (dd, *J* = 7.5, 5.1 Hz, 2H), 7.68 (d, *J* = 8.4 Hz, 1H), 7.65–7.52 (m, 4H), 7.50 (ddd, *J* = 8.4, 7.2, 1.2 Hz, 1H), 7.45 (ddd, *J* = 8.4, 6.9, 1.2 Hz, 1H), 4.23 (s, 3H). ^{13}C NMR (150 MHz, $CDCl_3$) δ 144.3, 139.8, 139.3, 138.8, 136.3, 134.7, 133.1, 129.8, 126.8, 126.1, 125.5, 125.4, 125.3, 125.2, 124.4, 124.2, 123.9, 123.22, 123.15,

122.8, 120.24, 120.21, 117.8, 111.0, 37.4. HRMS (ESI$^+$) *m*/*z* calcd for $C_{25}H_{16}NS_2$ [M+H]$^+$: 394.0719, found: 394.0720.

21-Methyl-21*H*-benzo[4,5]thieno[3,2-*a*]benzo[4,5]thieno[2,3-*c*]benzo[4,5]thieno[3,2-*g*]benzo[4,5]thieno[2,3-*i*]carbazole (9)

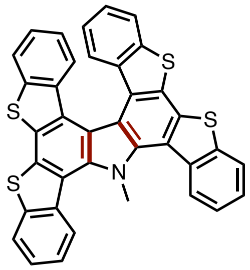

9

To a screw-capped glass tube containing a magnetic stirrer bar were added *N*-methylpyrrole (**4c**) (0.20 mmol, 1.0 equiv), 2,2′-diiodo-1,1′-biaryl (**7**) (0.60 mmol, 3.0 equiv), Ag_2CO_3 (0.60 mmol, 3.0 equiv), $Pd(OPiv)_2$ (0.010 mmol, 5.0 mol%), DMF (0.7 mL) and DMSO (0.3 mL) under air. After stirring at 80 °C for 19 h, the reaction mixture was cooled to room temperature, and then passed through a short pad of Celite® (eluent: $CHCl_3$). After the organic solvent was removed under reduced pressure, the residue was purified by MPLC (hexane/$CHCl_3$ = 19:1 to 3:2) and GPC to afford **9** as a yellow solid (18 mg, 15% yield). A single crystal of **9** was obtained by recrystallization from a THF solution through vapor diffusion of pentane at room temperature, and the structure was determined by X-ray crystallography. ^{1}H NMR (600 MHz, $CDCl_3$) δ 8.83 (d, *J* = 7.8 Hz, 2H), 8.09 (d, *J* = 7.8 Hz, 2H), 7.95 (d, *J* = 7.8 Hz, 2H), 7.80 (ddd, *J* = 8.4, 7.2, 1.2 Hz, 2H), 7.69 (d, *J* = 8.4 Hz, 2H), 7.62 (ddd, *J* = 8.1, 7.2, 1.2 Hz, 2H), 7.29 (ddd, *J* = 8.1, 7.2, 0.9 Hz, 2H), 6.78 (ddd, *J* = 8.1, 7.2, 0.9 Hz, 2H), 3.98 (s, 3H). ^{13}C NMR (150 MHz, $CDCl_3$) δ 144.7, 139.7, 139.2, 136.6, 134.4, 133.0, 129.2, 129.1, 127.7, 126.1, 125.3, 125.1, 123.2, 122.7, 122.3, 122.2, 120.0, 41.4 (one carbon peak was not observed because of overlapping). HRMS (APCI$^+$) *m*/*z* calcd for $C_{37}H_{20}NS_4$ [M+H]$^+$: 606.0473, found: 606.0473.

3. Experiments for mechanistic considerations

Palladium-catalyzed phenylation of 1,2-dimethylindole (10)

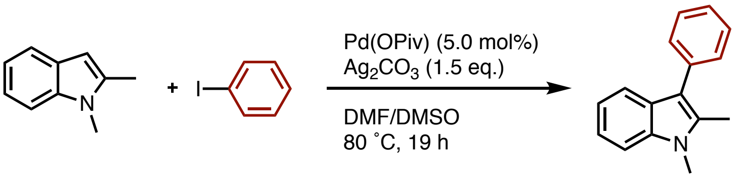

To a screw-capped glass tube containing a magnetic stirrer bar were added 1,2-dimethylindole (**10**) (0.20 mmol, 1.0 equiv), iodobenzene (0.60 mmol, 3.0 equiv), Ag_2CO_3 (0.30 mmol, 1.5 equiv), $Pd(OPiv)_2$ (0.010 mmol, 5.0 mol%), DMF (0.7 mL) and DMSO (0.3 mL) under air. After stirring at 80 °C for 19 h, the reaction mixture was cooled to room temperature, and then passed through a short pad of Celite® (eluent: EtOAc or $CHCl_3$). After the organic solvent was removed under reduced pressure, the residue was purified by PTLC (hexane/AcOEt = 10:1) to afford 1,2-dimethyl-3-phenyl-1*H*-indole (**12**) as a white solid (18 mg, 40% yield). ^1H NMR (600 MHz, $CDCl_3$) δ 7.66 (d, J = 7.8 Hz, 1H), 7.51–7.43 (m, 4H), 7.33–7.27 (m, 2H), 7.21 (ddd, J = 8.1, 7.2, 0.9 Hz, 1H), 7.11 (ddd, J = 8.1, 7.2, 1.2 Hz, 1H), 3.74 (s, 3H), 2.49 (s, 3H). ^{13}C NMR (150 MHz, $CDCl_3$) δ 136.6, 135.8, 133.3, 129.7, 128.4, 126.9, 125.6, 121.1, 119.6, 118.7, 114.0, 108.7, 29.6, 11.1. HRMS (ESI$^+$) *m*/*z* calcd for $C_{16}H_{16}N$ $[M+H]^+$: 222.1277, found: 222.1278.

Palladium-catalyzed C–H phenylation of 1,3-dimethylindole (11)

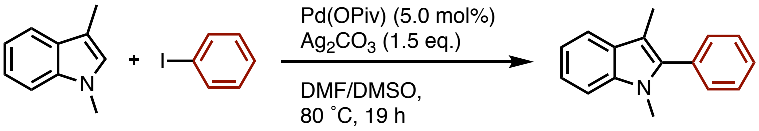

To a screw-capped glass tube containing a magnetic stirrer bar were added 1,3-dimethylindole (**11**) (0.20 mmol, 1.0 equiv), iodobenzene (0.60 mmol, 3.0 equiv), Ag_2CO_3 (0.30 mmol, 1.5 equiv), $Pd(OPiv)_2$ (0.010 mmol, 5.0 mol%), DMF (0.7 mL) and DMSO (0.3 mL) under air. After stirring at 80 °C for 19 h, the reaction mixture was cooled to room temperature, and then passed through a short pad of Celite® (eluent: EtOAc or $CHCl_3$). After the organic solvent was removed under reduced pressure, the residue was purified by PTLC (hexane/AcOEt = 10:1) to afford 1,3-dimethyl-2-phenyl-1*H*-indole (**13**) as a white solid (36 mg, 80% yield). ^1H NMR (600 MHz, $CDCl_3$) δ 7.60 (dt, J = 7.8, 1.2 Hz, 1H), 7.50–7.46 (m, 2H), 7.42–7.38 (m, 3H), 7.33 (d, J = 7.8 Hz, 1H), 7.25 (ddd, J = 8.4, 7.2, 1.2 Hz, 1H), 7.15 (ddd, J = 8.1, 7.2, 1.2 Hz, 1H), 3.61 (s, 3H), 2.29 (s, 3H). ^{13}C NMR (150 MHz, $CDCl_3$) δ 137.6, 137.2, 132.1, 130.6, 128.4, 128.3, 127.7, 121.7, 119.1, 118.8, 109.2, 108.5, 30.9, 9.3. HRMS (ESI$^+$) *m*/*z* calcd for $C_{16}H_{16}N$ $[M+H]^+$: 222.1277, found: 222.1278.

4. Effect of reaction parameters

Table S1. Effect of solvent.

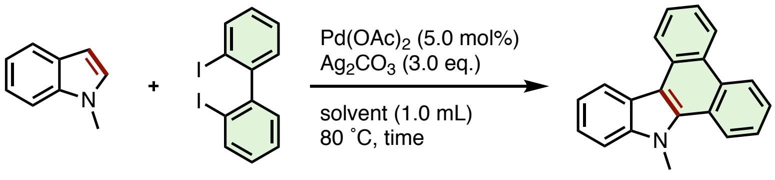

1a
0.2 mmol

2a
1.5 eq.

3aa

entry	solvent	time (h)	NMR yield[a]
1	DMAc	16	10%
2	DMF	16	10%
3	DMSO	16	29%
4	CH_3CN	16	15%
5	1,2-dichloroethane	16	trace
6	TFE	16	trace
7	HFIP	16	trace
8	1,4-dioxane	16	trace
9	toluene	16	trace
10	DMAc/DMSO (7:3)	21	47%
11	DMF/DMSO (7:3)	21	54%
12	1,2-dichloroethane/DMSO (7:3)	21	39%
13	1,4-dioxane/DMSO (7:3)	21	51%
14	CH_3CN/DMSO (7:3)	19	30%
15	DMF (1.0 mL), DMSO (1.0 eq.)	21	20%
16	DMF/DMSO (9:1)	19	36%
17	DMF/DMSO (8:2)	19	41%
18	DMF/DMSO (5:5)	19	45%
19	DMF/DMSO (2:8)	19	39%

[a]Determined by ^{1}H NMR using CH_2Br_2 as an internal standard.

Table S2. Effect of base.

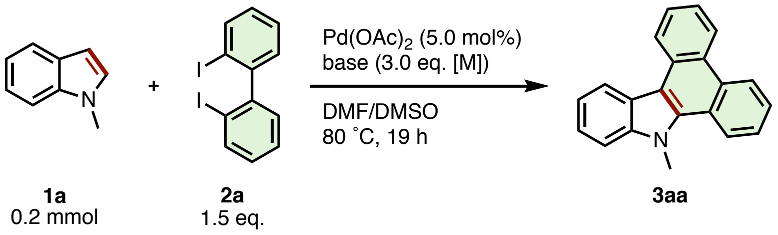

entry	base	NMR yield[a] (isolated yield)
1	AgOAc	33%
2	AgOPiv	0%
3	AgOTs	0%
4	AgOTFA	0%
5	Ag_2O	32%
6	Ag_2CO_3	78% (66%)
7	Na_2CO_3	0%
8	K_2CO_3	0%
9	Cs_2CO_3	0%

[a]Determined by ^{1}H NMR using CH_2Br_2 as an internal standard.

Table S3. Effect of ligand.

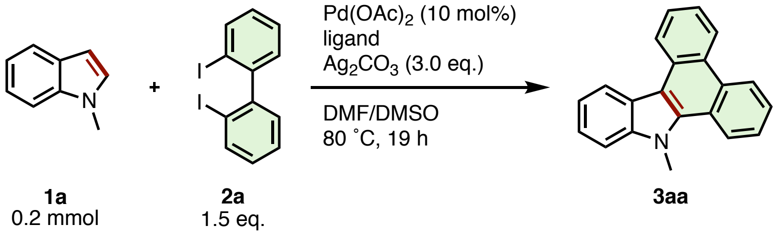

entry	ligand	NMR yield[a]
1	none	43%
2	2,2′-bipyridyl (10 mol%)	trace
3	1,10-phenanthroline (10 mol%)	4%
4	rac-BINAP (10 mol%)	43%
5	PPh_3 (20 mol%)	40%
6	Xanthphos (10 mol%)	29%
7	BrettPhos (20 mol%)	40%
8	SPhos (20 mol%)	46%
9	DavePhos (20 mol%)	36%
10	**L1** (10 mol%)	23%
11	**L2** (10 mol%)	41%
12	**L3** (10 mol%)	21%
13	**L4** (10 mol%)	37%
14	**L5** (10 mol%)	53%
15	**L6** (10 mol%)	44%
16	**L7** (10 mol%)	18%
17[b]	**L5** (5 mol%)	61%

[a]Determined by ^{1}H NMR using CH_2Br_2 as an internal standard.

[b]$Pd(OPiv)_2$ (5 mol%) was used

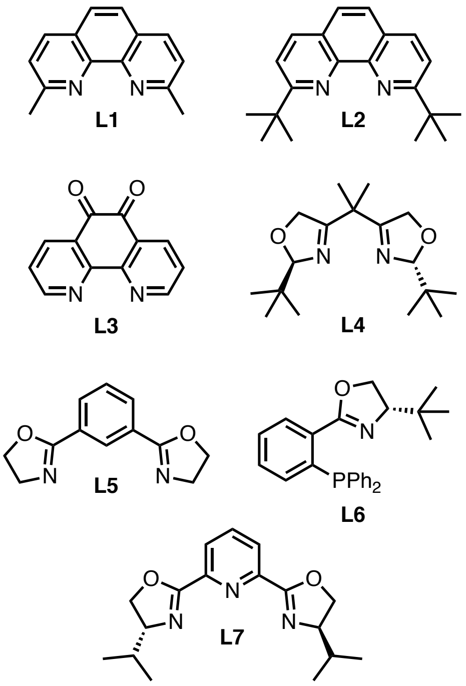

Scheme S1. APEX reaction of **1a** by using dibromobiphenyl (**2e**) as a π-extending agent.

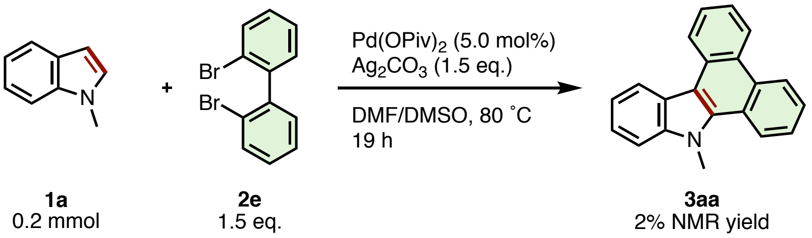

Table S4. Effect of temperature in APEX reaction of pyrrole **4a**.

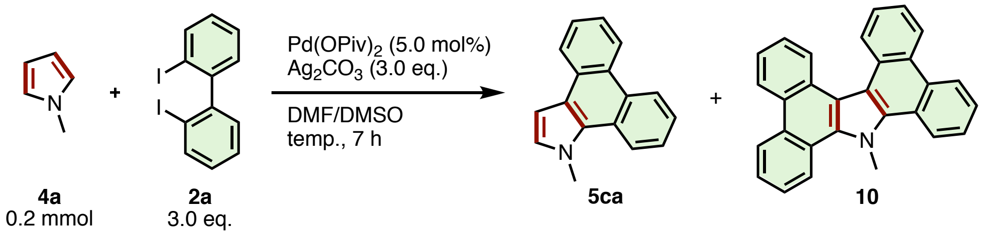

entry	temp. (°C)	NMR yield[a] (**5ca** / **10**)
1	80	38% / trace
2	90	39% / trace
3	100	18% / trace

[a]Determined by ^{1}H NMR using CH_2Br_2 as an internal standard.

5. X-ray crystallographic analysis of 3ad, 8 and 9

Each single crystal of **3ad**, **8** and **9** was obtained by the recrystallizations from $CHCl_3$/pentane, $CHCl_3$/pentane and THF/pentane, respectively. Details of the crystal data and a summary of the intensity data collection parameters for **3ad**, **8**, and **9** are listed in Table S1. A suitable crystal, obtained by crystallization from appropriate solution, was mounted with mineral oil on a MiTeGen MicroMounts and transferred to the goniometer of a Rigaku PILATUS diffractometer. Graphite-monochromated Mo Kα radiation (λ = 0.71075 Å) was used. The structures were solved by direct methods with (SIR-97)[6] and refined by full-matrix least-squares techniques against F^2 (SHELXL-2014/7)[7] by using Yadokari-XG software package.[8] The intensities were corrected for Lorentz and polarization effects. The non-hydrogen atoms were refined anisotropically. Hydrogen atoms were placed using AFIX instructions.

Table S5. Crystallographic data and structure refinement detail for **3ad**, **8** and **9**

	**3ad**	**8**	**9**
CCDC deposition No.	1848311	1848309	1848310
Formula	$C_{29}H_{19}N$	$C_{25}H_{15}NS_2$	$C_{41}H_{27}NOS_4$
Fw	381.45	393.50	677.87
T (K)	123(2)	123(2)	123(2)
λ (Å)	0.71073	0.71073	0.71073
cryst syst	Monoclinic	Orthorhombic	Monoclinic
space group	$P2_1/n$	Pbca	$P2_1/c$
a, (Å)	10.6434(15)	18.7793(2)	16.1846(6)
b, (Å)	9.1028(13)	7.45210(10)	27.2713(9)
c, (Å)	20.109(3)	50.3987(6)	7.6092(3)
α, (deg)	90	90	90
β, (deg)	102.565(15)	90	97.890(3)
γ, (deg)	90	90	90
V, (Å^3)	1901.6(5)	7053.06(15)	3326.7(2)
Z	4	16	4
D_{calc}, (g / cm^3)	1.332	1.482	1.353
μ (mm^{-1})	0.077	0.313	0.321
F(000)	800	3264	1408
cryst size (mm)	0.20 × 0.05 × 0.05	0.25 × 0.10 × 0.10	0.20 × 0.10 × 0.02
θ range, (deg)	2.009–24.998	1.946–24.999	1.961–24.999
reflns collected	13195	68569	30329
indep reflns/R_{int}	3357/0.1713	6212/0.0227	5832/0.0434
params	272	507	469
GOF on F^2	1.010	1.278	1.086
R_1, wR_2 [I>2σ(I)]	0.1013, 0.2480	0.0410, 0.0917	0.0653, 0.2172
R_1, wR_2 (all data)	0.1869, 0.3280	0.0419, 0.0921	0.0871, 0.2336

[6] A. Altomare, M. C. Burla, M. Camalli, G. L. Cascarano, C. Giacovazzo, A. Guagliardi, A. G. G. Moliterni, G. Polidori and R. Spagna, *J. Appl. Crystallogr.*, 1999, **32**, 115.
[7] G. M. Sheldrick, *Acta Crystallogr. A*, 2008, **64**, 112-122.
[8] a) K. Wakita, Yadokari-XG, Software for crystal structure analyses, 2001; b) C. Kabuto, S. Akine, T. Nemoto and E. Kwon, *J. Cryst. Soc. Jpn.*, 2009, **51**, 218-224.

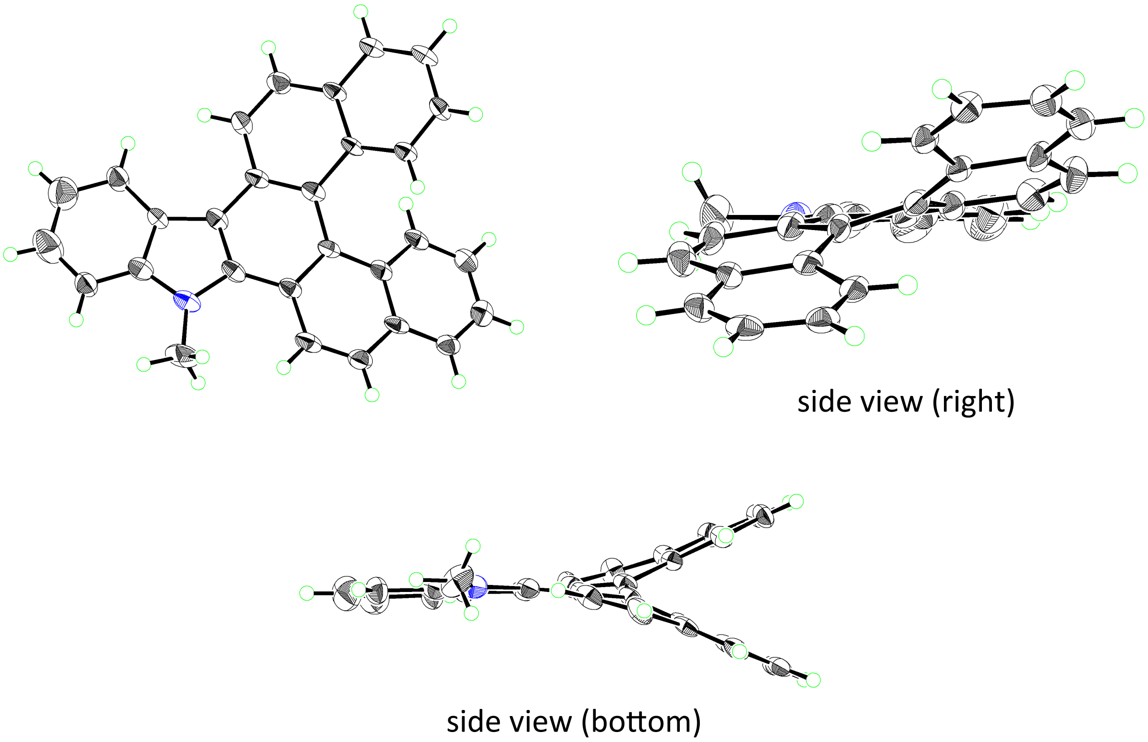

Figure S1. ORTEP drawings of **3ad** with 50% thermal ellipsoid.

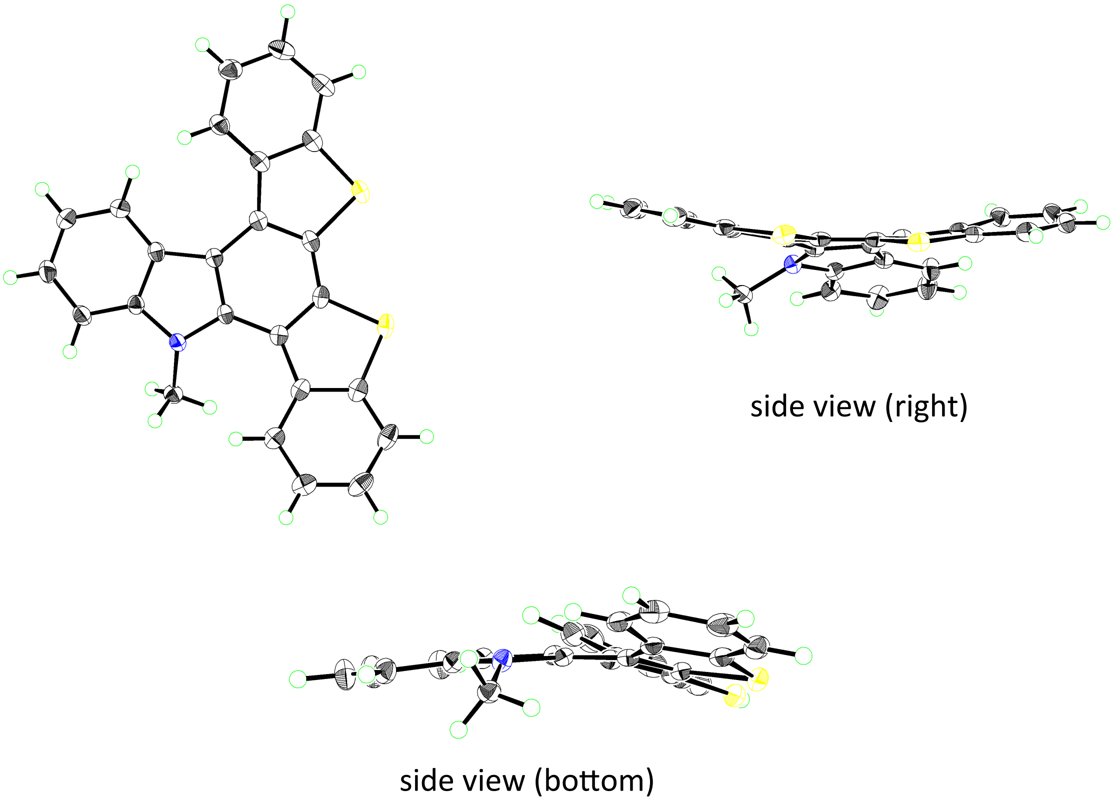

Figure S2. ORTEP drawings of **8** with 50% thermal ellipsoid.

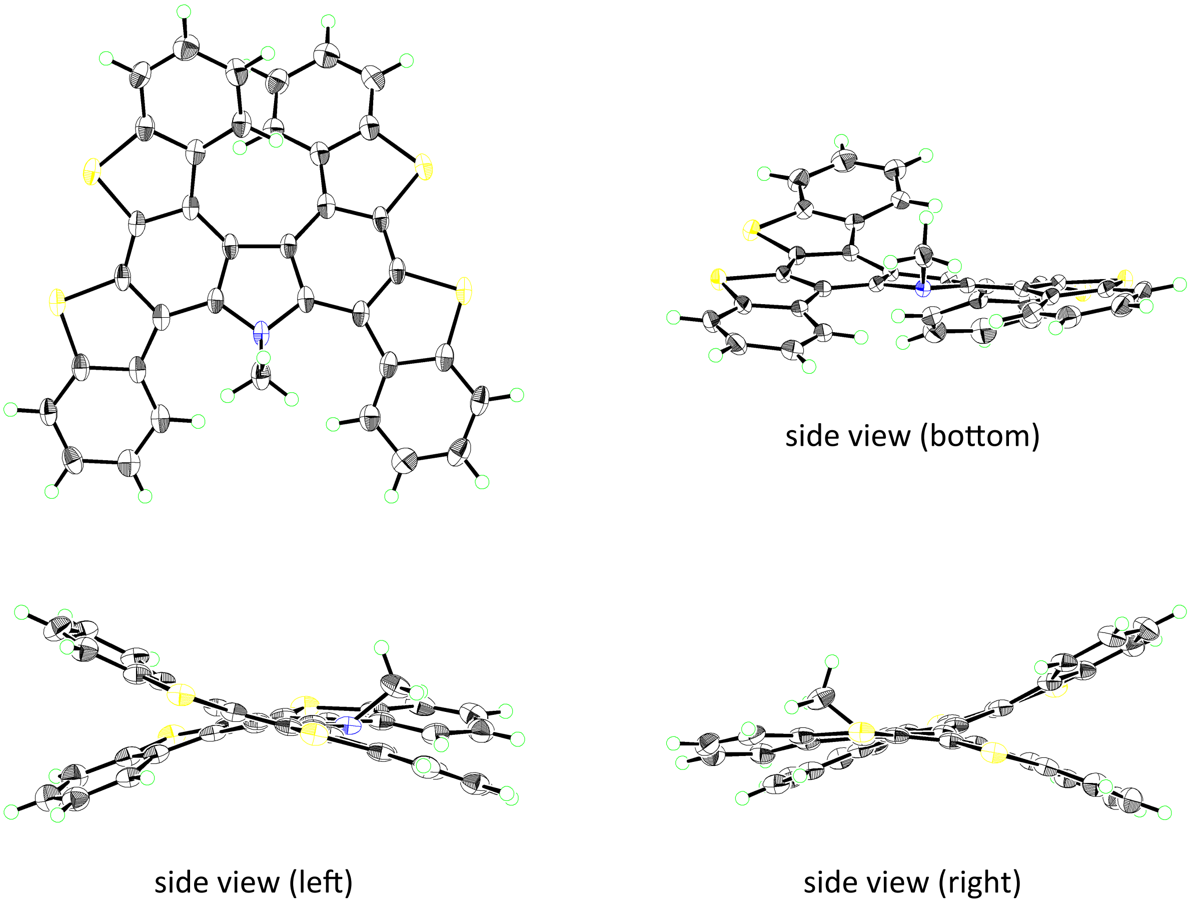

Figure S3. ORTEP drawings of **9** with 50% thermal ellipsoid.

6. DFT calculations of 8 and 9

The Gaussian 09 program[9] running on a SGI Altix4700 system was used for geometry optimization (B3LYP/6-31G(d)).[10] All structures were optimized without any symmetry assumptions. Zero-point energy, enthalpy, and Gibbs free energy at 298.15 K and 1 atm were estimated from the gas-phase unless otherwise noted. Calculations of harmonic vibration frequencies, molecular orbitals and their energies at the same level were performed to verify all stationary points as local minima (with no imaginary frequency) or transition states (with one imaginary frequency). Visualization of the results was performed by use of GaussView 5.0 software.

Table S6. Uncorrected and thermal-corrected (298 K) energies of stationary points (Hartree).[a]

structure	E	$E + ZPE$	H	G
**8**	-1812.87556461	-1812.548257	-1812.526842	-1812.596592
**9**	-3068.96648317	-3068.518491	-3068.486765	-3068.578818

a) E: electronic energy; ZPE: zero-point energy; H (= $E + ZPE + E_{\mathrm{vib}} + E_{\mathrm{rot}} + E_{\mathrm{trans}} + RT$): sum of electronic and thermal enthalpies; G (= $H - TS$): sum of electronic and thermal free energies.

Cartesian coordinates of optimized structures

9 M. J. Frisch, G. W. Trucks, H. B. Schlegel, G. E. Scuseria, M. A. Robb, J. R. Cheeseman, G. Scalmani, V. Barone, B. Mennucci, G. A. Petersson, H. Nakatsuji, M. Caricato, X. Li, H. P. Hratchian, A. F. Izmaylov, J. Bloino, G. Zheng, J. L. Sonnenberg, M. Hada, M. Ehara, K. Toyota, R. Fukuda, J. Hasegawa, M. Ishida, T. Nakajima, Y. Honda, O. Kitao, H. Nakai, T. Vreven, J. A. Montgomery, Jr., J. E. Peralta, F. Ogliaro, M. Bearpark, J. J. Heyd, E. Brothers, K. N. Kudin, V. N. Staroverov, T. Keith, R. Kobayashi, J. Normand, K. Raghavachari, A. Rendell, J. C. Burant, S. S. Iyengar, J. Tomasi, M. Cossi, N. Rega, J. M. Millam, M. Klene, J. E. Knox, J. B. Cross, V. Bakken, C. Adamo, J. Jaramillo, R. Gomperts, R. E. Stratmann, O. Yazyev, A. J. Austin, R. Cammi, C. Pomelli, J. W. Ochterski, R. L. Martin, K. Morokuma, V. G. Zakrzewski, G. A. Voth, P. Salvador, J. J. Dannenberg, S. Dapprich, A. D. Daniels, O. Farkas, J. B. Foresman, J. V. Ortiz, J. Cioslowski and D. J. Fox, Gaussian 09, Revision D.01, Gaussian, Inc., Wallingford CT, 2013.

10 a) A. D. Becke, *J. Chem. Phys.*, 1993, **98**, 5648-5652; b) C. Lee, W. Yang and R. G. Parr, *Phys. Rev. B*, 1988, **37**, 785-789.

Compound **8**

C	0.30925000	3.07541200	-0.03921000
C	1.38308400	2.14870300	-0.01702900
C	-0.62860900	1.02499200	-0.02130600
C	0.78038800	0.82391700	0.01443300
C	1.29834600	-0.50406700	-0.01707800
C	2.64489300	-1.03108400	0.18441300
C	0.39110800	-1.54644200	-0.29883100
C	2.72615800	-2.41759700	-0.10076600
C	3.79662000	-0.40943600	0.70175900
C	3.91844000	-3.13451600	0.01369800
C	4.98360900	-1.12109700	0.83387700
H	3.75047600	0.61477400	1.04575700
C	5.05470100	-2.47295200	0.46851400
H	3.95058700	-4.19350500	-0.22578600
H	5.86102100	-0.62462000	1.23858200
H	5.99021200	-3.01602300	0.56865900
C	-1.54565100	-0.05914900	-0.04256100
C	-2.98636400	-0.14607600	0.18512200
C	-0.99168000	-1.33055600	-0.30137500
C	-3.48404300	-1.44918400	-0.06790500
C	-3.88788200	0.80156700	0.70634500
C	-4.83259300	-1.77479600	0.08382700
C	-5.22965500	0.47807200	0.87607000
H	-3.53438800	1.77717700	1.01541700
C	-5.70907600	-0.79685100	0.54432900
H	-5.18497900	-2.77968400	-0.13017000
H	-5.90973300	1.22093300	1.28331500
H	-6.76099200	-1.03429900	0.67465800
N	-0.90352500	2.39448100	-0.05035300
S	1.16439300	-3.11595600	-0.51354300
S	-2.20876200	-2.59016300	-0.48529900
C	-2.07644800	3.01688700	-0.65645400
H	-2.65129200	3.60277400	0.06996100
H	-1.75399700	3.68550400	-1.46180900
H	-2.72317100	2.25308800	-1.08438100
C	0.50644000	4.45683900	-0.11642800
H	-0.33349900	5.14483800	-0.11644500
C	1.81370900	4.92696700	-0.19301500
H	1.99644000	5.99663300	-0.24600700
C	2.89326300	4.03092600	-0.22190400
H	3.90594200	4.41233900	-0.31732700
C	2.68765100	2.65668200	-0.14411300
H	3.53801700	1.99364200	-0.22695600

Compound **9**

C	1.19279800	-0.93227300	-0.22681400
C	0.68985400	0.37957000	-0.04310700
C	-1.04833700	-1.11128300	-0.09180600
C	-0.76794300	0.27152000	-0.10768200
C	-1.85720600	1.17594600	-0.29888000
C	-1.88991900	2.54534700	-0.80334400
C	-3.16747400	0.66746100	-0.14498800
C	-3.20604800	3.06613900	-0.86005000
C	-0.85077500	3.33080200	-1.33525900
C	-3.47726300	4.35116400	-1.33282100
C	-1.11777900	4.60216200	-1.82859300
C	-2.42027900	5.12174700	-1.80797300
C	-2.34847100	-1.62310100	0.09856700
C	-2.81471300	-2.97083900	0.39018500
C	-3.40578900	-0.69003300	0.09324000
C	-4.22397000	-3.02480100	0.51882400
C	-2.08105900	-4.14737500	0.62662700
C	-4.89398700	-4.21428700	0.80758200
C	-2.74480600	-5.33334000	0.92246000
H	-0.99718900	-4.11798000	0.61482500
C	-4.14456000	-5.37252100	0.99761000
H	-5.97640300	-4.23349500	0.89510700
H	-2.17021000	-6.23672900	1.10631900
H	-4.64988100	-6.30712900	1.22384100
N	0.13412700	-1.86384700	-0.29886000
S	-4.42086600	1.88528300	-0.37717600
S	-4.98038600	-1.44040100	0.33926800
C	0.08118100	-2.69515800	-1.52859000
H	1.03113800	-3.20588500	-1.67434100
H	-0.70948800	-3.44041700	-1.44145000
H	-0.11934800	-2.06583500	-2.40523000
C	2.57469800	-1.21252800	-0.29481600
C	3.31370200	-2.46949700	-0.31192900
C	3.44568600	-0.10056200	-0.29547900
C	4.70593800	-2.26196400	-0.47139600
C	2.86477400	-3.78802900	-0.11074800
C	2.97776800	1.18269800	0.00719400
S	5.13481300	-0.55137700	-0.50969000
C	5.61209700	-3.32179400	-0.52469900
C	3.76649000	-4.84655800	-0.14561000
H	1.82140400	-3.97588500	0.11479200
C	1.60910500	1.42730700	0.25784500
S	4.00458500	2.58073300	0.32792400
C	5.13104600	-4.61957800	-0.37328300
H	6.67362200	-3.13592000	-0.65995500
H	3.40817500	-5.85917700	0.01637900
C	1.42274400	2.71824700	0.91030900
C	2.62501800	3.46325600	0.98057000
H	5.82307700	-5.45620500	-0.40637500
C	2.68396100	4.72184300	1.58133100
C	1.53048300	5.23137900	2.17066200
H	-4.49525700	4.72924700	-1.35496700
H	3.61675500	5.27732600	1.61310400
H	-2.61288500	6.12091500	-2.18863300
C	0.28462200	3.24223600	1.54977500
C	0.34378700	4.48354500	2.17142500
H	1.55948300	6.20418900	2.65348700
H	-0.63471200	2.67019900	1.57256400
H	-0.54078900	4.87608100	2.66441000
H	-0.30583600	5.19703900	-2.23663800
H	0.15689300	2.93771000	-1.37847100

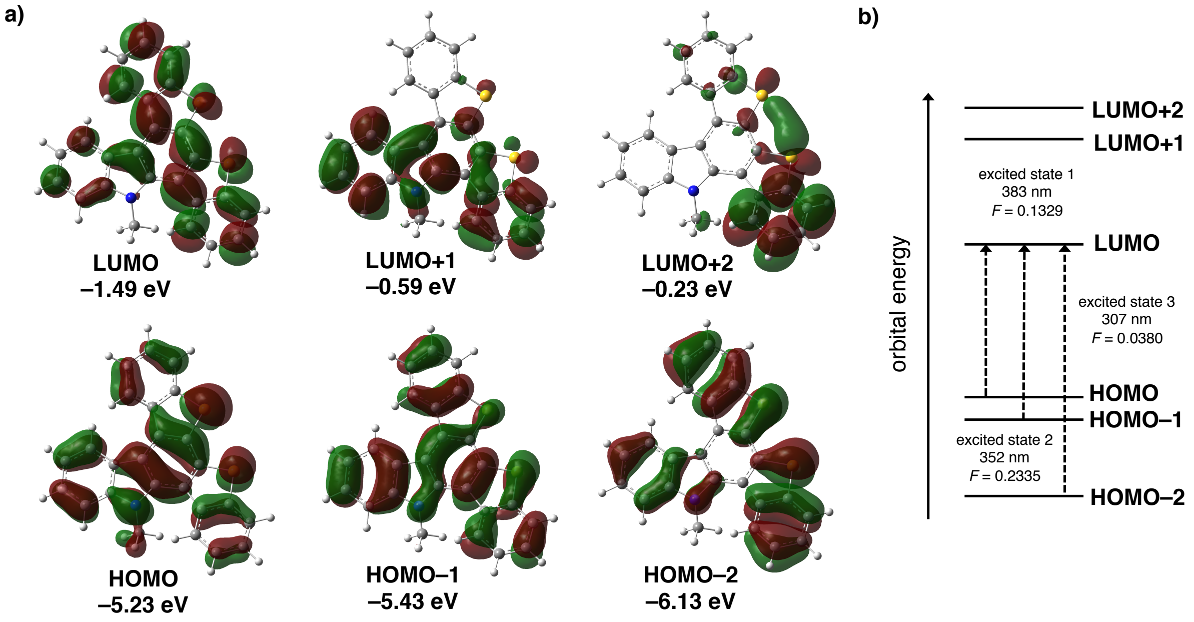

Figure S4. Pictorial representations of the frontier MOs of **8** and energy diagrams and, calculated at the B3LYP/6-31G(d) level of theory.

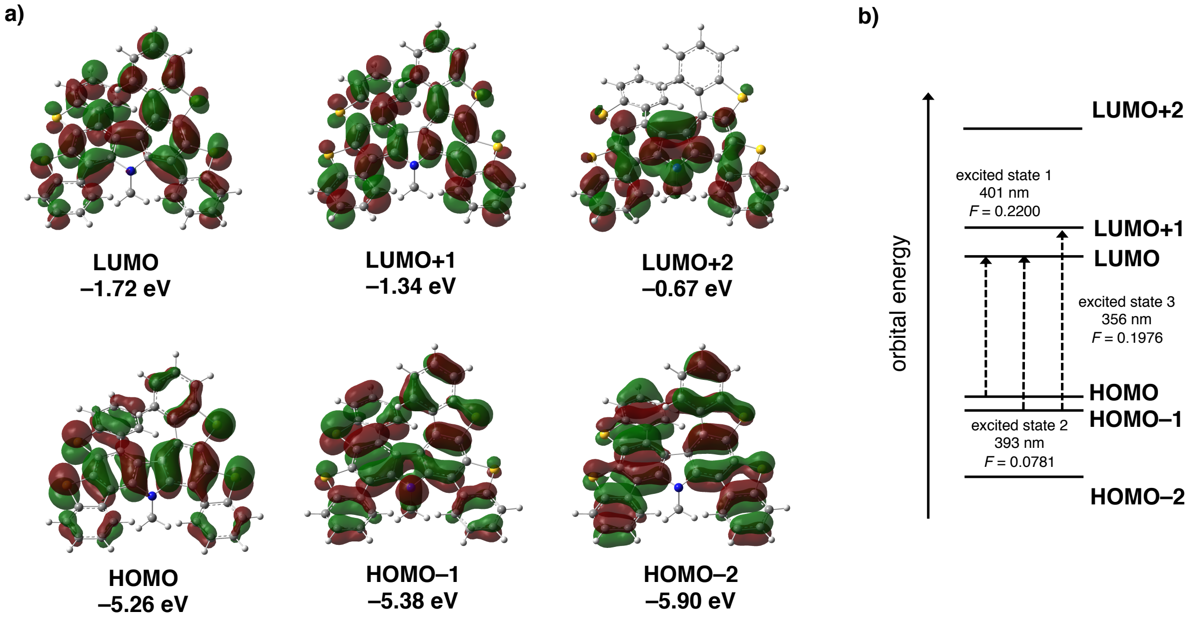

Figure S5. Pictorial representations of the frontier MOs of **9** and energy diagrams and, calculated at the B3LYP/6-31G(d) level of theory.

Table S7. TD-DFT vertical one-electron excitations (3 states) calculated for the conformation of optimized **8**.

exited state	energy	wavelength	oscillator strength (*f*)	description
1	3.2338 eV	383.41 nm	0.1329	**HOMO → LUMO (0.68377)**<br>HOMO–1 → LUMO+1 (0.13101)
2	3.5204 eV	352.19 nm	0.2335	**HOMO–1 → LUMO (0.67894)**<br>HOMO → LUMO+1 (–0.12685)
3	4.0345 eV	307.31 nm	0.0380	**HOMO–2 → LUMO (0.63711)**<br>HOMO–1 → LUMO+1 (0.10179)<br>HOMO → LUMO+1 (–0.19606)

Table S8. TD-DFT vertical one-electron excitations (5 states) calculated for the conformation of optimized **9**.

exited state	energy	wavelength	oscillator strength (*f*)	description
1	3.0922 eV	400.96 nm	0.2200	**HOMO → LUMO (0.67912)**<br>HOMO–1 → LUMO (0.12453)
2	3.1538 eV	393.13 nm	0.0781	**HOMO–1 → LUMO (0.62281)**<br>HOMO → LUMO (–0.13802)<br>HOMO → LUMO+1 (0.27352)
3	3.4845 eV	355.82 nm	0.1976	HOMO–3 → LUMO (0.10794)<br>HOMO–1 → LUMO (–0.27528)<br>**HOMO → LUMO+1 (0.62304)**
4	3.5461 eV	349.63 nm	0.1649	**HOMO–1 → LUMO+1 (0.68477)**<br>HOMO → LUMO+2 (0.12258)
5	3.6880 eV	336.19 nm	0.0296	**HOMO–2 → LUMO (0.66399)**<br>HOMO → LUMO+2 (0.19397)

7. Absorption and emission spectra of 8 and 9

UV–vis absorption spectra in CH_2Cl_2 were recorded at room temperature on a Shimadzu UV-3510 spectrometer with a resolution of 0.2 nm. Emission spectra were measured on Shimadzu RF-6000 spectrometer in CH_2Cl_2 at room temperature with a resolution of 0.1 nm upon excitation at 427 nm for **8** and 437 nm for **9**. Absolute fluorescence quantum yields (Φ_F) were determined on a Shimadzu RF-6000 using a calibrated integrating sphere system (207-21460-41). Dilute solution in degassed spectral grade CH_2Cl_2 in a 1 cm square quartz cell was used for all measurements

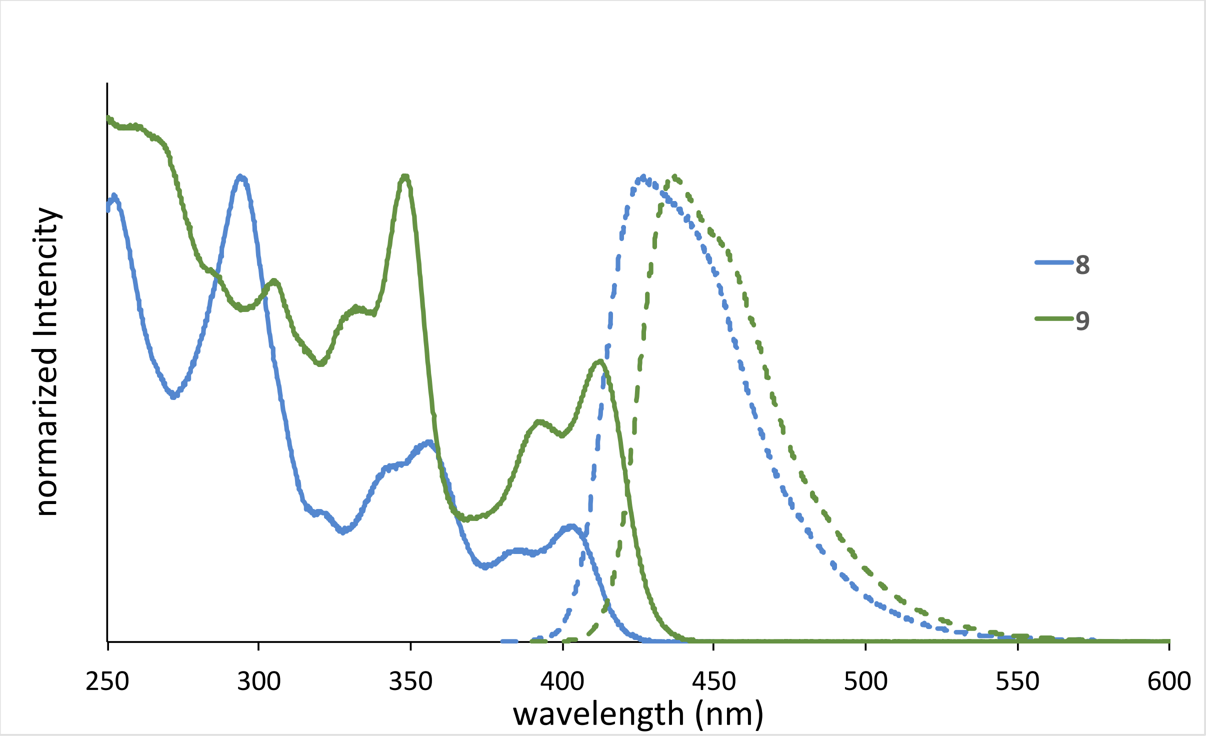

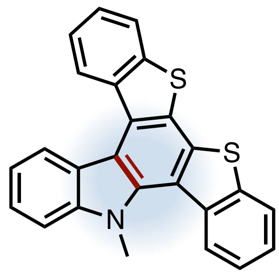

8

Φ_F = 7%

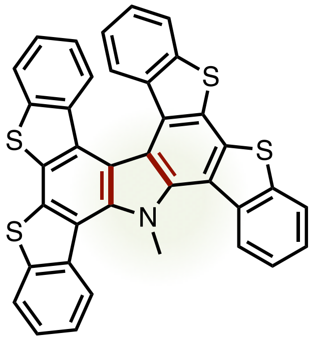

9

Φ_F = 5%

8. ^{1}H and ^{13}C NMR spectra

9-Methyl-9*H*-dibenzo[*a*,*c*]carbazole (3aa)

^{1}H NMR (600 MHz, $CDCl_3$)

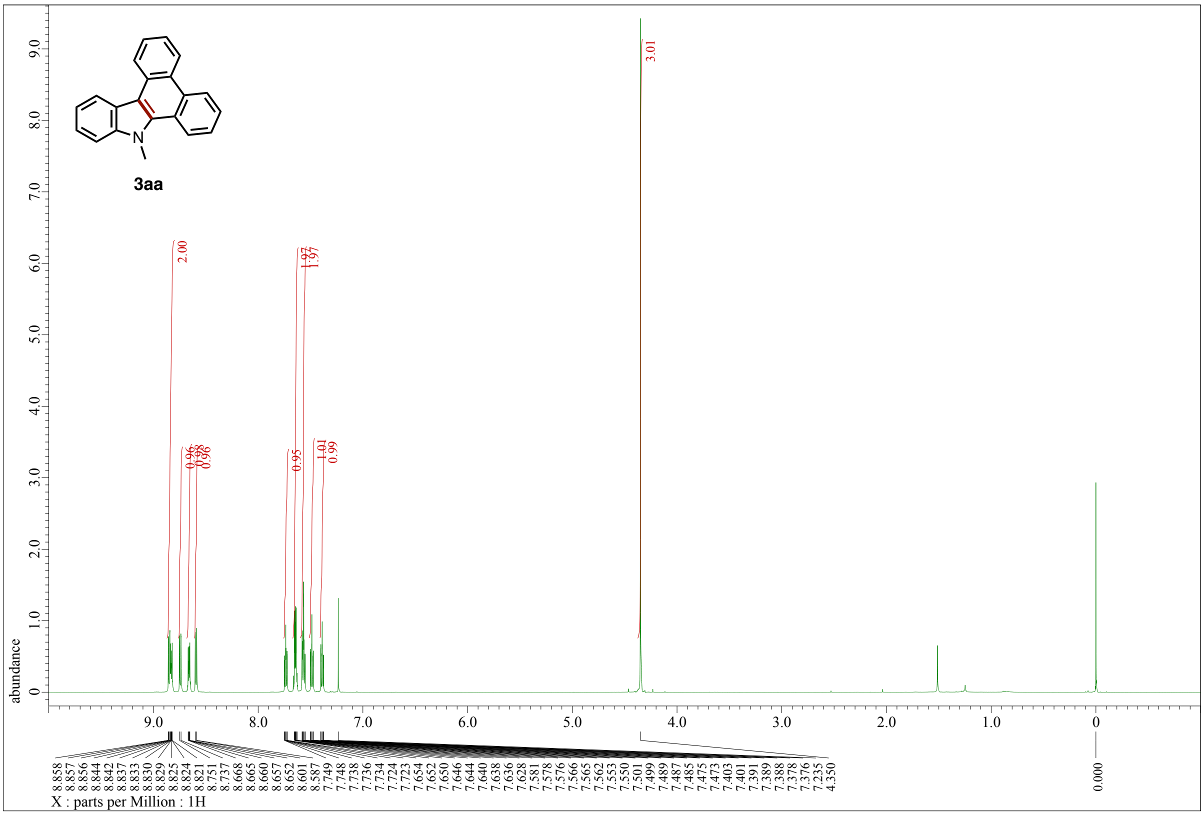

^{13}C NMR (150 MHz, $CDCl_3$)

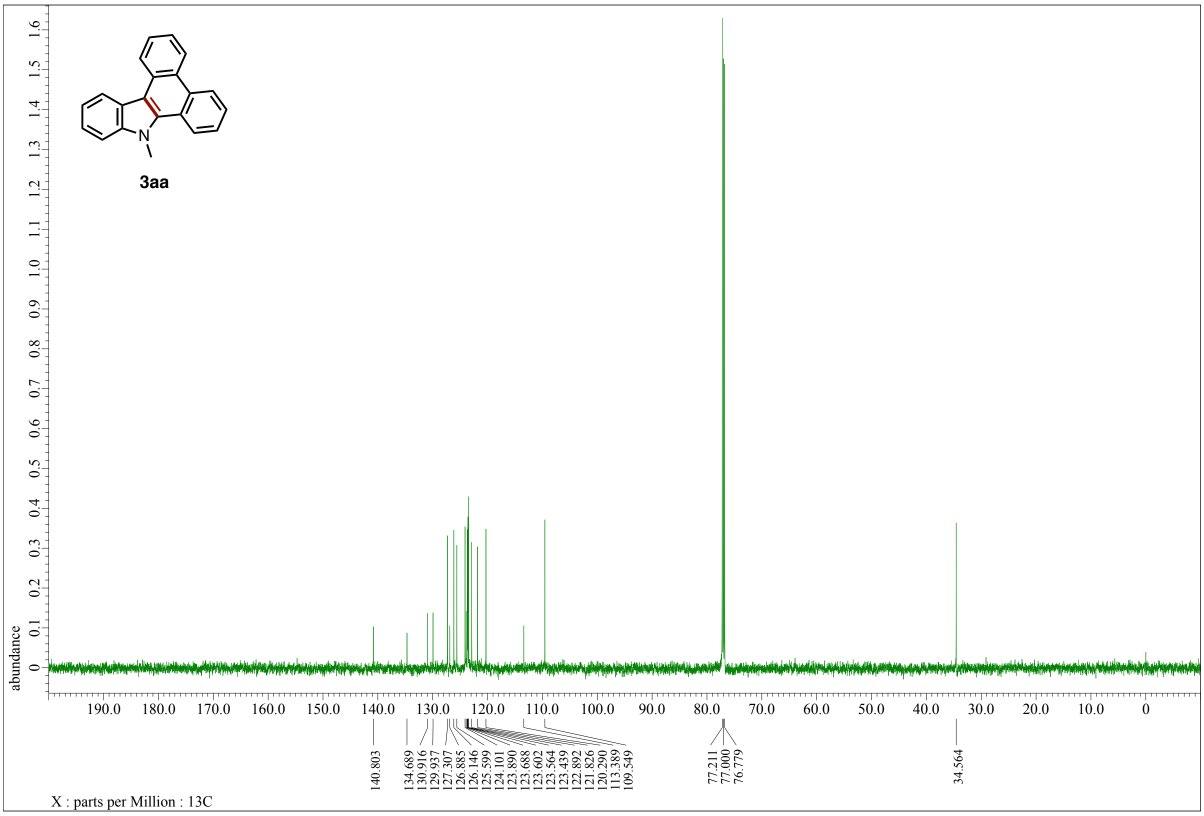

9-Ethyl-9*H*-dibenzo[*a*,*c*]carbazole (3ba)

^{1}H NMR (600 MHz, $CDCl_3$)

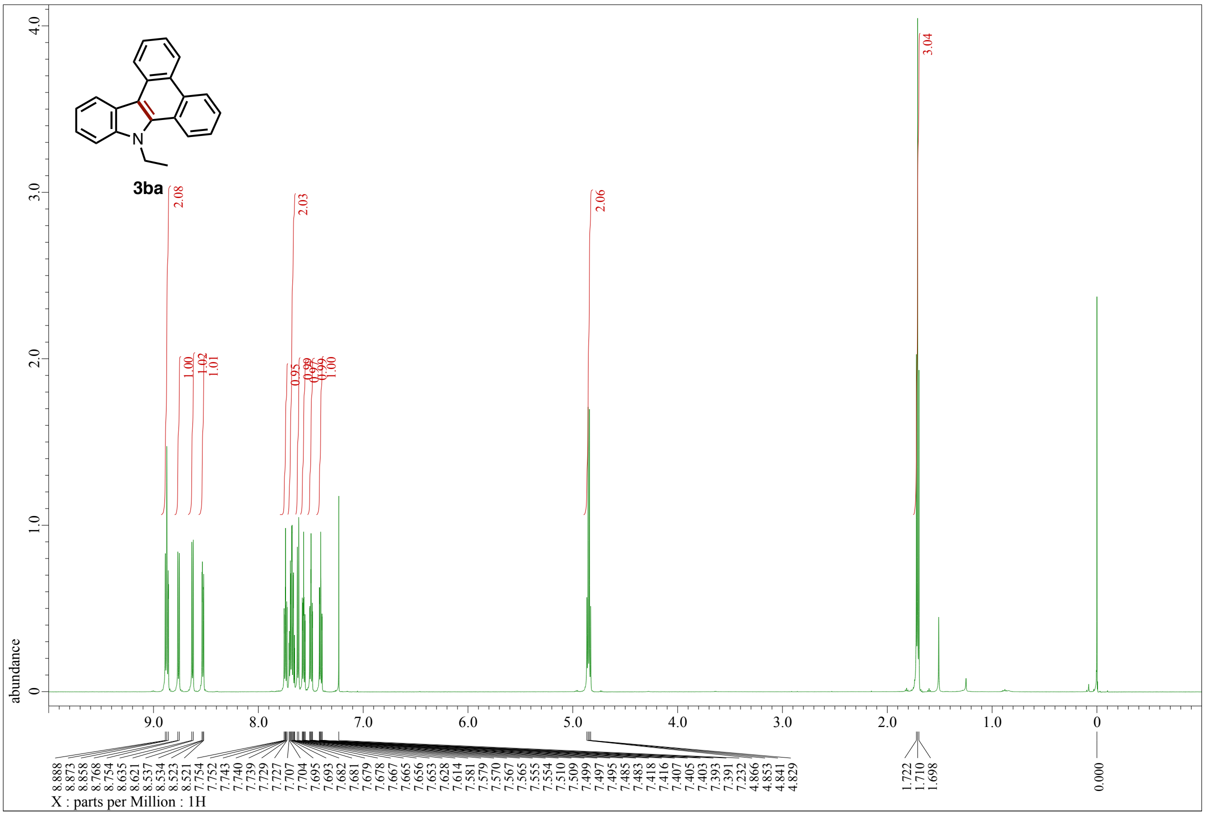

^{13}C NMR (150 MHz, $CDCl_3$)

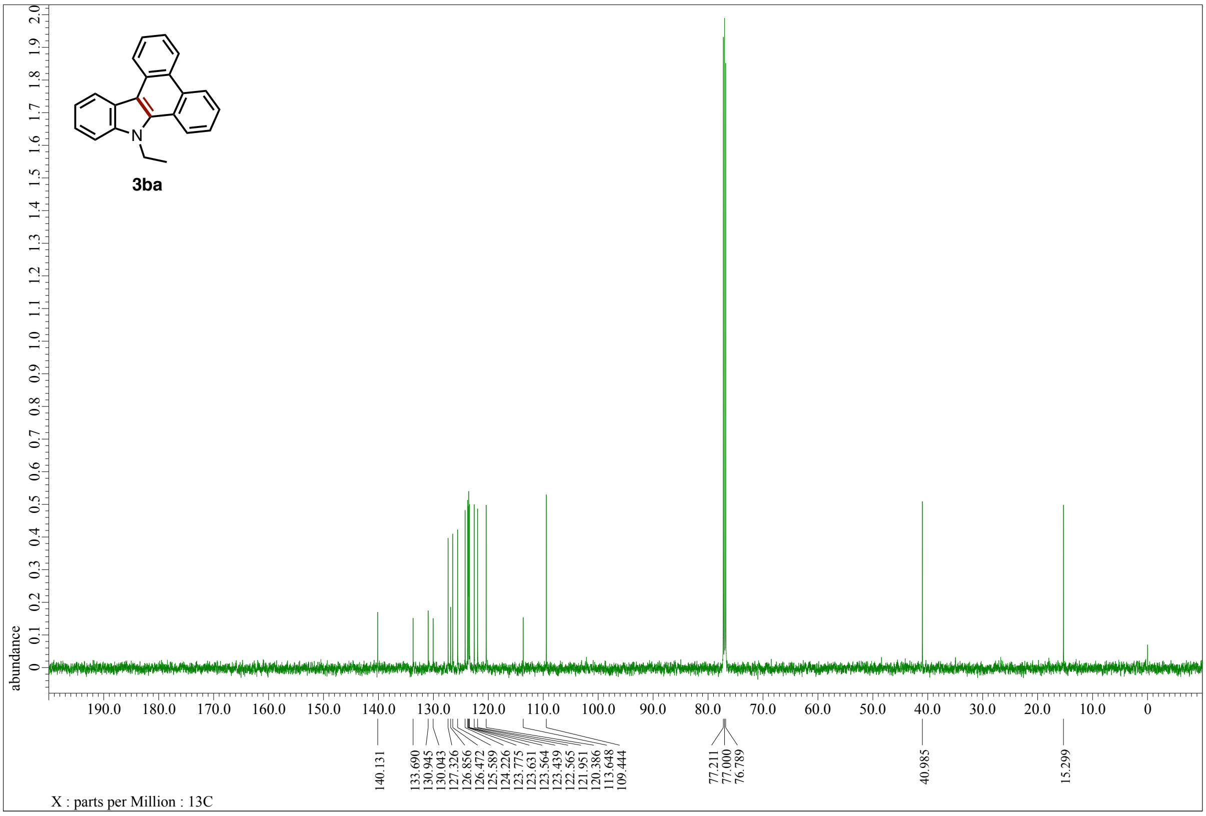

9-Benzyl-9*H*-dibenzo[*a*,*c*]carbazole (3ca)

^{1}H NMR (600 MHz, CDCl$_3$)

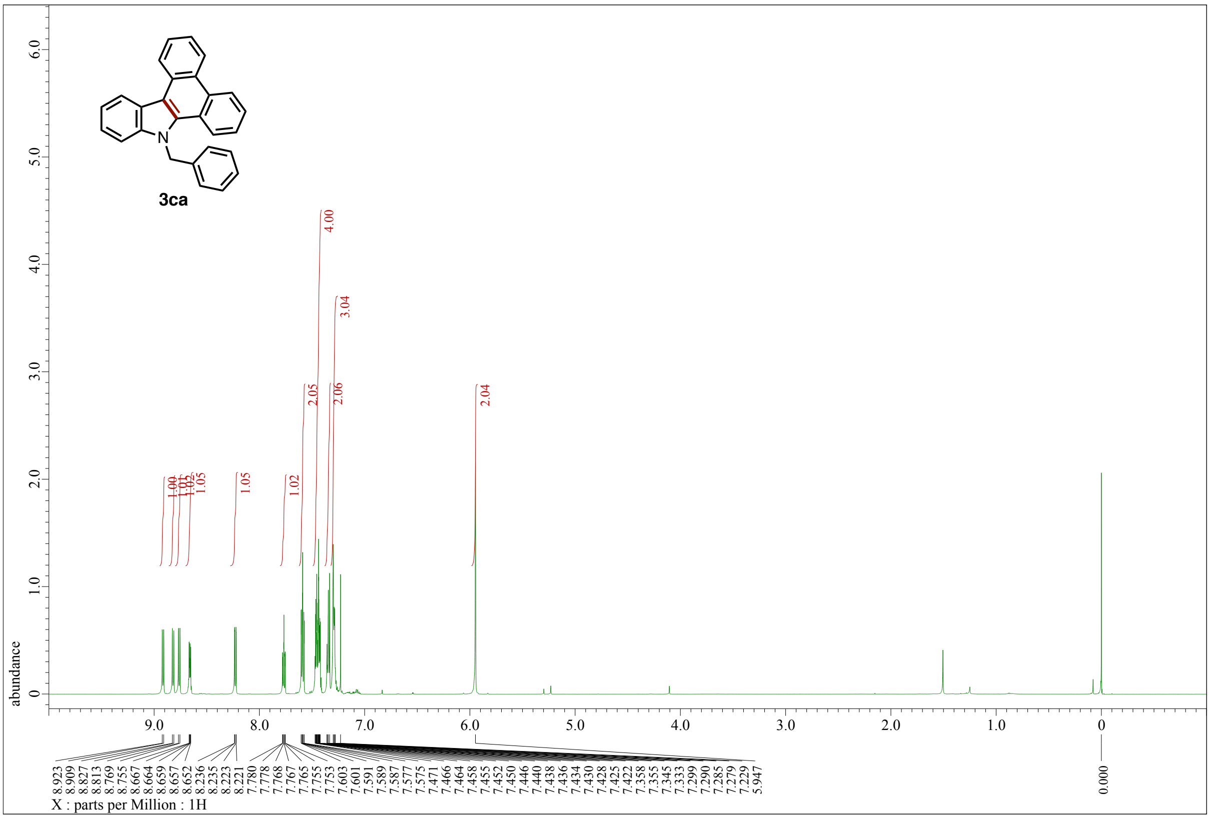

^{13}C NMR (150 MHz, CDCl$_3$)

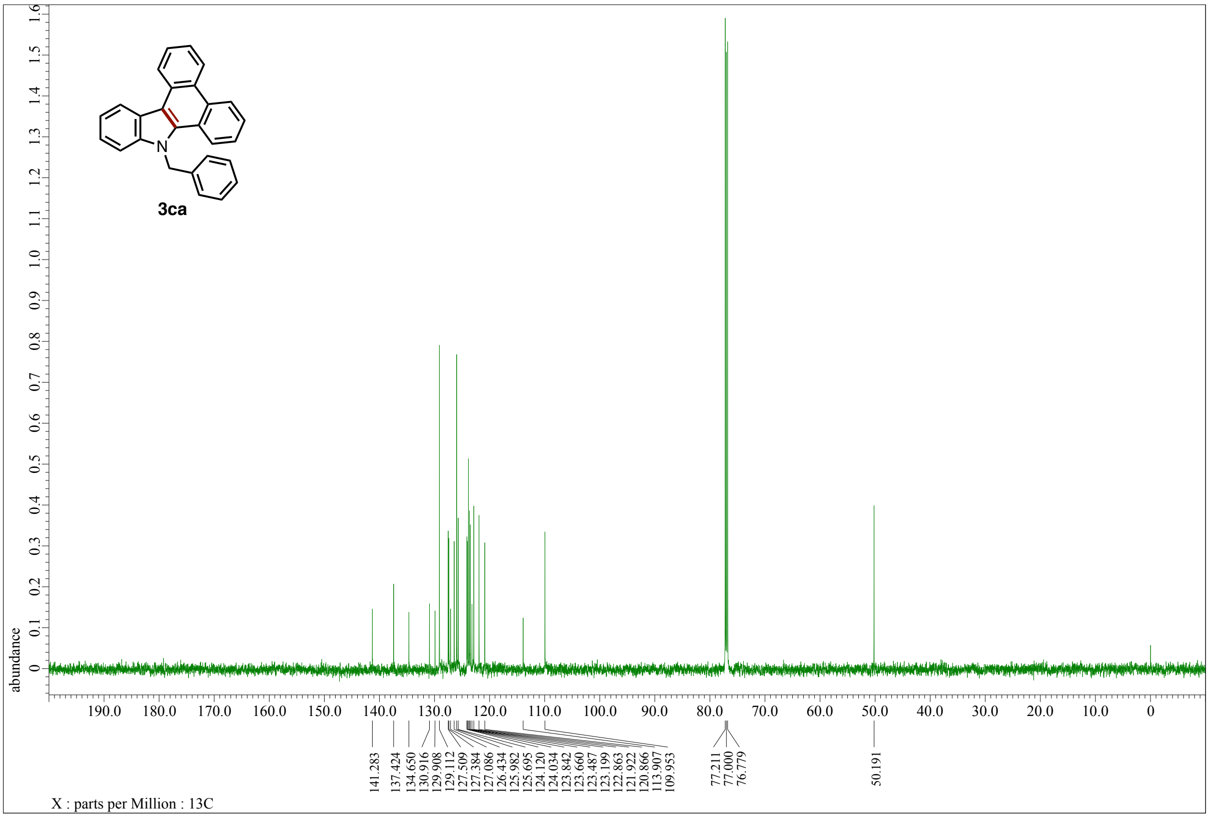

9-Phenyl-9*H*-dibenzo[*a*,*c*]carbazole (3da)

^{1}H NMR (600 MHz, $CDCl_3$)

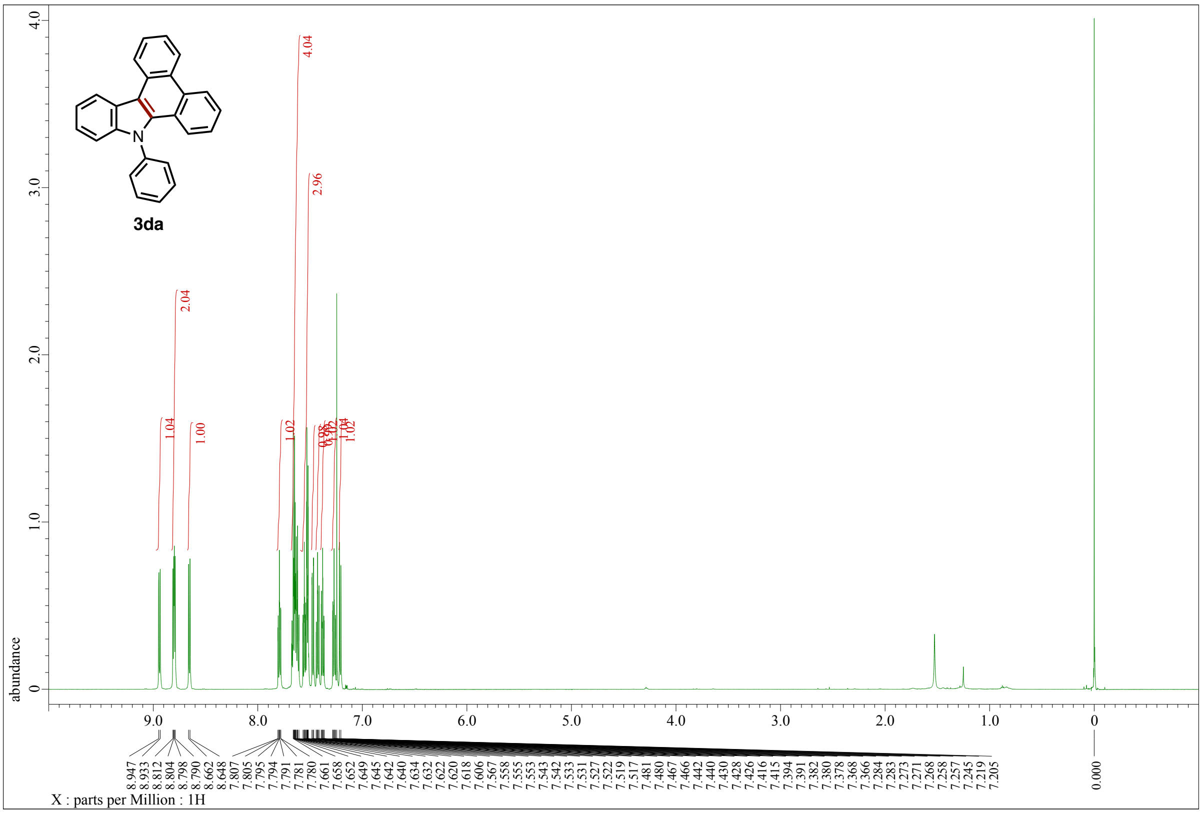

^{13}C NMR (150 MHz, $CDCl_3$)

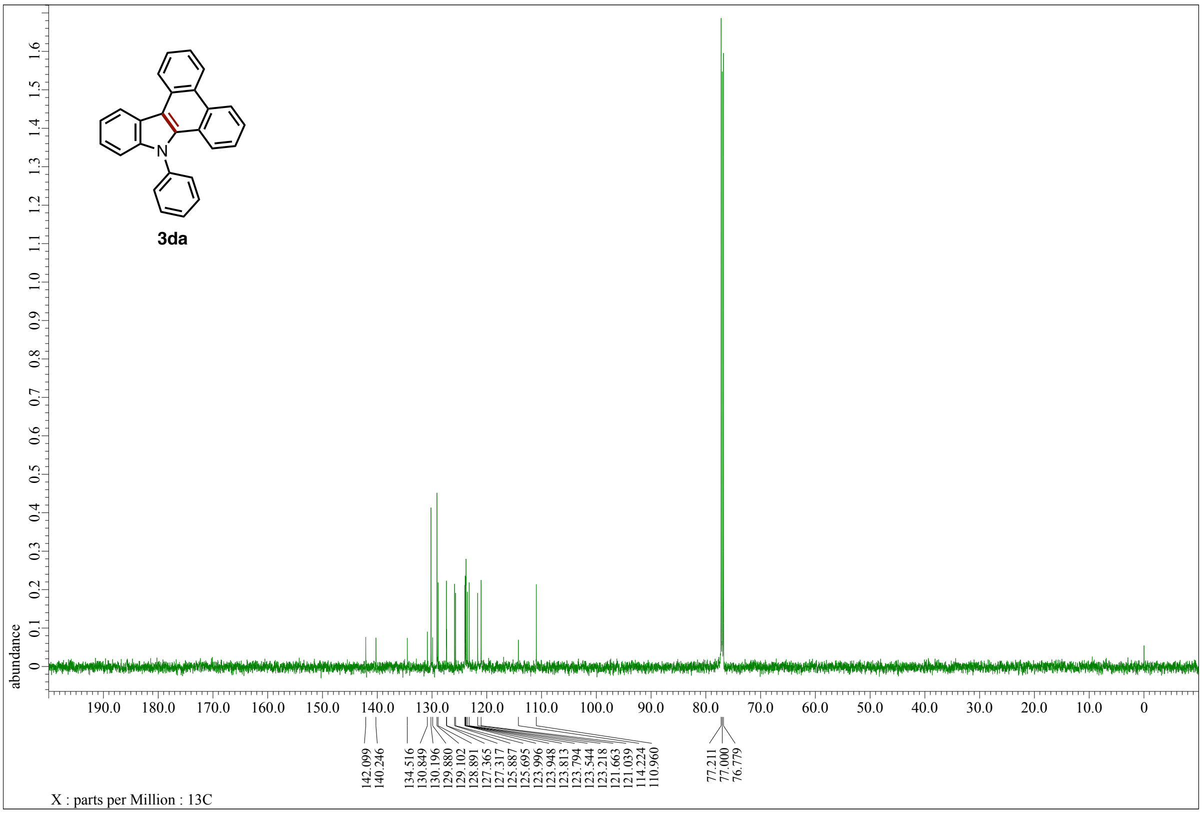

5,6-Dihydro-4*H*-dibenzo[*a*,*c*]pyrido[3,2,1-*jk*]carbazole (3ea)

^{1}H NMR (600 MHz, $CDCl_3$)

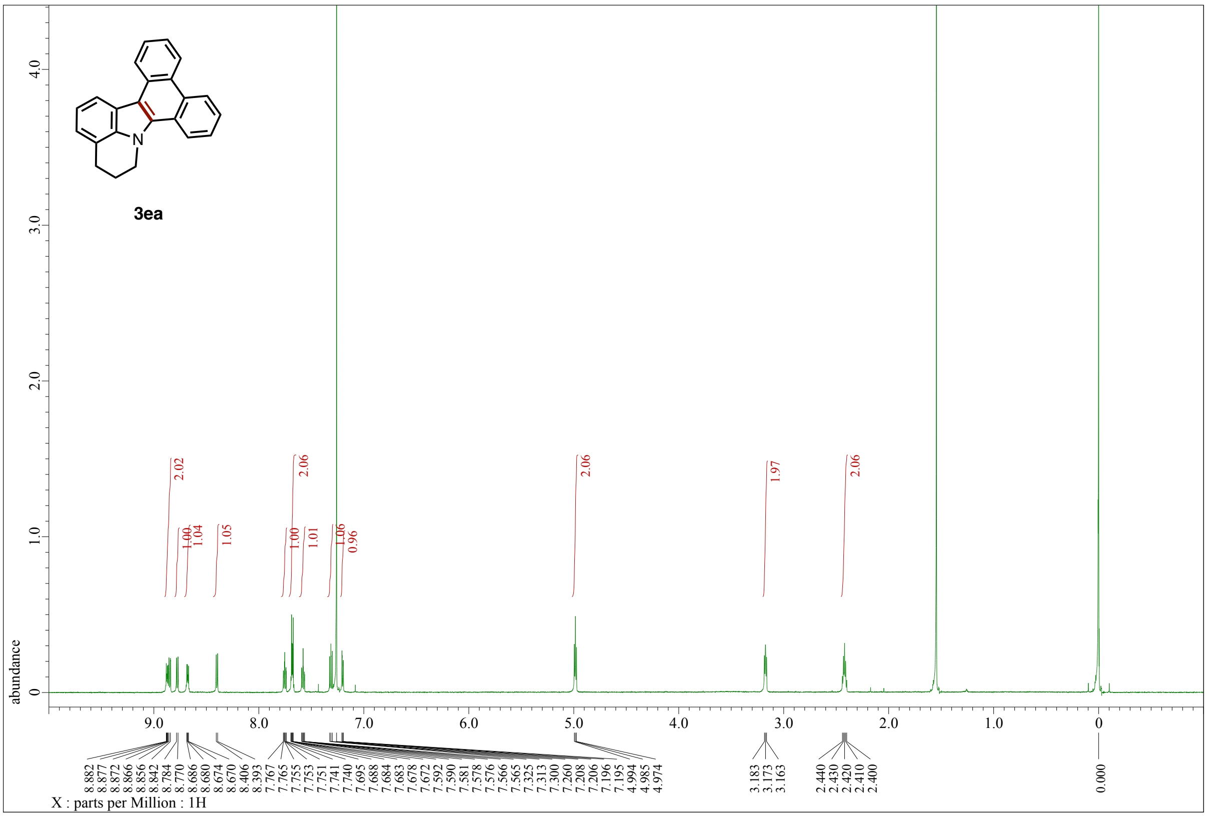

^{13}C NMR (150 MHz, $CDCl_3$)

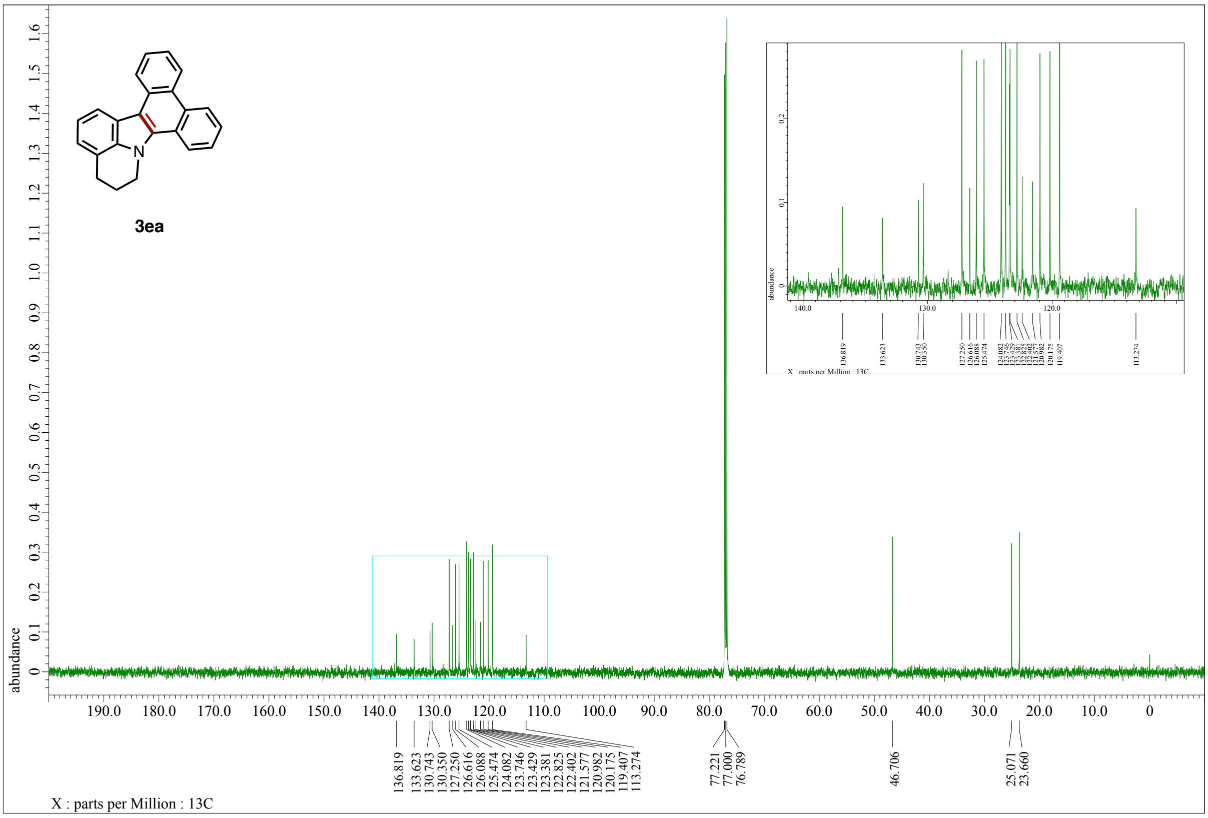

9-Benzyl-12-nitro-9*H*-dibenzo[*a*,*c*]carbazole (3ga)

^{1}H NMR (600 MHz, $CDCl_3$)

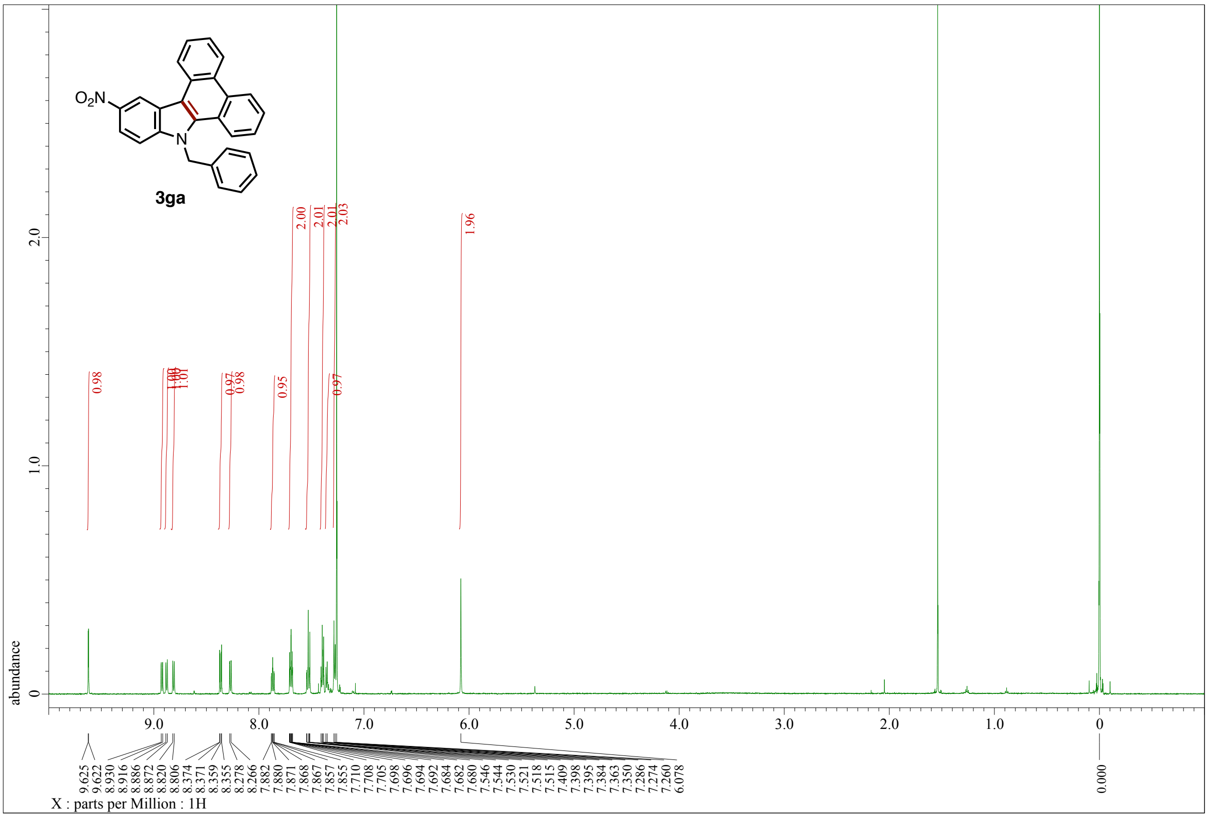

^{13}C NMR (150 MHz, $CDCl_3$)

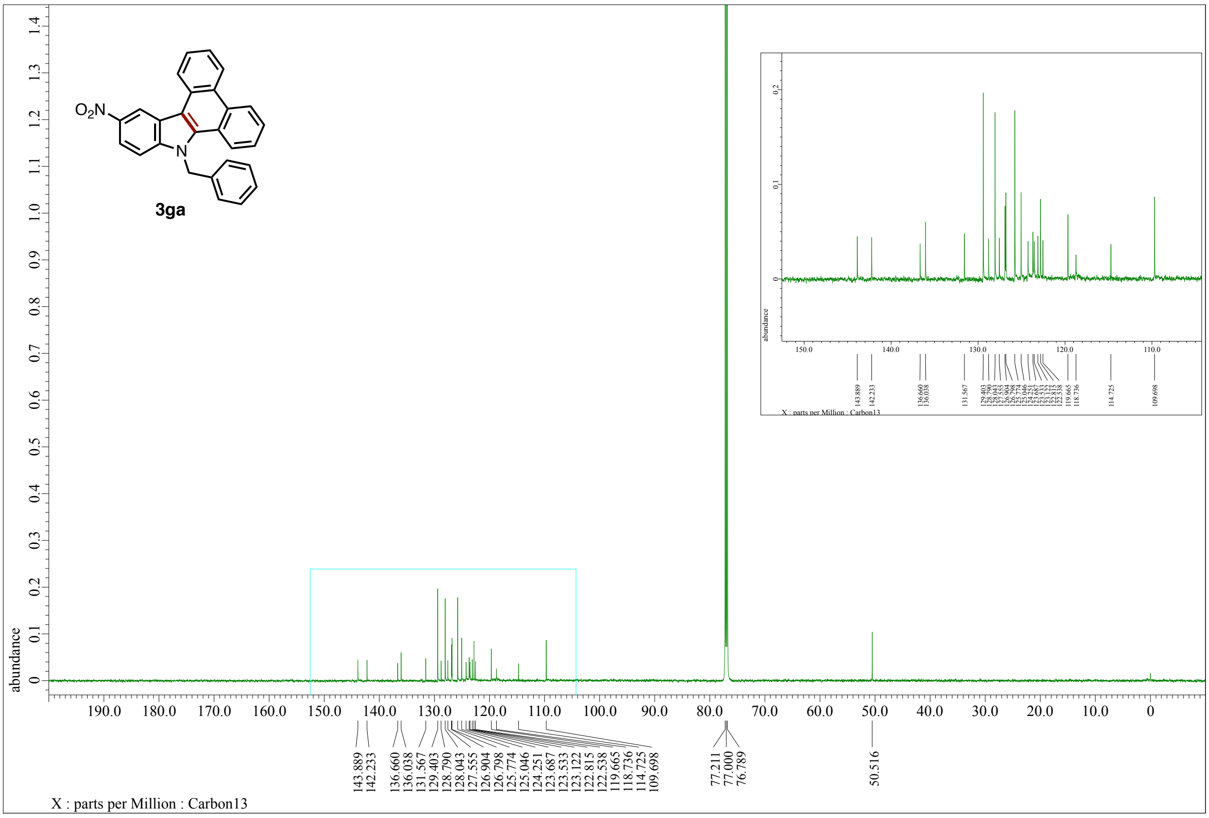

9-Benzyl-9*H*-dibenzo[*a*,*c*]carbazole-12-carbonitrile (3ha)

^{1}H NMR (600 MHz, $CDCl_3$)

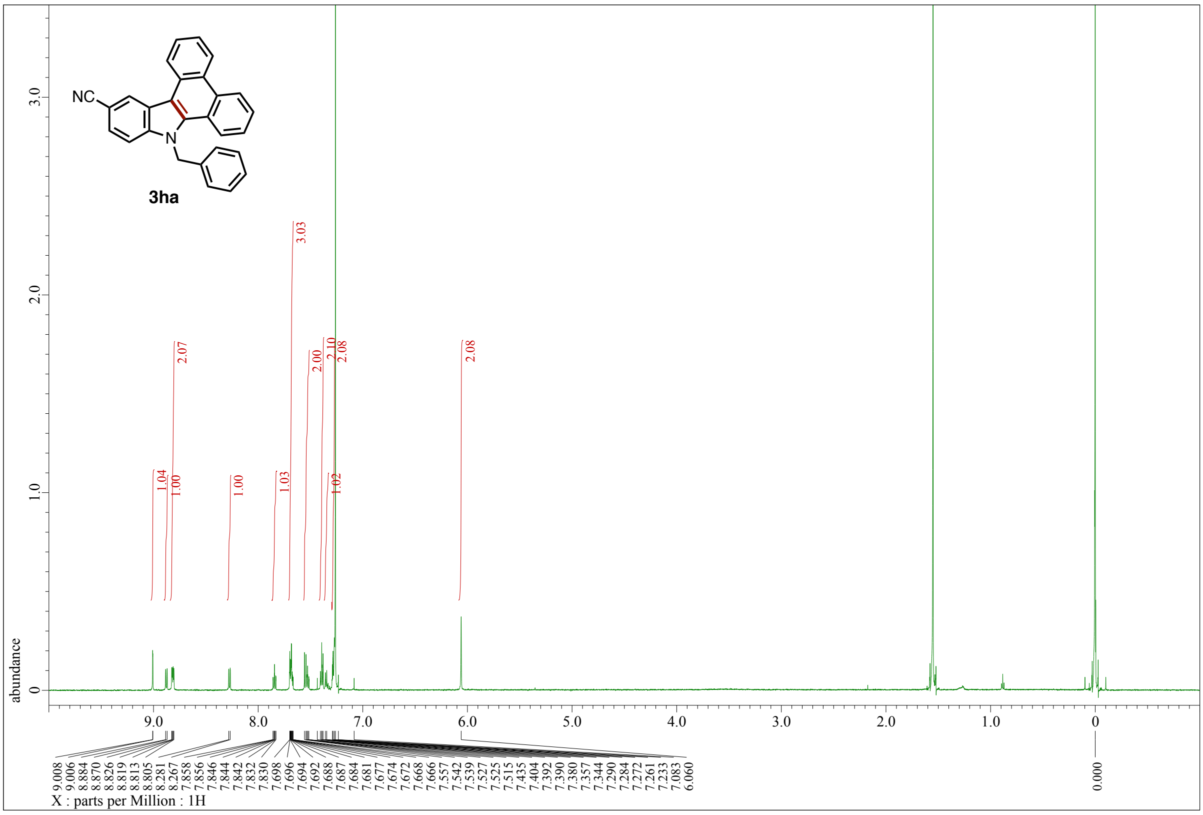

^{13}C NMR (150 MHz, $CDCl_3$)

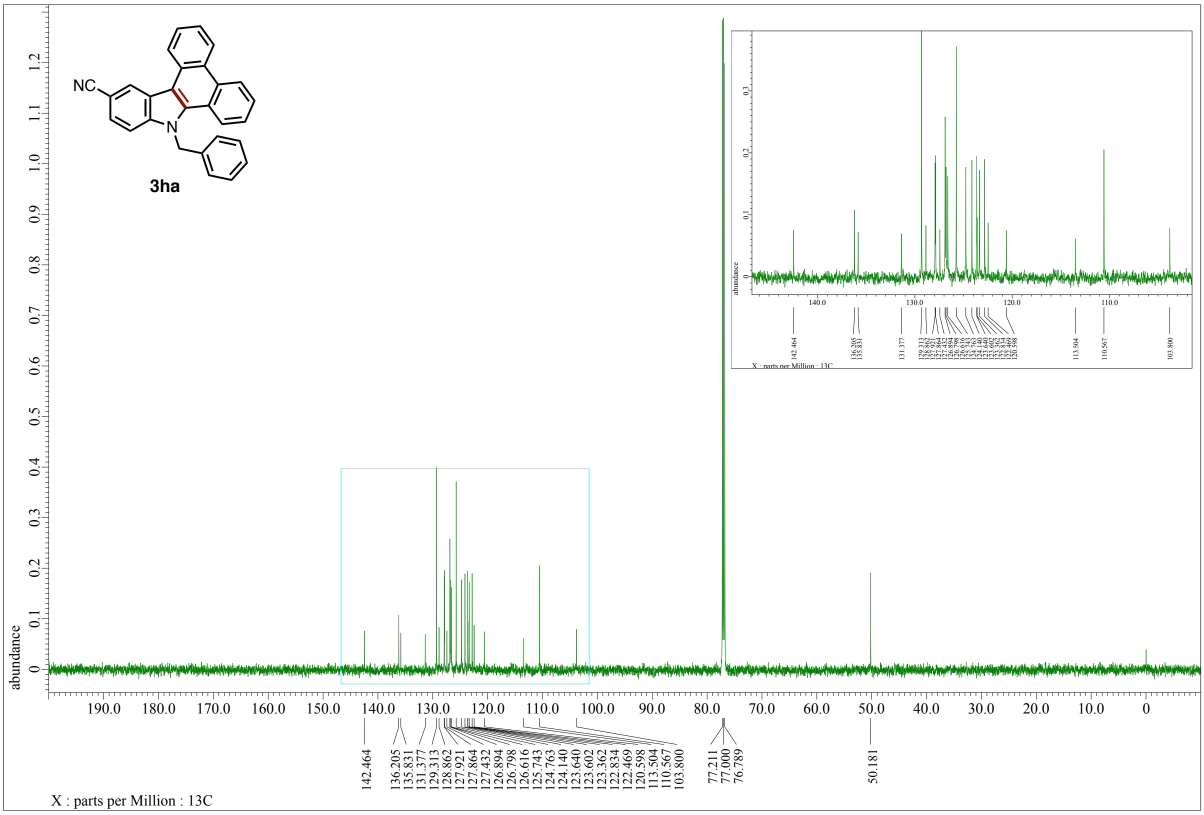

9-Benzyl-12-bromo-9*H*-dibenzo[*a*,*c*]carbazole (3ia)

^{1}H NMR (600 MHz, $CDCl_3$)

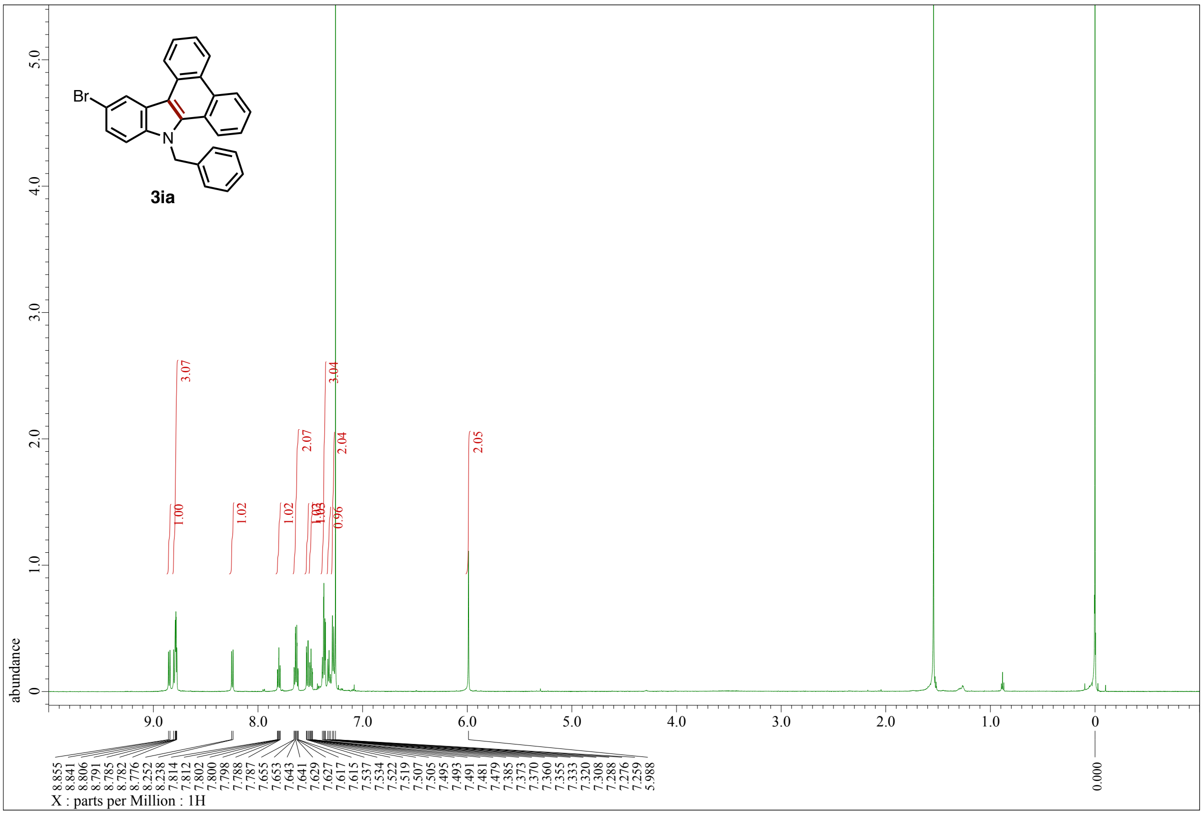

^{13}C NMR (150 MHz, $CDCl_3$)

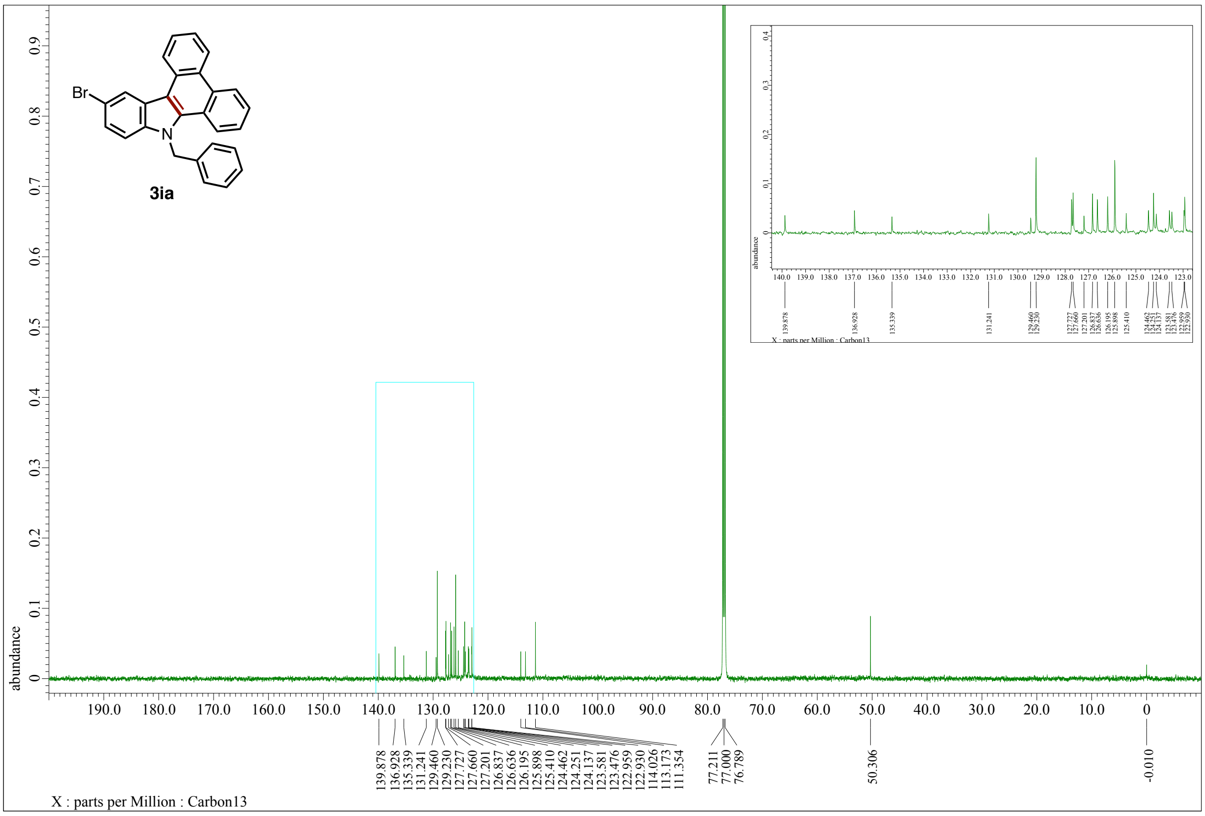

9-Benzyl-12-methoxy-9*H*-dibenzo[*a*,*c*]carbazole (3ja)

^{1}H NMR (600 MHz, $CDCl_3$)

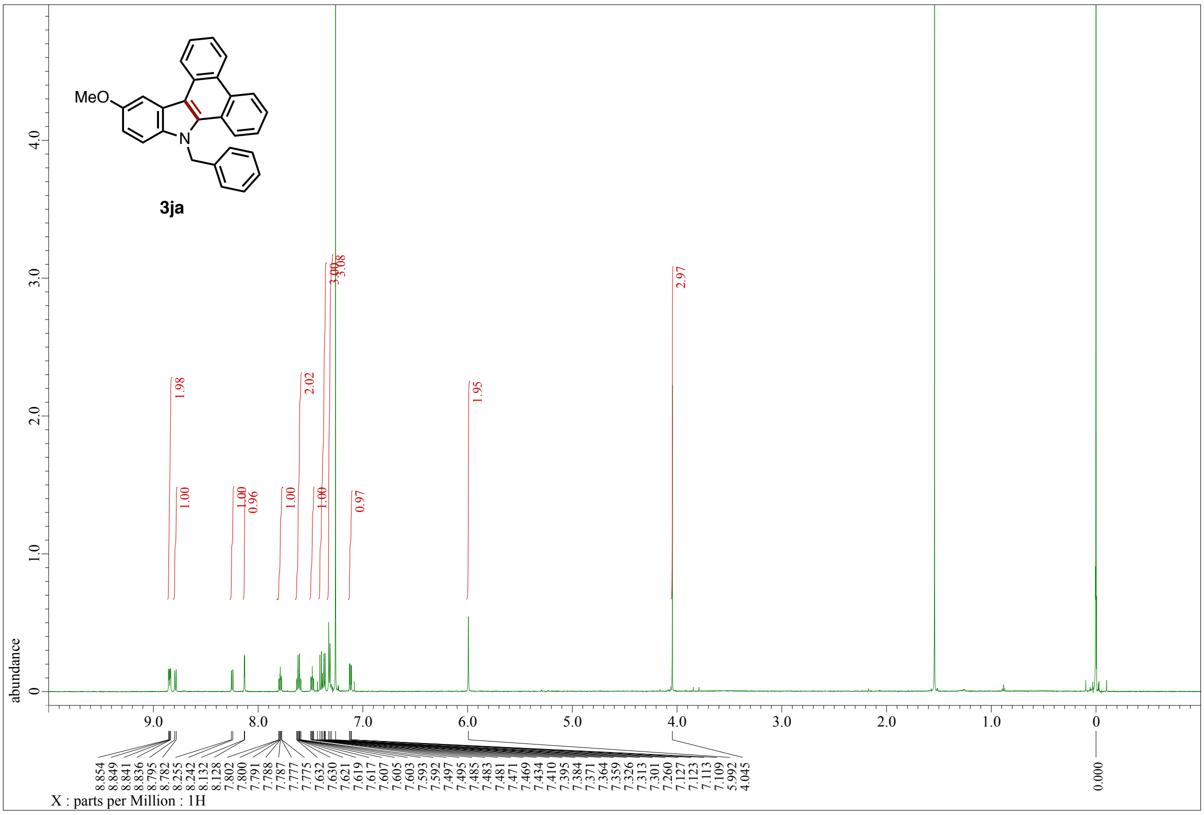

^{13}C NMR (150 MHz, $CDCl_3$)

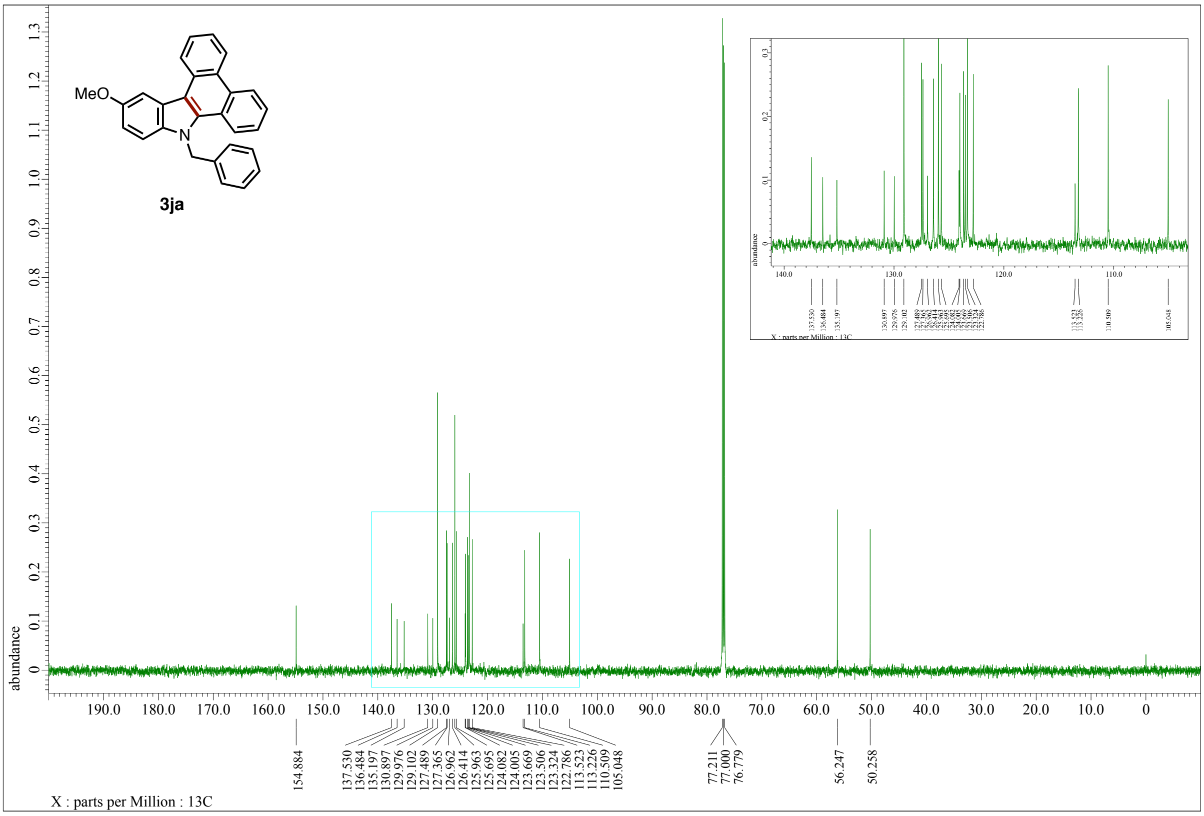

9-Benzyl-11-methoxy-9*H*-dibenzo[*a*,*c*]carbazole (3ka)

^{1}H NMR (600 MHz, CDCl$_3$)

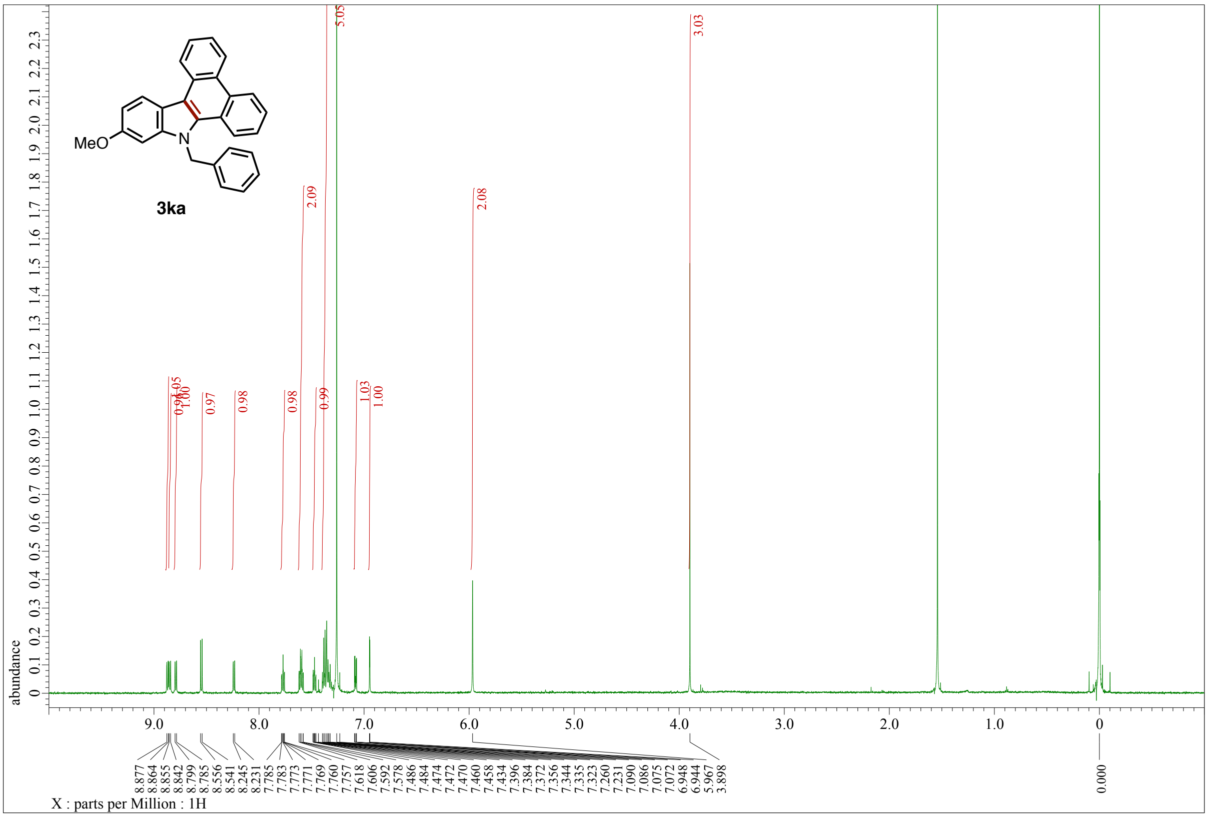

^{13}C NMR (150 MHz, CDCl$_3$)

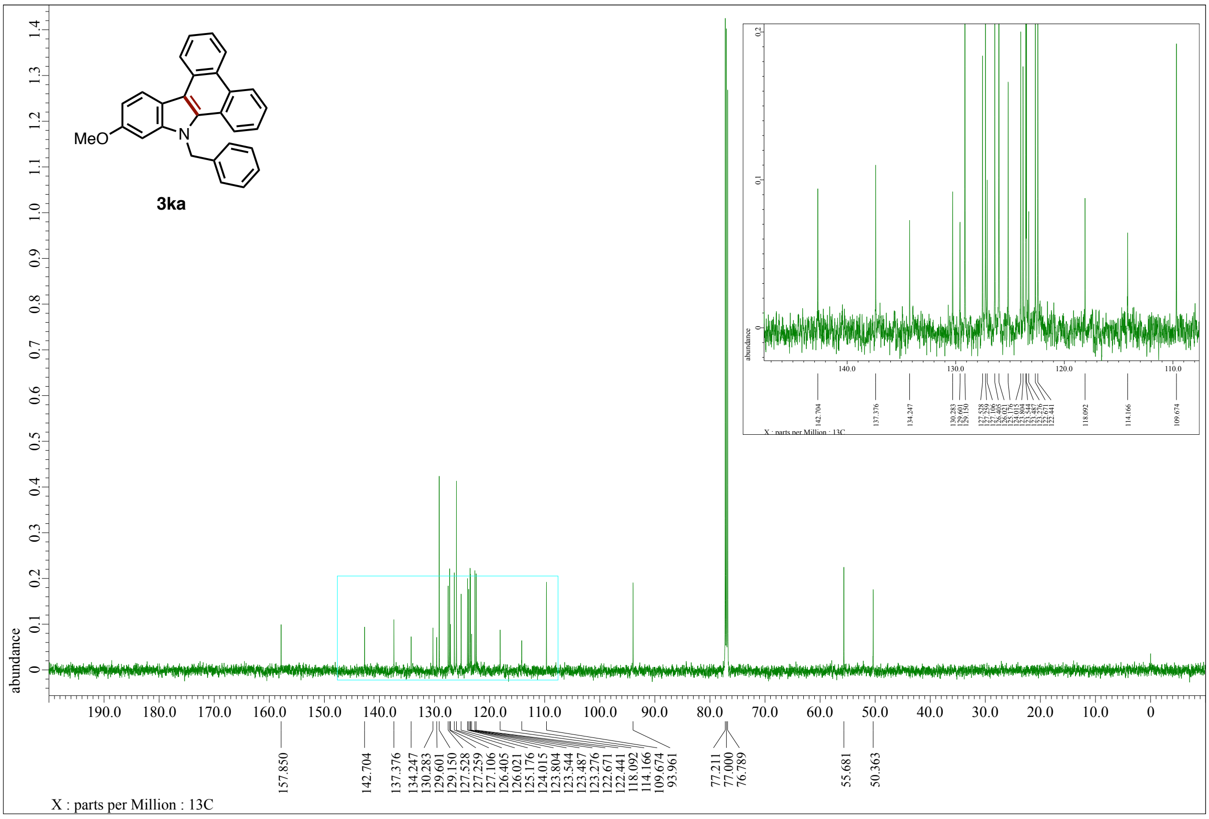

9-Benzyl-9*H*-dibenzo[*a*,*c*]carbazole-10-carbonitrile (3la)

^{1}H NMR (600 MHz, $CDCl_3$)

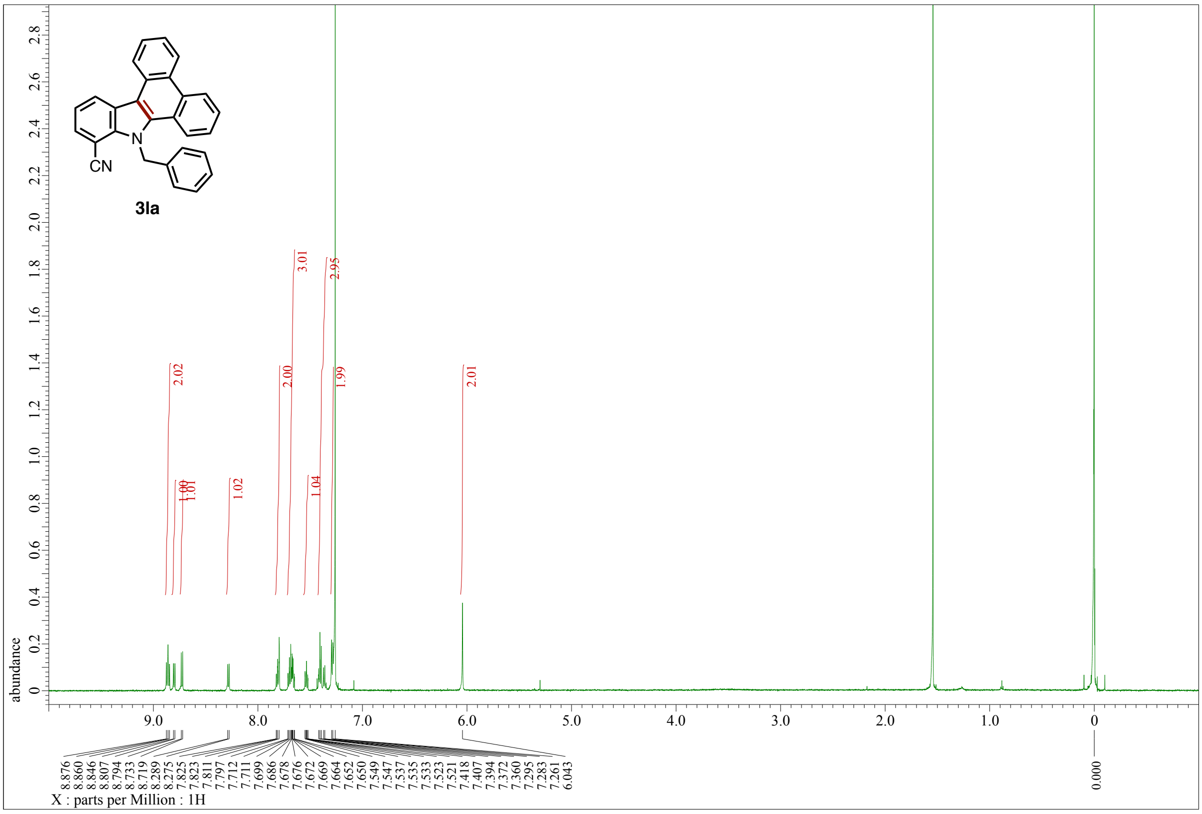

^{13}C NMR (150 MHz, $CDCl_3$)

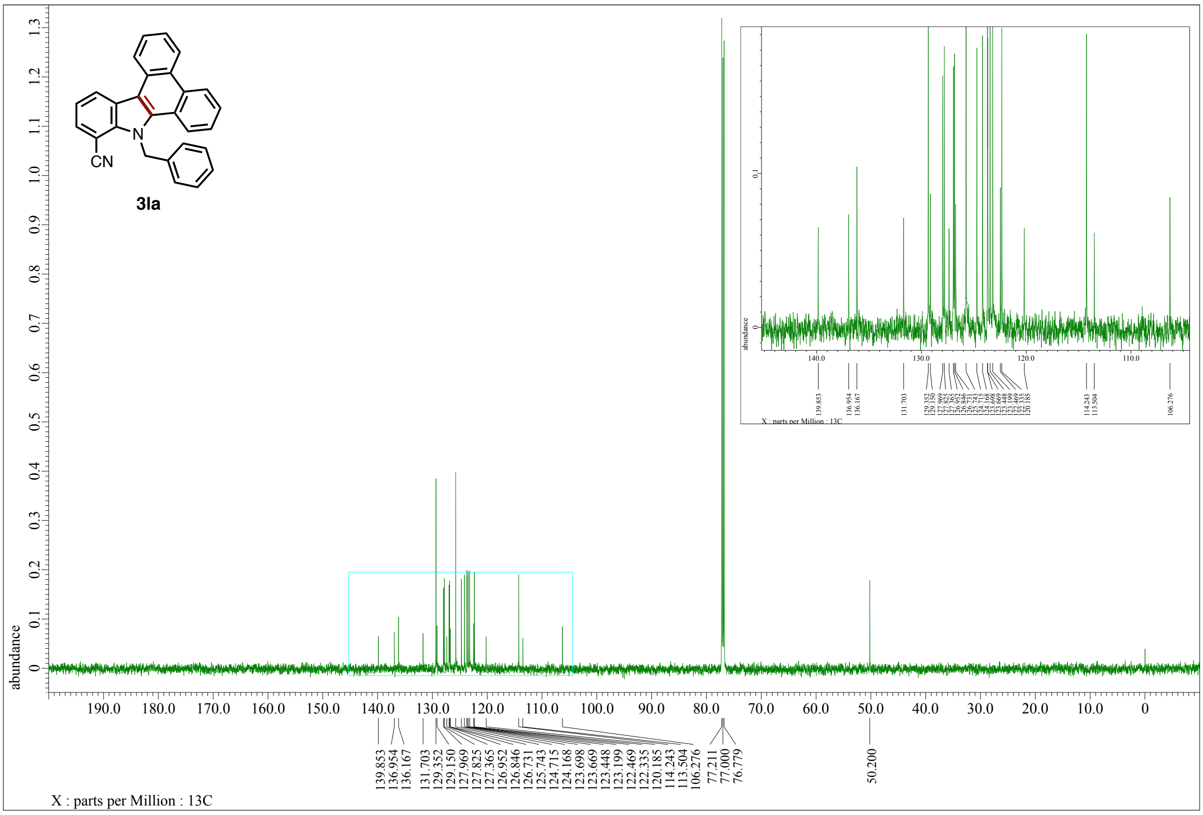

9-Benzyl-10-(benzyloxy)-9*H*-dibenzo[*a*,*c*]carbazole (3ma)

^{1}H NMR (600 MHz, $CDCl_3$)

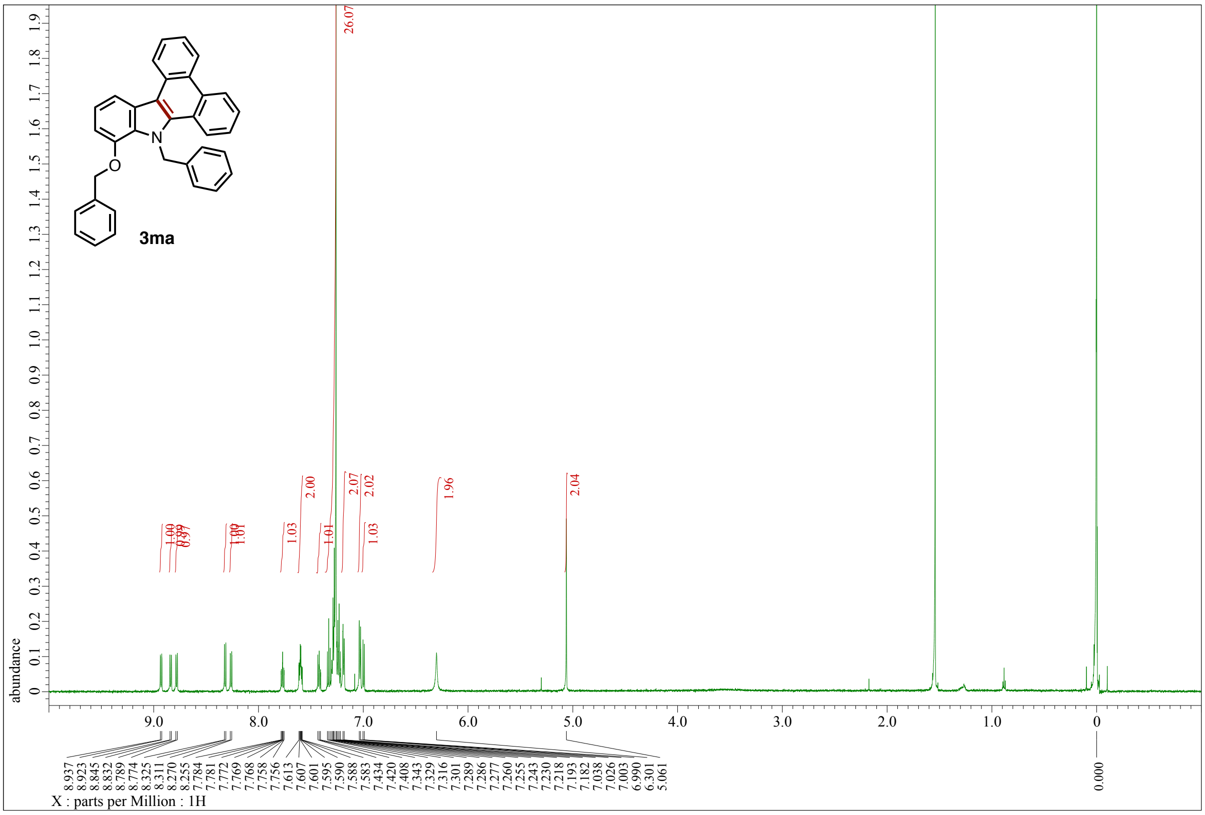

^{13}C NMR (150 MHz, $CDCl_3$)

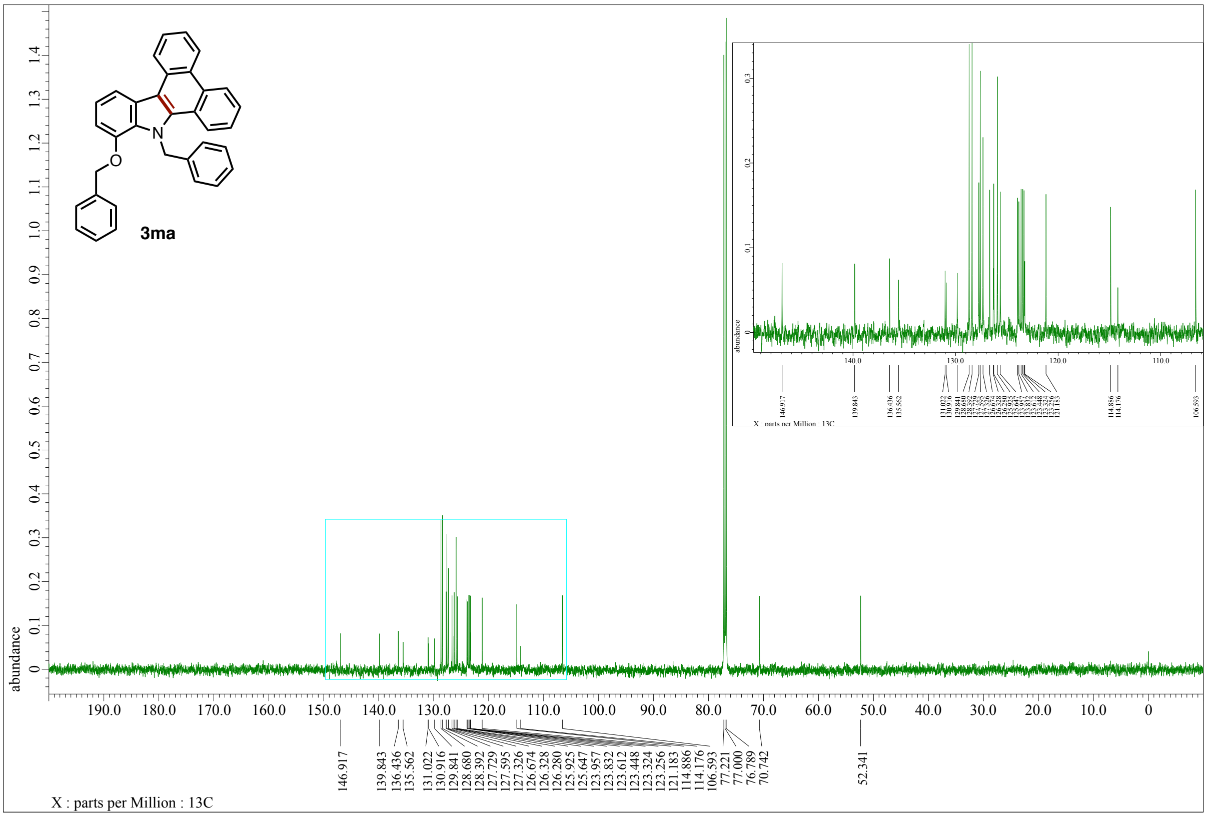

1-Ethyl-1*H*-dibenzo[*e*,*g*]indole (5aa)

^{1}H NMR (600 MHz, $CDCl_3$)

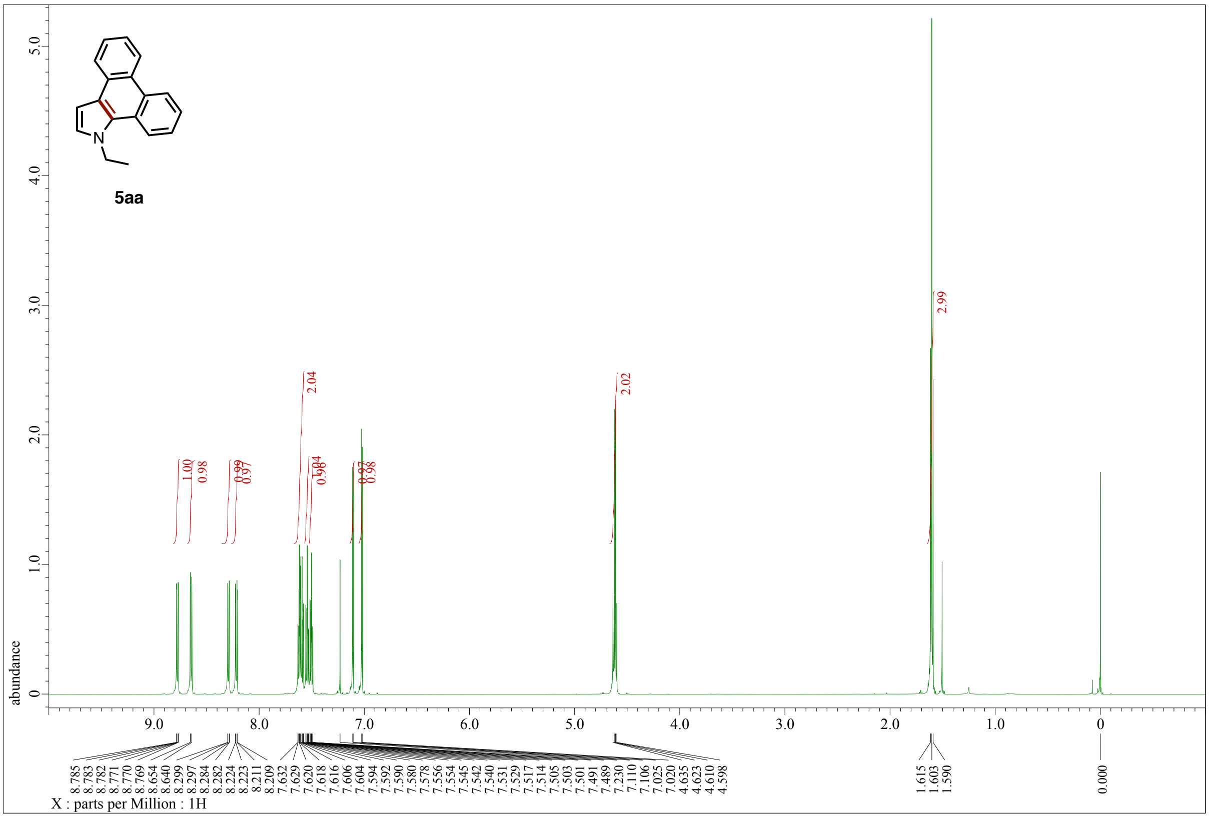

^{13}C NMR (150 MHz, $CDCl_3$)

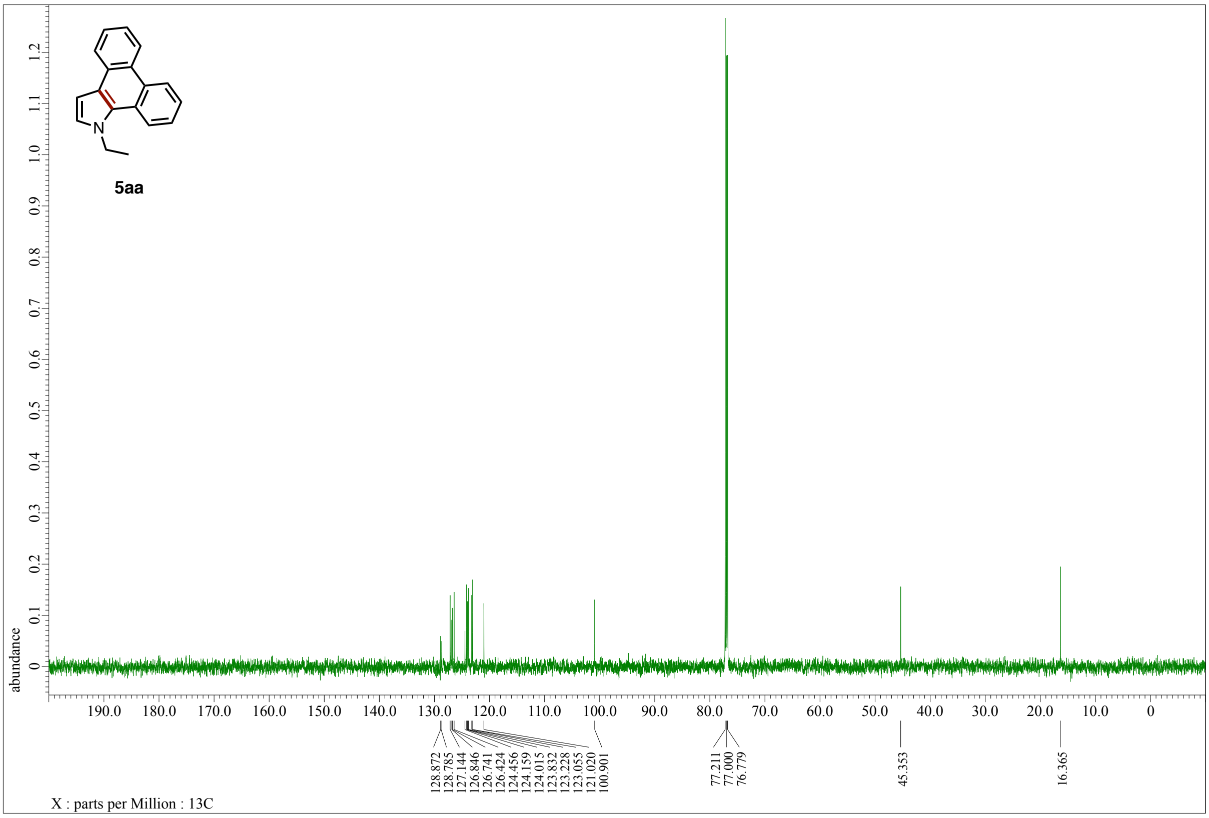

1-Benzyl-1*H*-dibenzo[*e*,*g*]indole (5ba)

^{1}H NMR (600 MHz, $CDCl_3$)

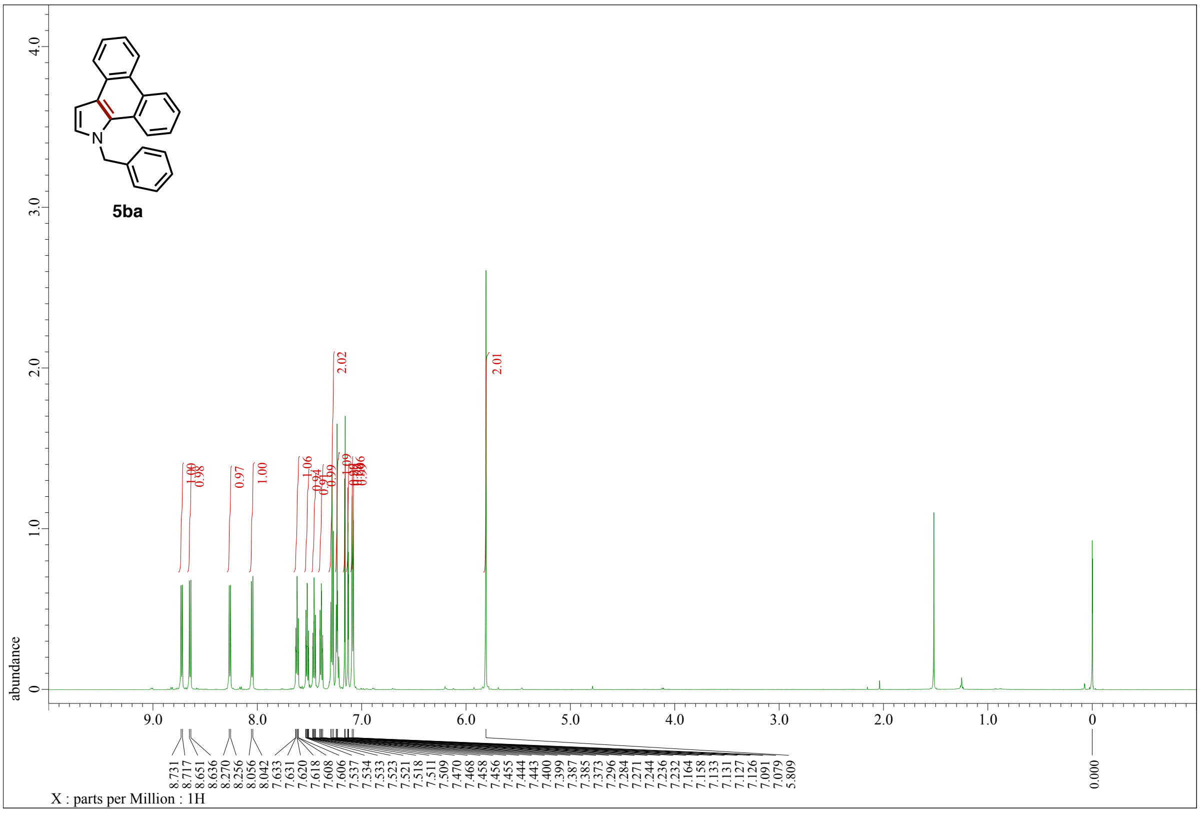

^{13}C NMR (150 MHz, $CDCl_3$)

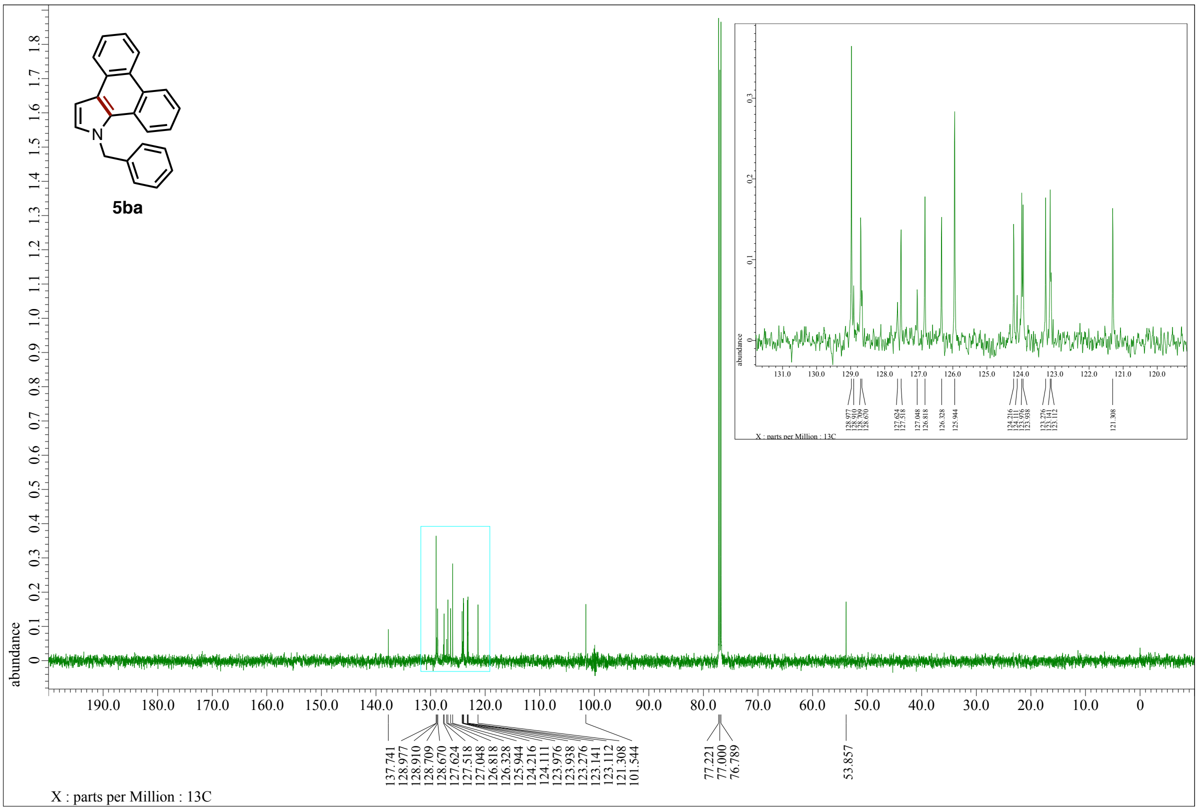

2-Chloro-9-methyl-9*H*-dibenzo[*a*,*c*]carbazole (3ab) and 7-chloro-9-methyl-9*H*-dibenzo[*a*,*c*]carbazole (3ab′)

^{1}H NMR (600 MHz, CDCl$_3$)

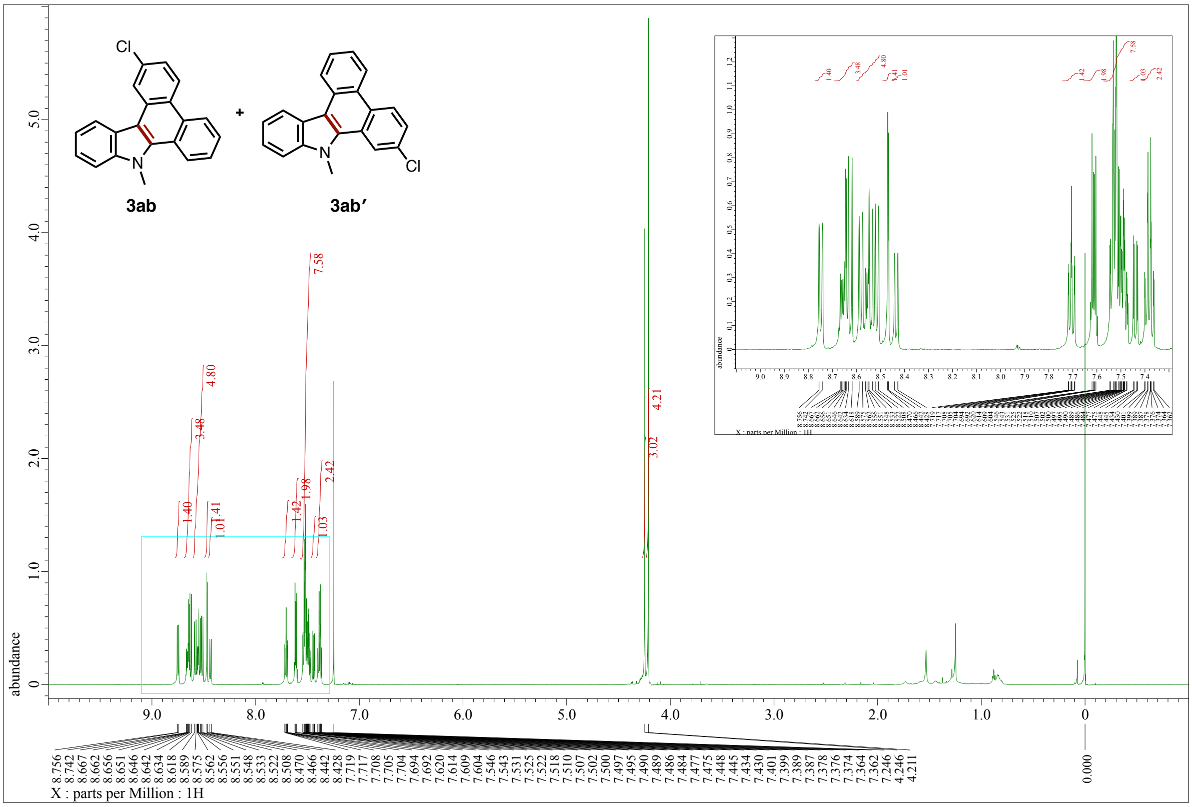

^{13}C NMR (150 MHz, CDCl$_3$)

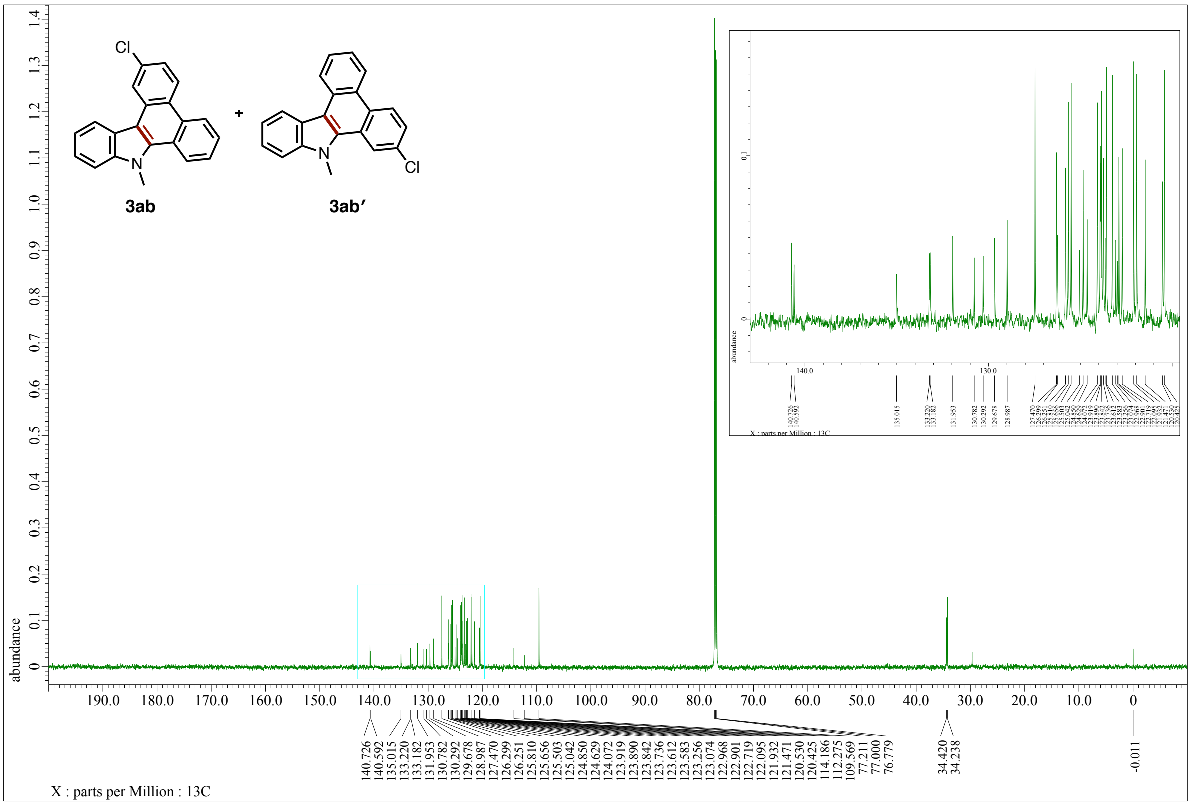

2,7-Dibromo-9-methyl-9*H*-dibenzo[*a*,*c*]carbazole (3ac)

^{1}H NMR (600 MHz, $CDCl_3$)

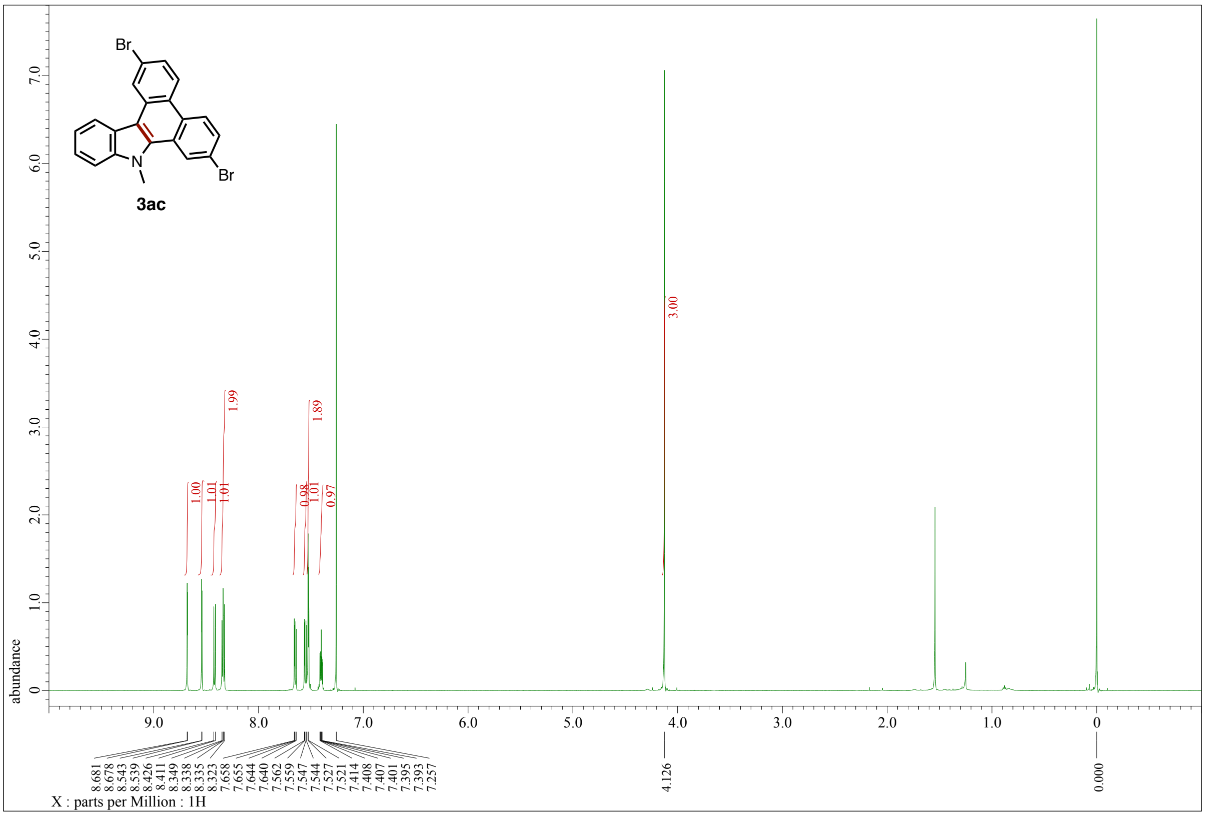

^{13}C NMR (150 MHz, $CDCl_3$)

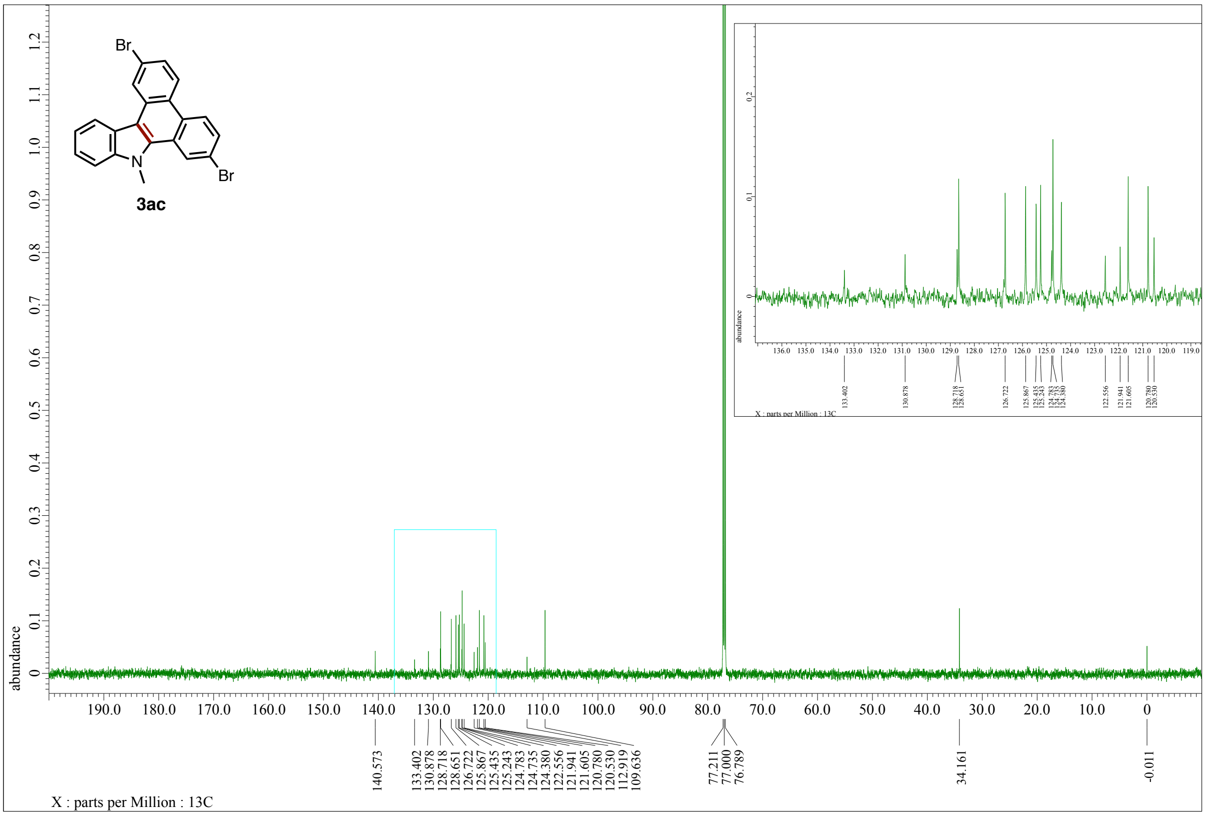

5,10-Dibromo-1-methyl-1*H*-dibenzo[*e*,*g*]indole (5cc)

^{1}H NMR (600 MHz, $CDCl_3$)

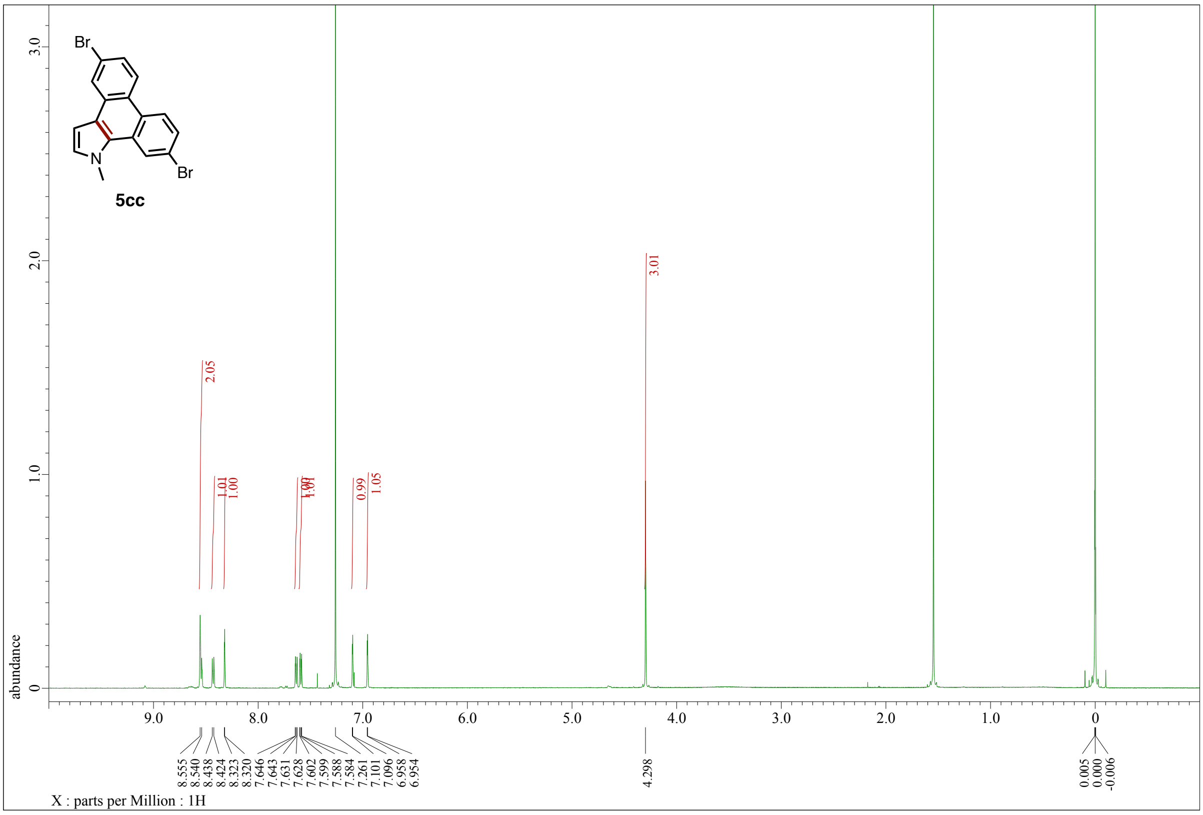

^{13}C NMR (150 MHz, $CDCl_3$)

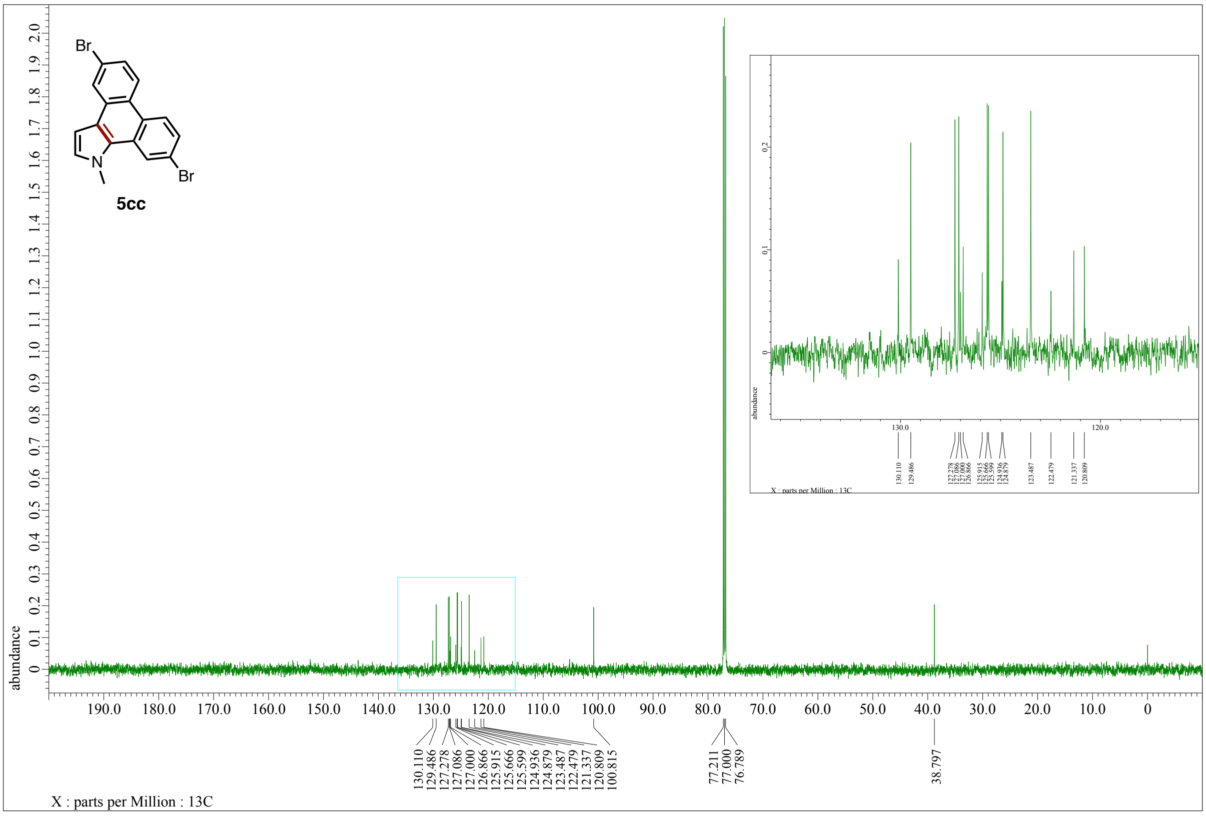

11-Methyl-11*H*-dinaphtho[2,1-*a*:1′,2′-*c*]carbazole (3ad)

^{1}H NMR (600 MHz, $CDCl_3$)

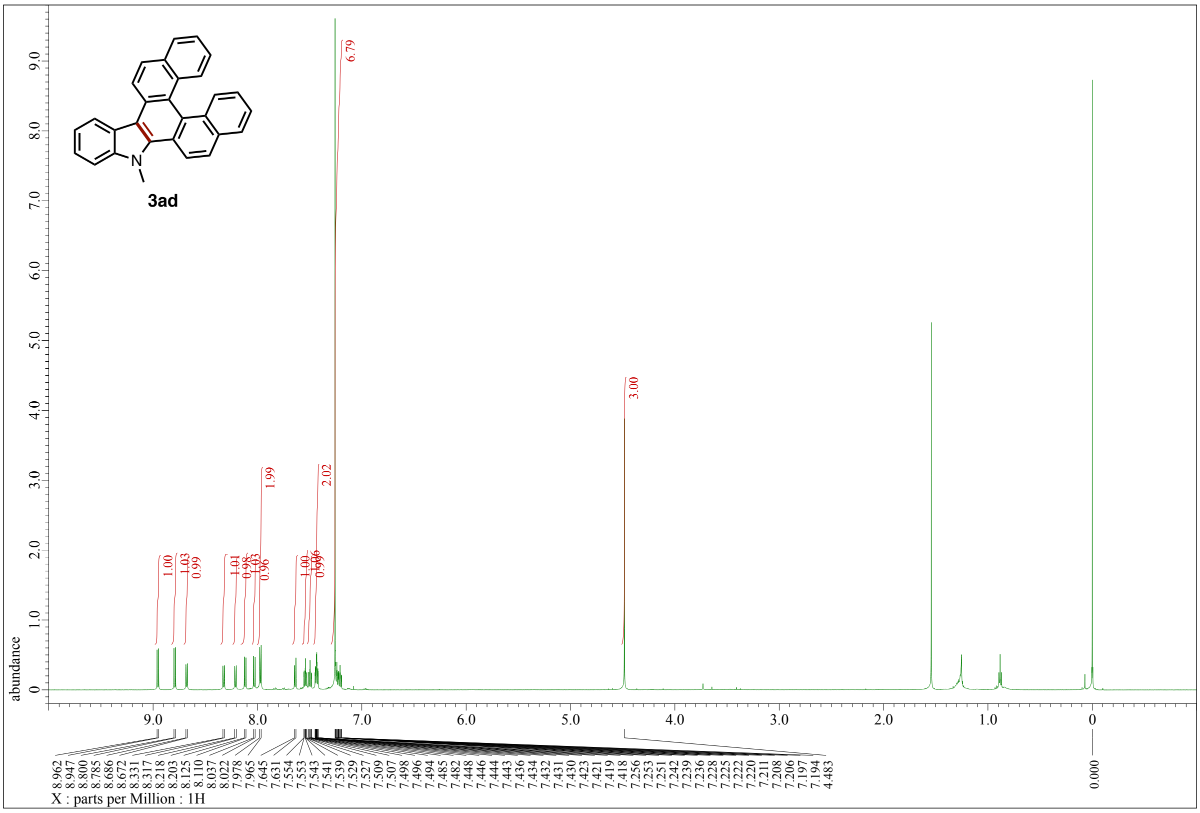

^{13}C NMR (150 MHz, $CDCl_3$)

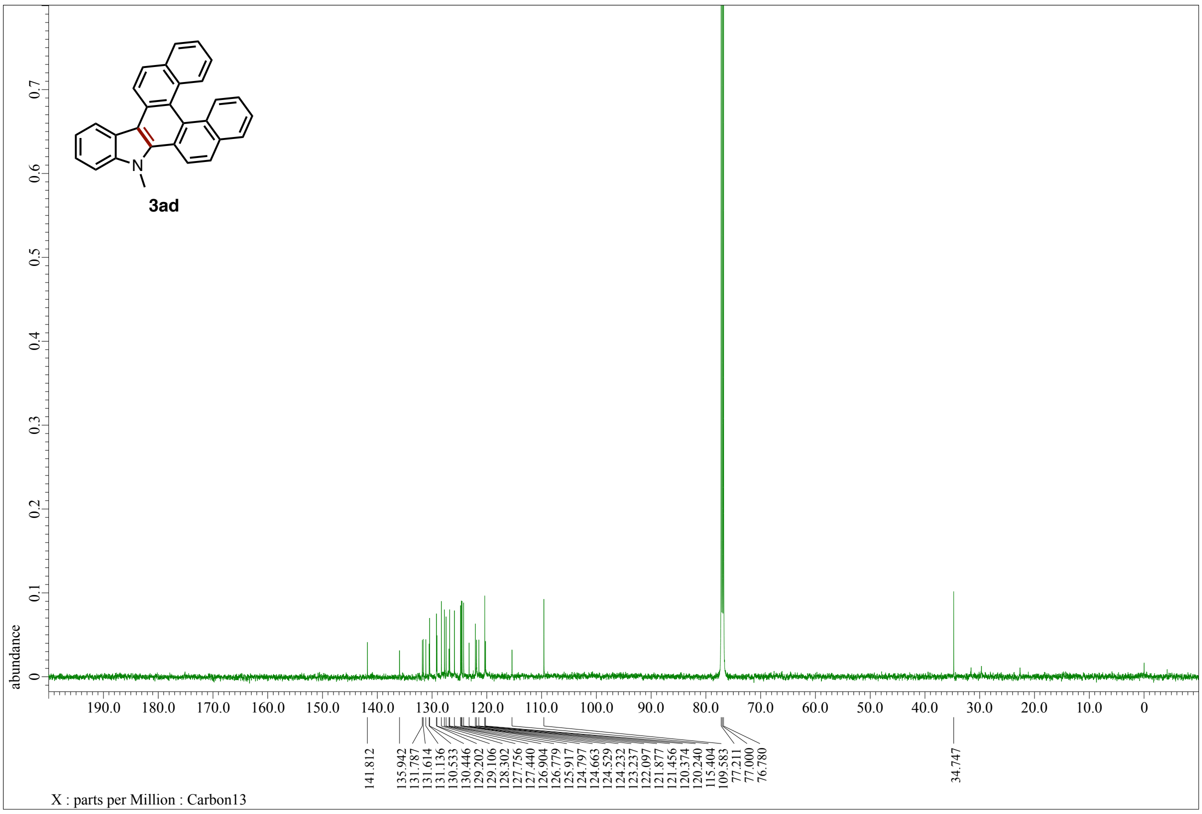

1-Methyl-1*H*-dibenzo[*e*,*g*]indole (5ca)

^{1}H NMR (600 MHz, $CDCl_3$)

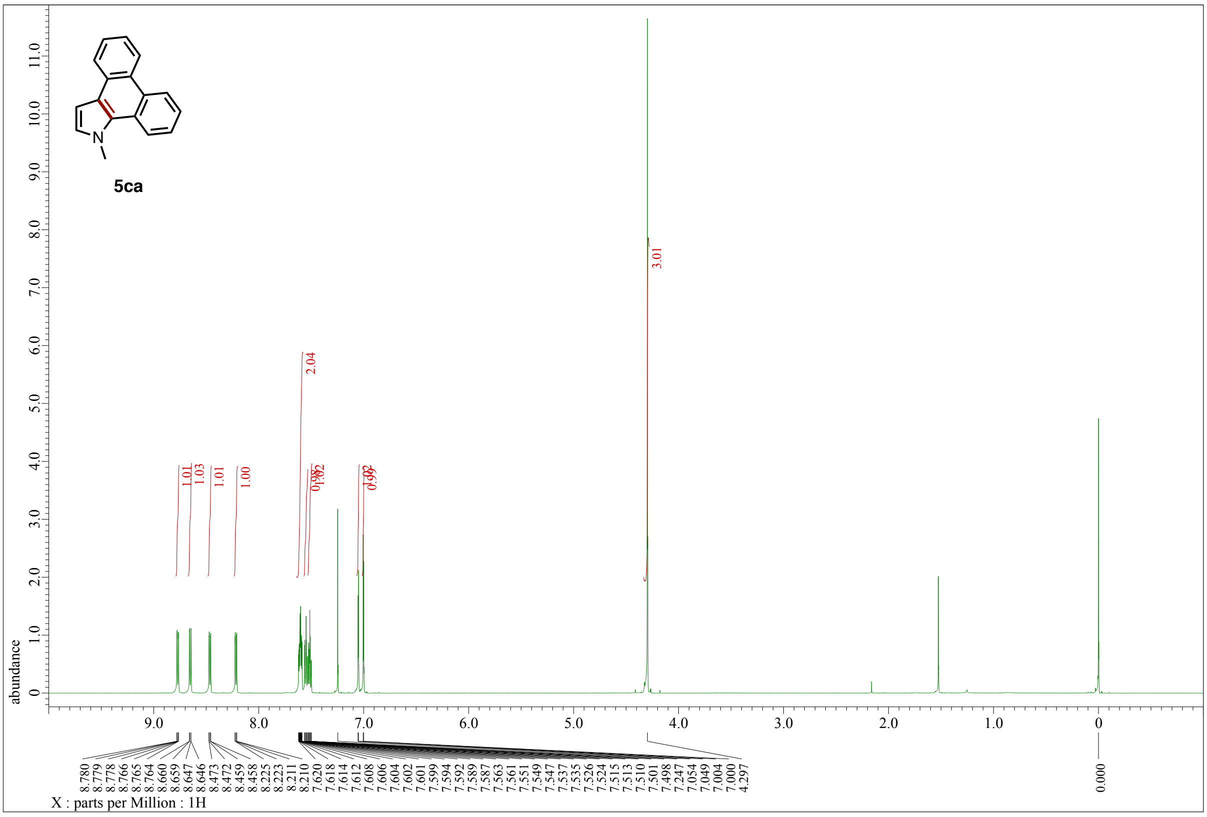

^{13}C NMR (150 MHz, $CDCl_3$)

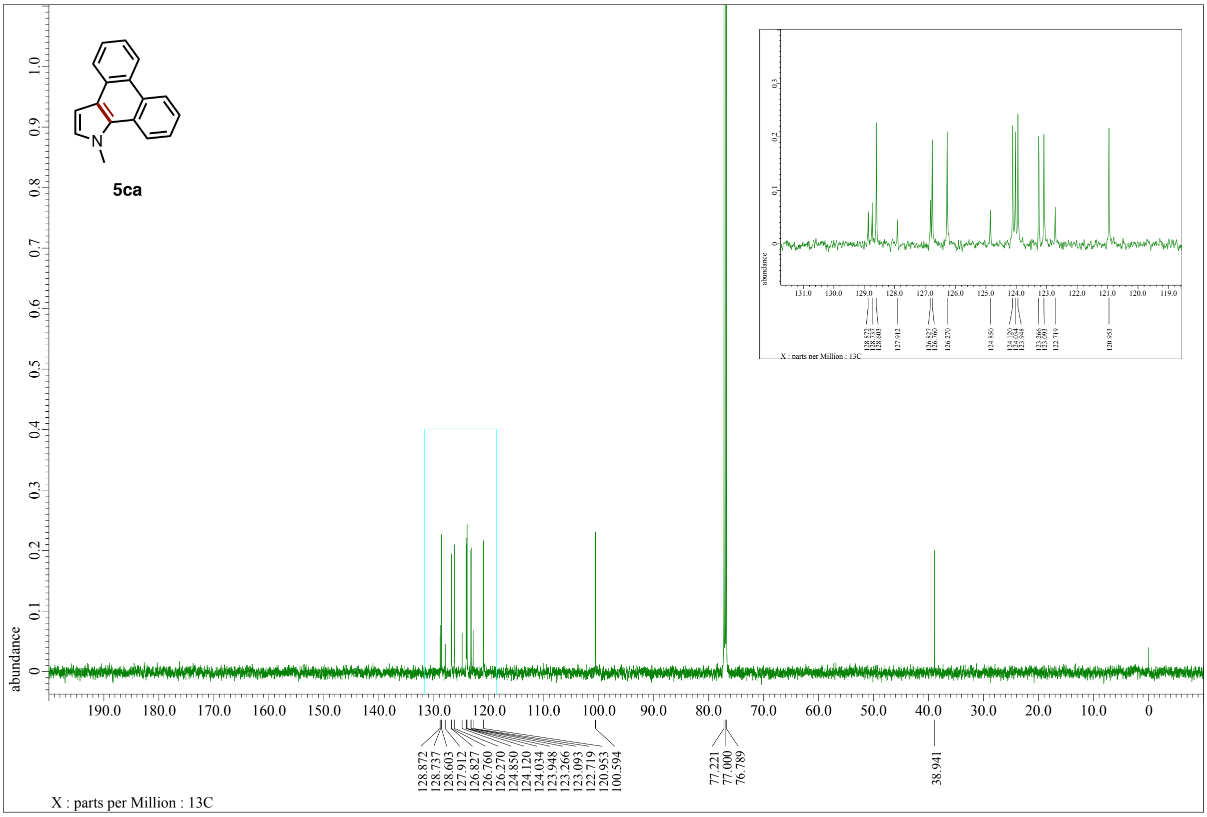

2,7-Dibromo-9-methyl-9*H*-tetrabenzo[*a*,*c*,*g*,*i*]carbazole (6)

^{1}H NMR (600 MHz, $CDCl_3$)

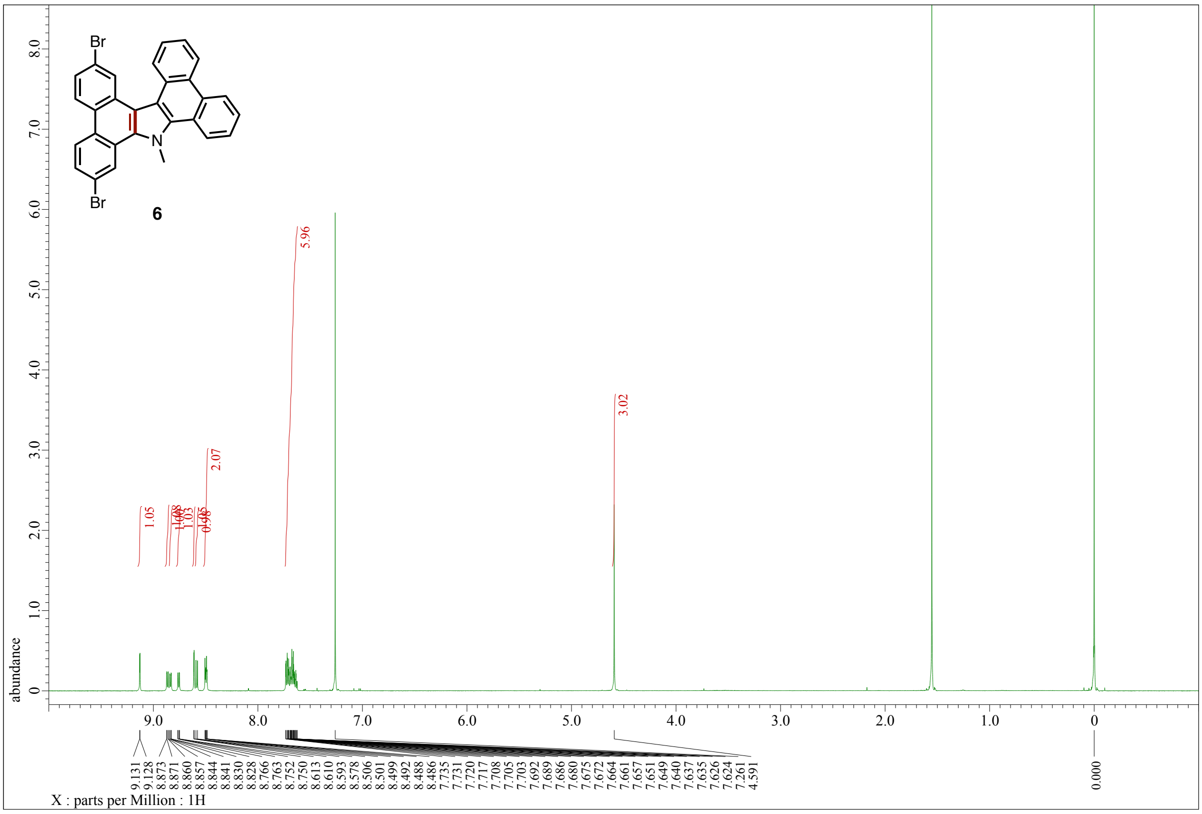

^{13}C NMR (150 MHz, $CDCl_3$)

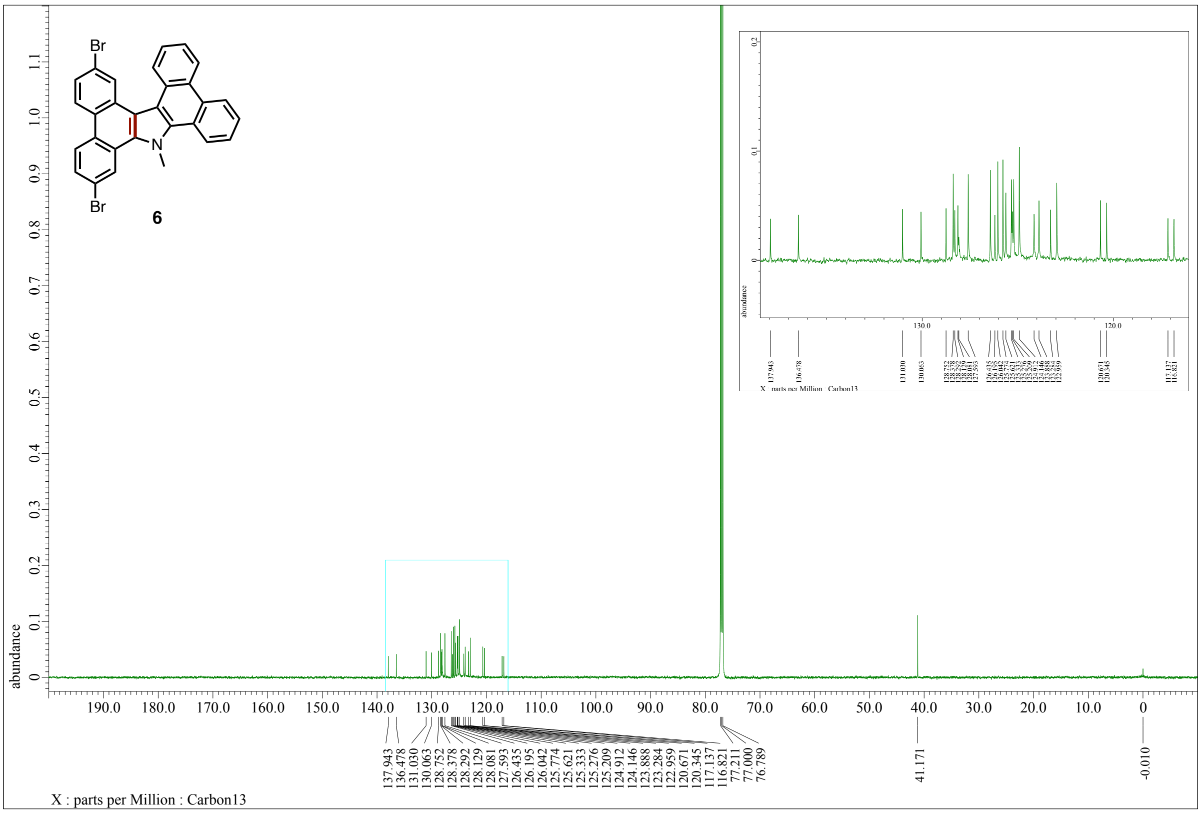

11-Methyl-11*H*-benzo[4,5]thieno[3,2-*a*]benzo[4,5]thieno[2,3-*c*]carbazole (8)

^{1}H NMR (600 MHz, $CDCl_3$)

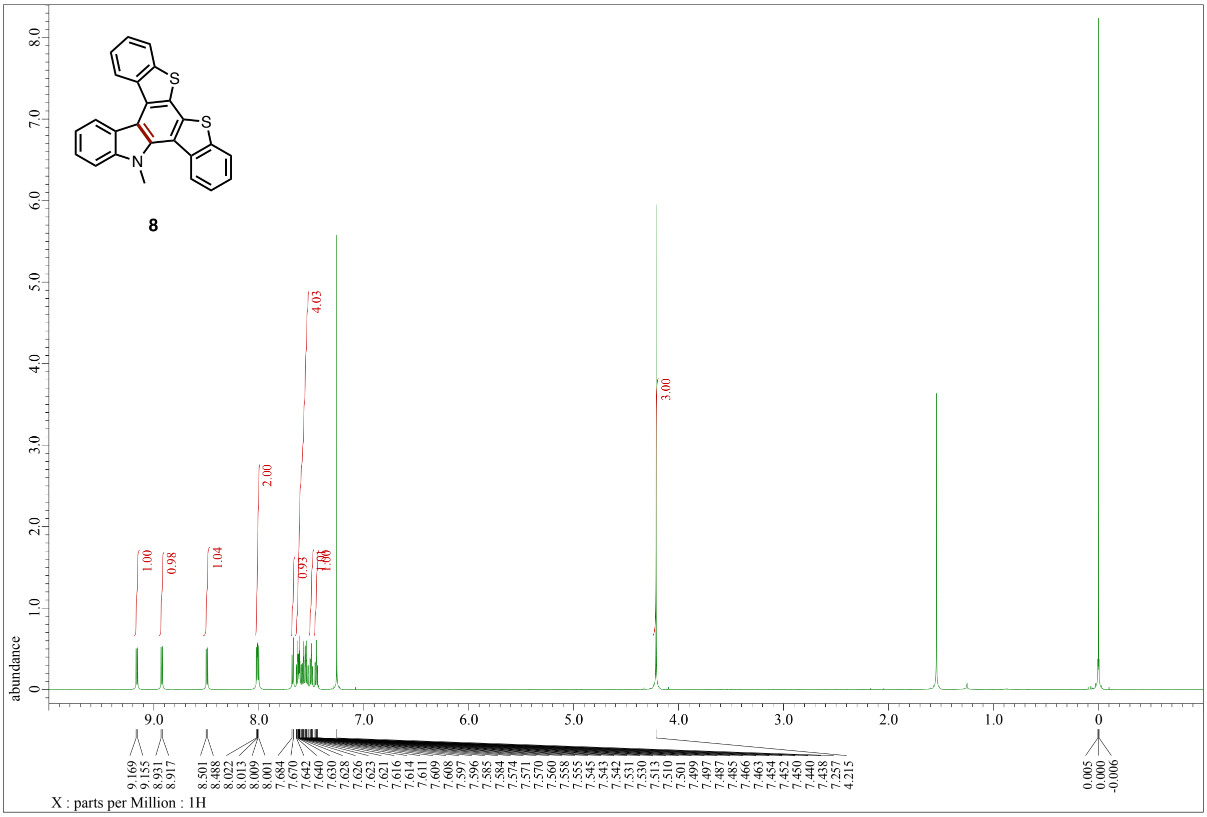

^{13}C NMR (150 MHz, $CDCl_3$)

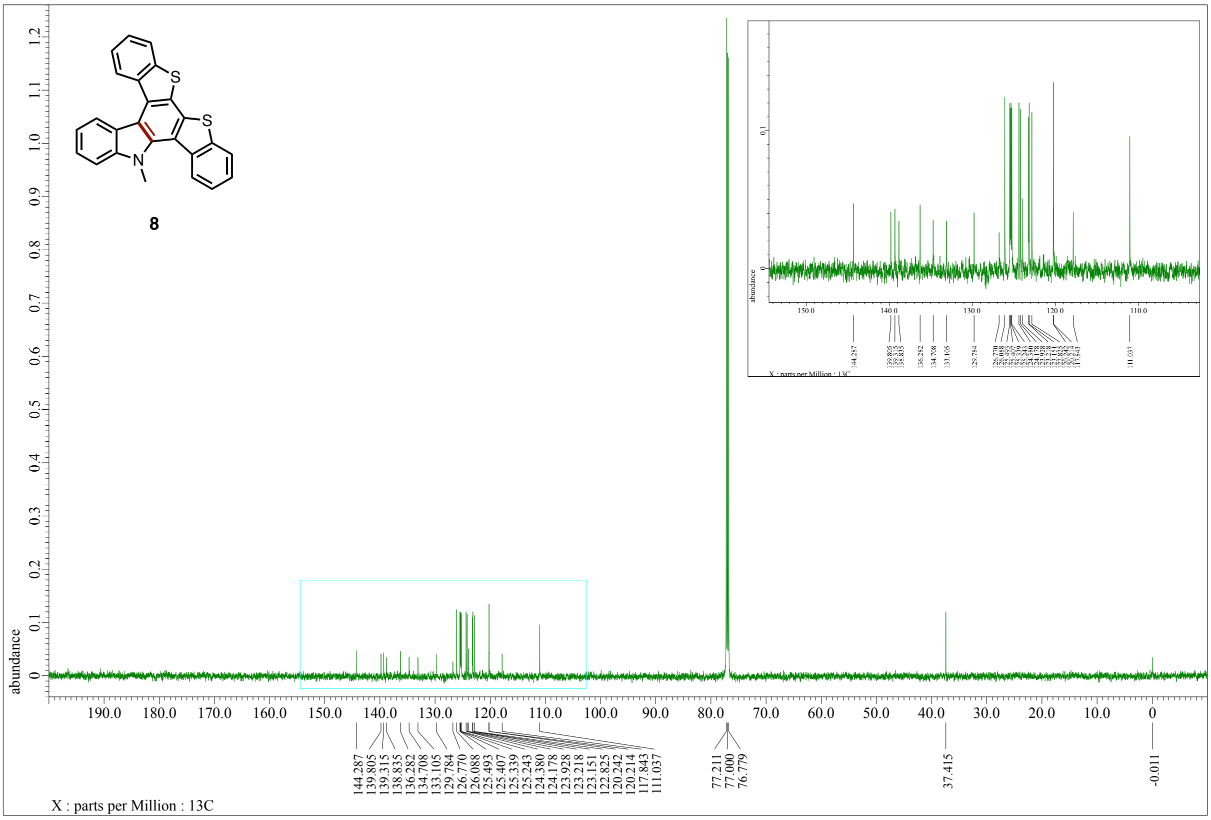

21-Methyl-21*H*-benzo[4,5]thieno[3,2-*a*]benzo[4,5]thieno[2,3-*c*]benzo[4,5]thieno[3,2-*g*]benzo[4,5]thieno[2,3-*i*]carbazole (9)

^{1}H NMR (600 MHz, $CDCl_3$)

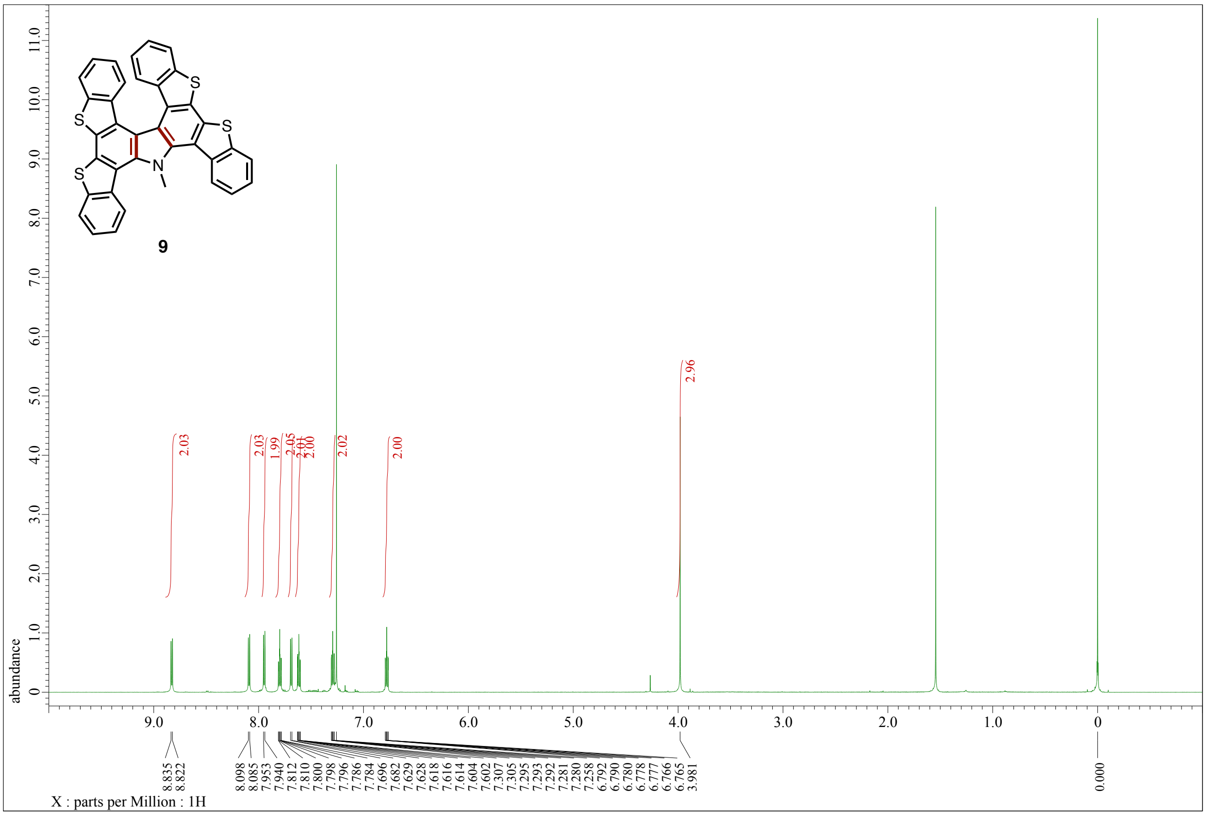

^{13}C NMR (150 MHz, $CDCl_3$)

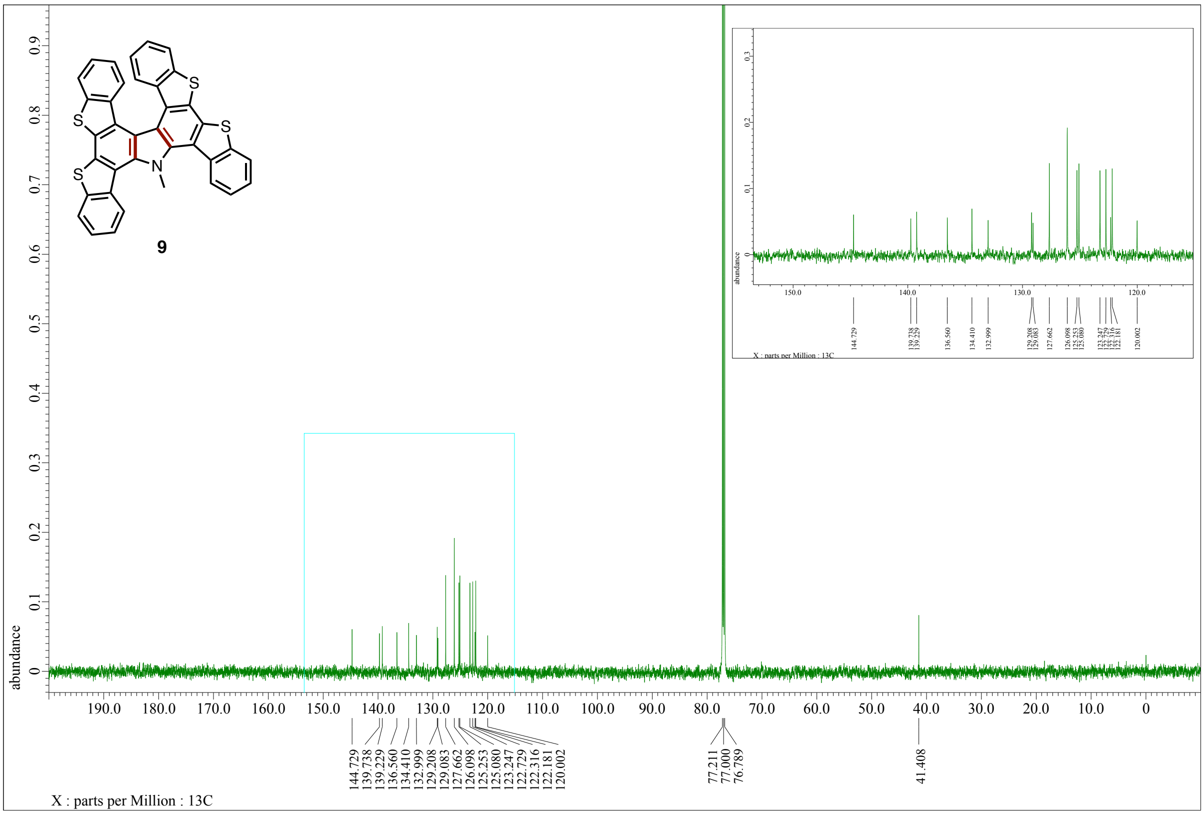

1,2-Dimethyl-3-phenyl-1*H*-indole (12)

^{1}H NMR (600 MHz, $CDCl_3$)

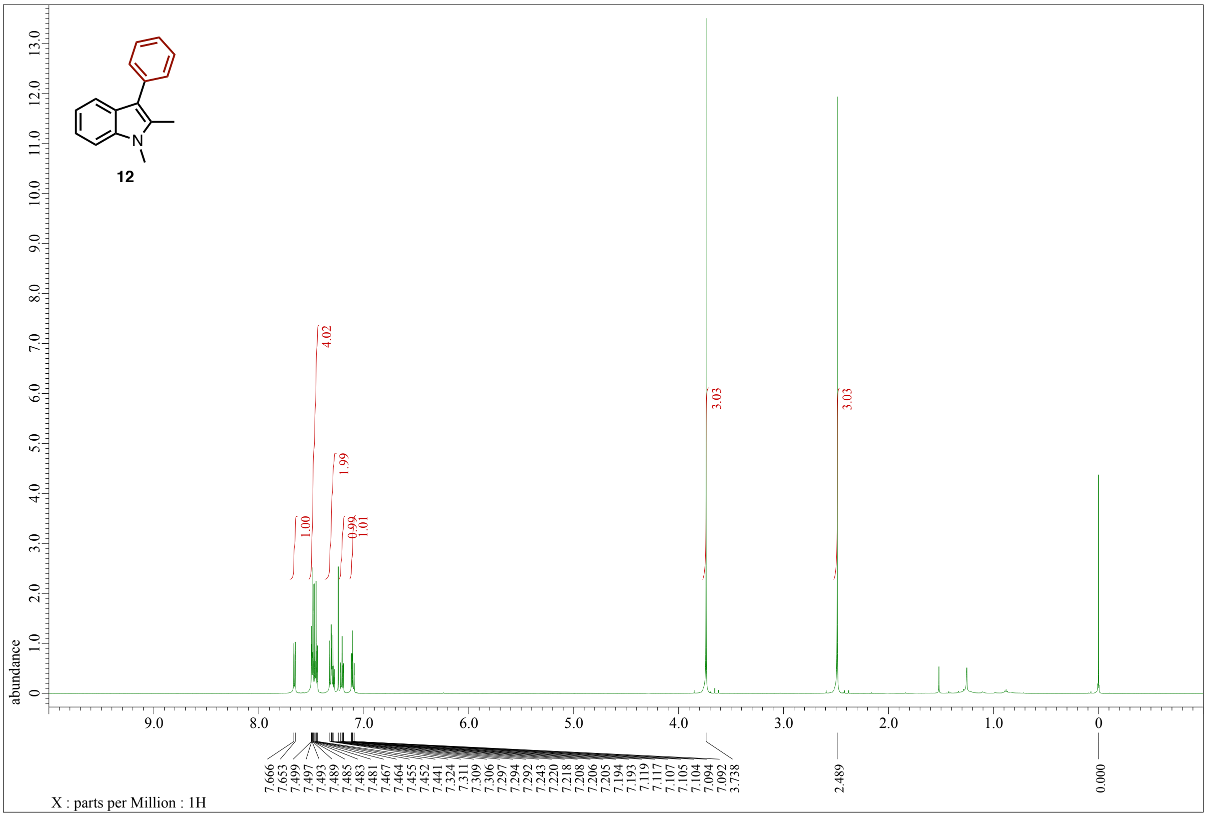

^{13}C NMR (150 MHz, $CDCl_3$)

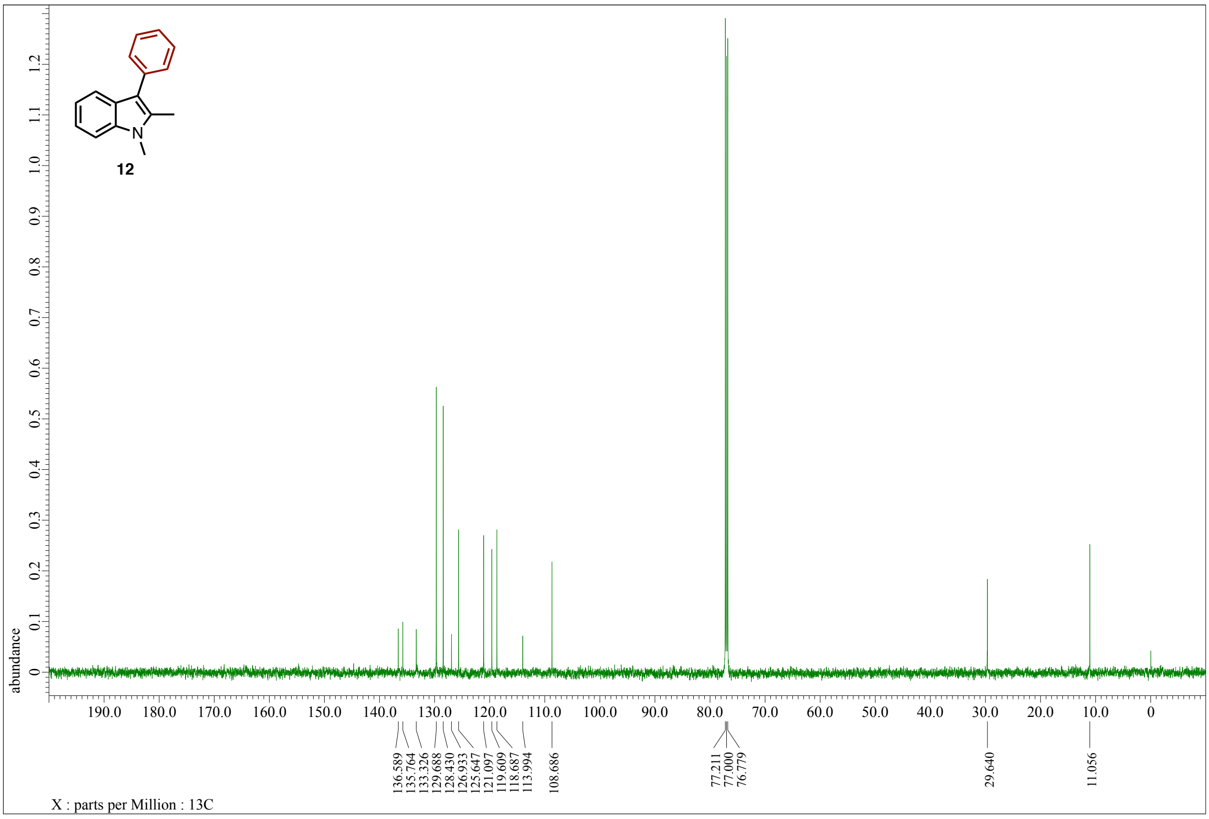

1,3-Dimethyl-2-phenyl-1*H*-indole (13)

^{1}H NMR (600 MHz, $CDCl_3$)

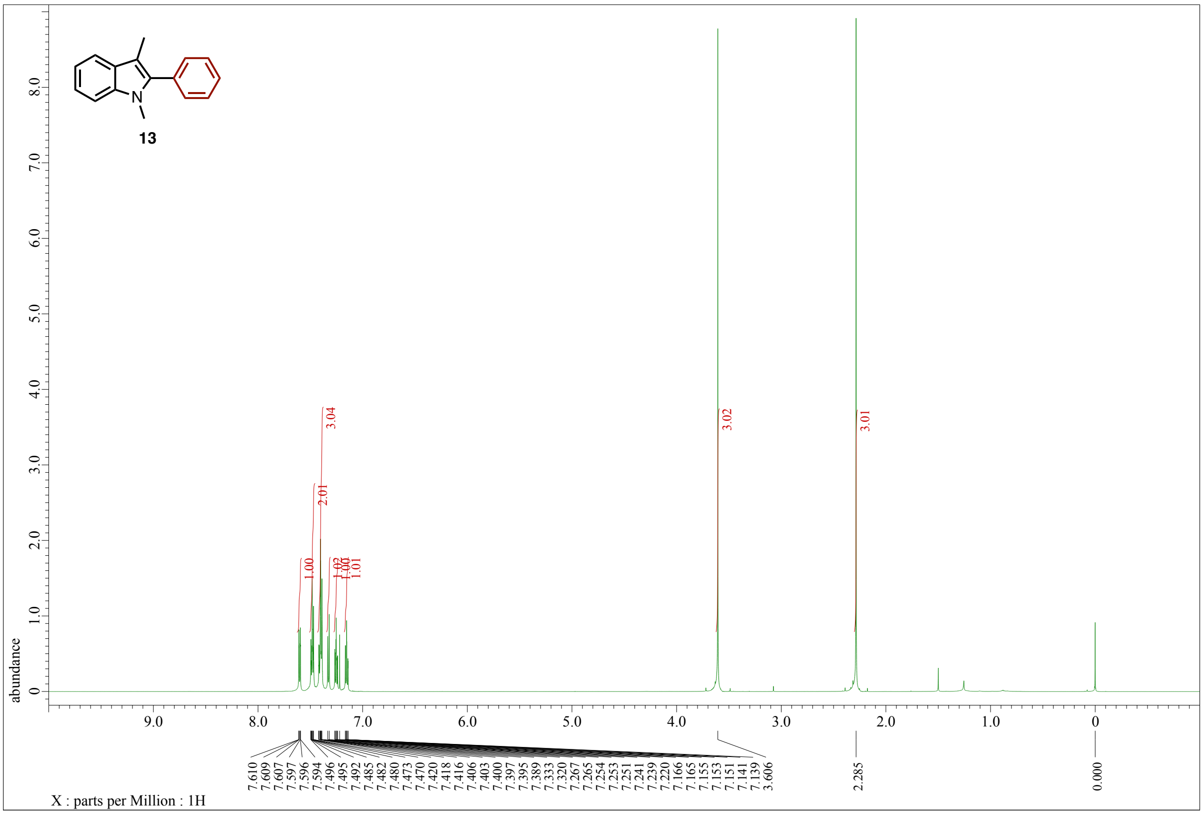

^{13}C NMR (150 MHz, $CDCl_3$)

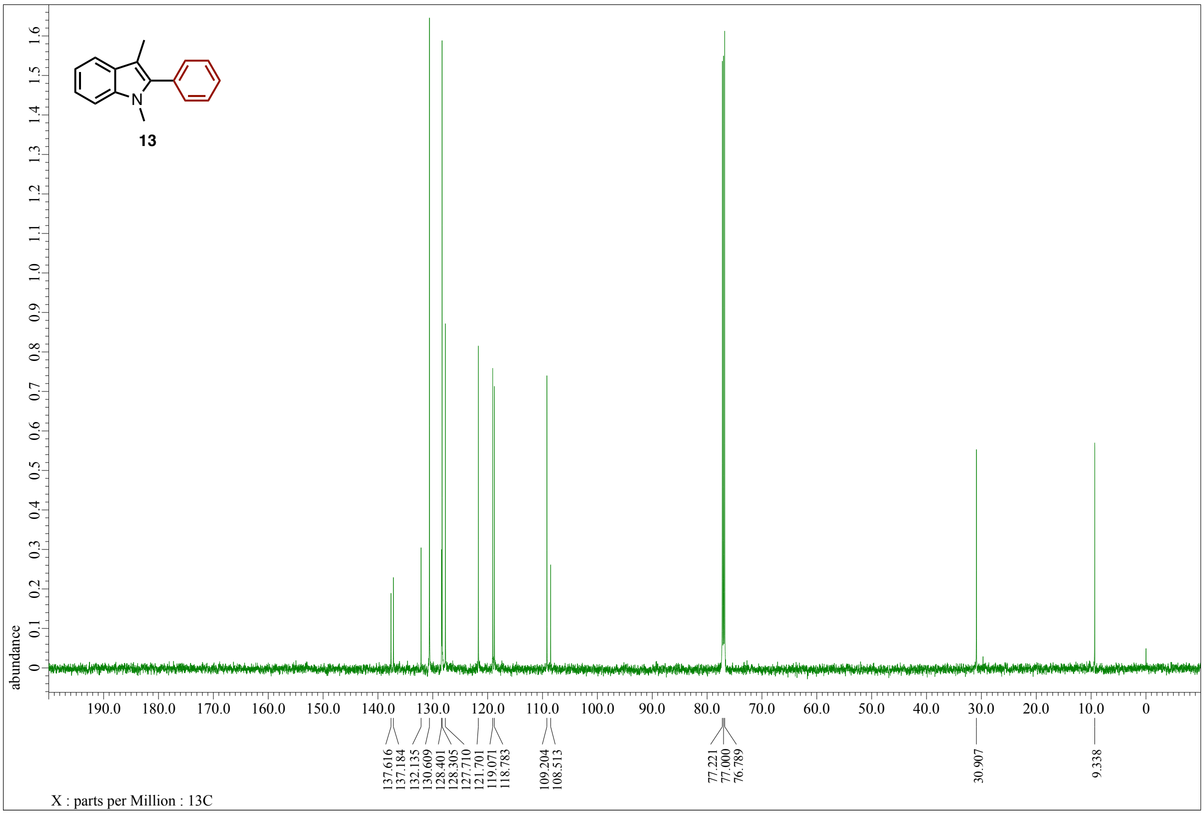

Supplement: Supplementary file 1 [file SC-009-C8SC02802H-s001.pdf]
